# Synthesis, Characterization and Biological Activity of Novel Cu(II) Complexes of 6-Methyl-2-Oxo-1,2-Dihydroquinoline-3-Carbaldehyde-4n-Substituted Thiosemicarbazones

**DOI:** 10.3390/molecules25081868

**Published:** 2020-04-17

**Authors:** Eswaran Ramachandran, Valentina Gandin, Roberta Bertani, Paolo Sgarbossa, Karuppannan Natarajan, Nattamai S. P. Bhuvanesh, Alfonso Venzo, Alfonso Zoleo, Mirto Mozzon, Alessandro Dolmella, Alberto Albinati, Carlo Castellano, Nuno Reis Conceição, M. Fátima C. Guedes da Silva, Cristina Marzano

**Affiliations:** 1 Department of Industrial Engineering, University of Padova, 35131 Padova, Italy; ramschemist@gmail.com (E.R.); roberta.bertani@unipd.it (R.B.); mirto.mozzon@unipd.it (M.M.); 2 Chemistry Research Center, National Engineering College, K. R. Nagar, Kovilpatti, Tamilnadu 628503, India; 3 Department of Pharmaceutical and Pharmacological Sciences, University of Padova, 35131 Padova, Italy; valentina.gandin@unipd.it (V.G.); alessandro.dolmella@unipd.it (A.D.); cristina.marzano@unipd.it (C.M.); 4 Department of Chemistry, Sri Ramakrishna Mission Vidyalaya College of Arts and Science, Coimbatore, Tamil Nadu 641020, India; 5 Department of Chemistry, Texas A&M University, College Station, TX 77842, USA; nbhuv@chem.tamu.edu; 6 Department of Chemical Sciences, University of Padova, 35131 Padova, Italy; alfonso.venzo@gmail.com (A.V.); alfonso.zoleo@unipd.it (A.Z.); 7 Department of Chemistry, University of Milan, 20133 Milan, Italy; alberto.albinati@unimi.it (A.A.); carlo.castellano@unimi.it (C.C.); 8 Centro de Química Estrutural, Instituto Superior Técnico, Universidade de Lisboa, 1049-001 Lisboa, Portugal; nunoconceicao@tecnico.ulisboa.pt (N.R.C.); fatima.guedes@tecnico.ulisboa.pt (M.F.C.G.d.S.)

**Keywords:** copper thiosemicarbazones complexes, X ray crystal structure, cytotoxicity in 3D spheroids, EPR and electrochemical measurements, TEM images

## Abstract

Three new 6-methyl-2-oxo-1,2-dihydroquinoline-3-carbaldehyde-thiosemicarbazones-N-4-substituted pro-ligands and their Cu(II) complexes (**1**, -NH_2_; **2**, -NHMe; **3**, -NHEt) have been prepared and characterized. In both the X-ray structures of **1** and **3**, two crystallographically independent complex molecules were found that differ either in the nature of weakly metal-binding species (water in **1a** and nitrate in **1b**) or in the co-ligand (water in **3a** and methanol in **3b**). Electron Paramagnetic Resonance (EPR) measurements carried out on complexes **1** and **3** confirmed the presence of such different species in the solution. The electrochemical behavior of the pro-ligands and of the complexes was investigated, as well as their biological activity. Complexes **2** and **3** exhibited a high cytotoxicity against human tumor cells and 3D spheroids derived from solid tumors, related to the high cellular uptake. Complexes **2** and **3** also showed a high selectivity towards cancerous cell lines with respect to non-cancerous cell lines and were able to circumvent cisplatin resistance. Via the Transmission Electron Microscopy (TEM) imaging technique, preliminary insights into the biological activity of copper complexes were obtained.

## 1. Introduction

**Chart 1:**
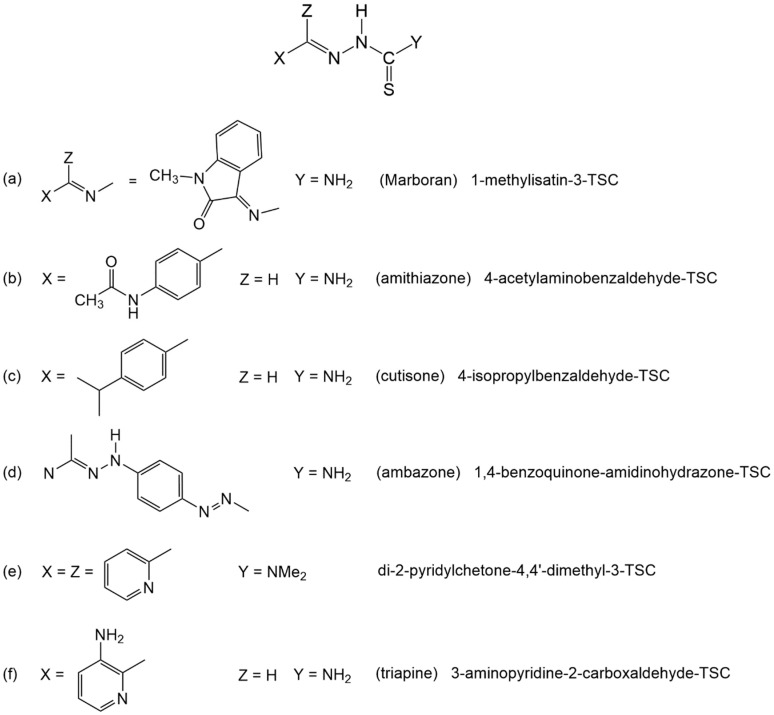
Thiosemicarbazones with known medicinal effects.

Great interest has been reserved in the last decades to the chemistry of thiosemicarbazones (TSC) of the type X-C(Z)=N-NH-C(=S)Y, where different combinations of X, Y and Z moieties have been introduced in the framework. This allows for the modulation of the lipophilic properties, chelating abilities and steric hindrance, also by the presence of additional functional groups, in order to attain an overall synergistic effect for their desired biological activity as such or that of their metal complexes [1,2,3,4,5,6,7,8,9,10,11,12]. A wide range of medicinal effects of thiosemicarbazones are well known (Chart 1): 1-methylisatin 3-TSC (Marboran, no longer produced as a drug) as being antiviral [13], 4-acetylaminobenzaldehyde-TSC (amithiazone, [14,15]) for the treatment of tuberculosis, 4-isopropylbenzaldehyde-TSC (cutisone [16]), 1,4-benzoquinone-amidinohydrazone-TSC (ambazone, [17]), di-2-pyridylketone-4,4′-dimethyl-3-TSC [18] and 3-amino pyridine-2-carboxaldehyde-TSC (Triapine, it has undergone Phase I and Phase II clinical trials, [19,20]) as potential antitumor drugs.

Recently, some copper-TSC complexes have been tested in vivo on HL60 human xenographs [21] and Ehlrich ascites carcinoma [22,23,24], showing a decrease in the weight loss and a significant increase in the percentage life-span of tumor-bearing mice.

The biological activity of TSC metal complexes is associated with the chelating ability of TSC ligands and their facility in inducing the perturbation of intracellular metal homeostasis (in particular of iron [11,25,26] and copper [27,28]). By forming redox active complexes, they generate reactive oxygen species (ROS) able to influence the activity of enzymes such as tyrosinase [29,30,31], topoisomerase [32], kinase [33,34], ribonucleotide reductase [35] or metalloproteinase [36].

Thiosemicarbazones can act as *NS* [37] bidentate, *NN′S* [38] and *ONS* [39] tridentate or *ONN′S* [40] tetradentate chelators, forming either neutral [41] or cationic [42,43], mononuclear [44,45] or polynuclear [46,47,48] systems with different metal ions. Complexes of V(IV) [49], Mn(II) [50], Fe(III) [38,51], Co(III) [52], Ni(II) [53,54,55,56], Cu(II) [28,57,58], Zn(II) [48], Ga(III) [59,60], Ru(II/III) [61,62,63,64,65,66,67,68,69], Pd(II) [70,71,72,73,74], In(III) [75], Re(I) [76,77], Pt(II) [78], Au(III) [79], Hg(II) [80], Ag(I) [81] and Sn(IV) [39] exhibit higher cytotoxicity and antimicrobial effects compared with the corresponding pro-ligands [82]. The anticancer activity of the metal complexes has been exploited to develop new compounds able to circumvent single and multidrug resistance [83]. In the attempt to achieve a structure/activity relationship, it was reported that for the TSC systems, the *NN′S* chelators exhibit a superior activity when compared to the *ONS* ones [1,28].

Quite recently, a Cu(II) hybrid system containing a polyoxometalate moiety and a 2-acetylpyrazine-TSC *NN′S* chelator was proven to exhibit antibacterial activity against *Escherichia coli* and *Staphilococcus aureus* and a better cytotoxicity against human hepatic cancer cells (SMMC-7721) than Mitoxantone, the current chemical anticancer drug, with an IC_50_ value of about 1.6 µg/mL [84].

In addition, bis(thiosemicarbazone) complexes of Cu(II) [85], Cu(I) [86] and Zn(II) [87] attracted great attention owing to their antiproliferative activity; recently, a wide series of dissymmetrically substituted bis(thiosemicarbazone) copper complexes, with different redox and lipophilic properties, have been investigated as radiopharmaceuticals for positron emission tomography [88].

The influence of different anions (i.e., NO_3_^−^ or SO_4_^2−^) on the TSC coordination ability for Cu(II) has also been investigated: it was observed that in the presence of the Cu(NO_3_)_2_·3H_2_O, binuclear systems could be obtained which exhibited an intriguing anticancer activity through mitochondrial apoptosis [89].

Some authors studied the coordination chemistry of TSC systems with different X moieties (Chart 1), observing that neutral or cationic complexes with Ligand/Metal ratios of 1:1 [65,72,90], 2:2 [91], 1:2 [74] or 2:1 [52,56] could be achieved, and their biological activities in terms of interactions with DNA via intercalation, antioxidant activity and cytotoxicity were investigated. For the water soluble 6-methoxy-2-oxo-1,2-dihydroquinoline–carbaldehyde thiosemicarbazone Ni(II) complexes bearing different NHR groups as the Y moiety (Chart 1), the biological activity depended on the nature of the R, and the system with the NHPh group had a cytotoxicity higher than that of cisplatin [92]. In the case of 2-oxo-1,2-dihydroquinoline-3-carbaldehyde thiosemicarbazone Cu(II) complexes, neutral compounds have been obtained when Y = NH_2_, NHMe or NHEt, whereas with Y = NHPh a cationic complex was achieved, the latter showing improved biological activity [93].

Here, we report the synthesis, characterization and biological activity of three 6-methyl-2-oxo-1,2-dihydroquinoline-3-carbaldehyde-TSC compounds and their Cu(II) complexes with the aim to evaluate the influence of X = C_6_H_4_CH_3_, compared with C_6_H_4_OCH_3_ analogues [90], and of Y = NHR (R = H,Me,Et) on their physical, chemical and biological properties.

## 2. Results and Discussion

### 2.1. Synthesis and Characterization of the Pro-Ligands

**Scheme 1:**
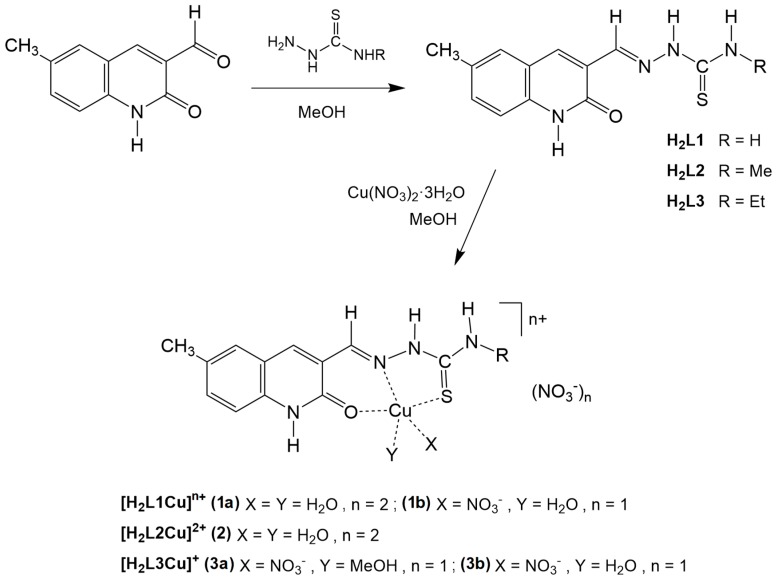
The synthetic route of the ligands and Cu(II) complexes.

H_2_L1, H_2_L2 and H_2_L3 have been prepared according to the procedure previously reported in the literature (Scheme 1) [63,67,92,94] by condensation of 6-methyl-2-oxo-1,2-dihydroquinoline-3-carbaldehyde [95] with the corresponding thiosemicarbazides in hot methanol. The compounds were obtained in high yields as yellow powders.

The absence of any ν(S-H) band in the 2700–2500 cm^−1^ region of the IR spectra excluded the presence of thiol species [96] and indicated the thione conformation for the compounds in the solid state. These results are in agreement with the theoretical calculations carried out on similar TSCs [97], which have shown that the thione tautomeric forms are stable, even in the presence of a solvent (MeOH). However, recent computational studies on 4-formylpyridine-TSC derivatives proved that both the thiol and thione forms are stable [98].

**Figure 1:**
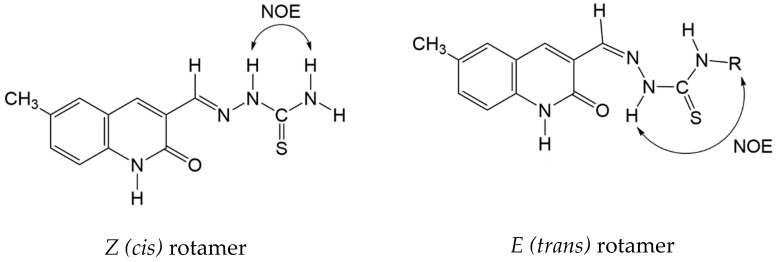
The *Z*(*cis)* and *E*(*trans)* rotamers of H_2_L1 (Y = NH_2_) and H_2_L2 (Y = NHCH_3_) (or H_2_L3, Y = NHCH_2_CH_3_) [97].

The ^1^H NMR spectra of H_2_L1 showed the presence of two distinct signals for –(C=S)N***H***_2_ protons at 8.09 and 8.31 ppm, respectively, the latter presenting a NOESY correlation with the N(2)***H*** proton at 11.64 ppm and suggested an *E* conformation. On the contrary, in the ^1^H NMR spectra of H_2_L2 and H_2_L3 a NOESY correlation between N(3)***H***···C***H***_3_ and N(2)***H***···C***H***_2_CH_3_, respectively, was observed, thus revealing different conformations in solution depending on the nature of the N(4) substituents [96] (Figure 1).

As for the mass spectrometric data collected under ESI conditions, the [H_3_L]^+^ and [H_2_L+Na]^+^ ions were detected for all the compounds, together with ionic species formed by the loss of NH_3_, H_2_NCH_3_ and H_2_NCH_2_CH_3_ from [H_3_L]^+^ (at m/z 244) and by the loss of H_2_S (at m/z 227, 241 and 255) from [H_3_L1]^+^, [H_3_L2]^+^ and [H_3_L3]^+^, respectively. It is worth noting that the latter ionic species are related to the metabolic pathways of TSCs compounds [3].

### 2.2. Synthesis and Characterization of the Complexes

The copper complexes **1**–**3** were synthetized by the direct reaction of Cu(NO_3_)_2_ × 3H_2_O with pro-ligand solutions. All the resulting mixtures gradually changed from blue to green, and by slow solvent evaporation dark green powder and crystals were obtained and characterized. The reaction of H_2_L1 with Cu(NO_3_)_2_.3H_2_O has been previously reported [99].

H_2_L1, H_2_L2 and H_2_L3 coordinated as *ONS* tridentate chelators in their neutral form lead to a square planar environment around the Cu ion where the fourth coordination site is engaged with a water (in **1a**, **1b** and **2**) or a methanol molecule (in **3a** and **3b**). The coordination geometry around the metal is completed by an additional water molecule (in **1a** and **2**), or one (in **3a** and **3b**) or two (in **1b**) nitrate anions, suggesting a possible facile substitution of the solvent ligands capable of promoting the interaction with a biological substrate.

It is worth noting that in the case of the reaction of 6-methyl-2-oxo-1,2-dihydroquinoline-3-carbaldehyde TSC with [PdCl_2_(PPh_3_)_2_] [100], the coordination mode depended on the nature of the Y substituents: tridentate *ONS* chelation when Y = NH_2_ (as in the present case) and bidentate *NS* chelation for Y = NHR (R = Me, Et, Ph).

The infrared spectra showed a decrease of the carbonyl stretching of the TCS ligand upon coordination, which was particularly evident for compounds **1** and **2**, together with a decrease of the C=S stretching vibration by 20 cm^−1^. The further appearance of bands attributed to Cu-O (523–525 cm^−1^) and to Cu-N (463–469 cm^−1^) confirmed the *ONS* coordination [101].

The mass spectra of the complexes revealed the presence of the [HLCu]^+^ ions as base peaks, together with oligomeric ionic species, as often observed under ESI conditions [58,102].

**Figure 2:**
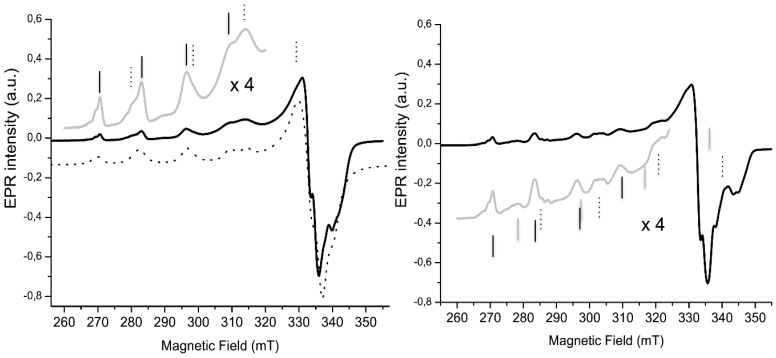
CW-EPR of the samples **1** (left) and **3** (right) in ethanol at 80 K. Black continuous line: experimental spectrum; dotted line: simulated spectrum of **1**; grey continuous line: the low-field region expanded to show all the features. The vertical lines mark the position of each quartet due to a different species.

The Continuous-Wave EPR (CW-EPR) spectra of **1** and **3** in the frozen matrix at 80 K are reported in Figure 2. The CW-EPR spectra of the Cu(II) complexes were characterized by a quartet of lines at low-field (the so-called parallel region), and a strong line at the higher field (perpendicular region). The parallel region was the most informative: the position of the quartet (related to the *g_zz_* parameter) and the distance between the lines (the *A_zz_* parameter) are very sensitive, even to small changes in the copper coordination. In agreement with the X-ray data, the spectrum of **1** (Figure 2, left) showed the occurrence of two detectable Cu(II) complexes with different coordinations. A well-matched simulated spectrum could be obtained, taking into account two Cu(II) species: one with *g_zz_* = 2.388 and *A_zz_* = 410 MHz (quartet marked by black vertical lines) and the other with *g_zz_* = 2.260, *A_zz_* = 520 MHz (quartet marked by dotted vertical lines). The latter presented a reduced *g_zz_* with an increased *A_zz_* with respect to the former, which was indicative of an increased covalent bonding and/or a decreased complex charge, in agreement with the X-ray determination that showed that the copper ion coordinated two water molecules in **1a** and one water molecule and one nitrate anion in **1b.** The EPR parameters for the first species (Table 1) were consistent with *ONS* donor Cu(II) complexes, and matched well with the complexes with two water molecules coordinated to the Cu(II) [103,104,105], and we could therefore assign it to **1a**. The second species had EPR parameters consistent with an *ONS* donor Cu(II) complex and could be attributed to **1b**. The g*_zz_*/A*_zz_* ratio has also been related to a deviation from square planar geometry [106], and this ratio was larger in **1a** than in **1b**, as revealed by the X-ray structure.

The EPR spectrum of **3** (Figure 2, right) showed a different pattern: apart from the presence of one species presenting very similar parameters to those of **1a** (quartet marked by black vertical lines) and another (marked by gray vertical lines) not very different from those found for **1b**, a third entity (in very small amounts) with a smaller *g_zz_* and larger *A_zz_* was also identified. Taking into consideration the resemblance of the EPR data and of the XRD structures, we could tentatively assign the species with parameters not very different from **1b**, to the entity **3b** (in fact, the g value was larger, in this case, but the g_zz_/A_zz_ ratio was similar), both actually presenting a similar coordination environment. However, the EPR parameters in the sample of **3** identical to those of **1a** could hardly be assigned to the XRD structure of **3a**, in view of the observed coordination distortion resulting from the steric influence of methanol. A tetrahedral Cu(II) configuration is easier in a crystalline or constrained environment, but it is unlikely to occur in solution, where Cu(II) prefers octahedral or square planar configurations. Since the EPR measurements were performed in ethanol, we could speculate that **3a** “relaxed” towards an octahedral or square planar structure (similar to **1a**). Further investigations through ENDOR will be carried out to clarify this point.

### 2.3. Electrochemical Properties

**Figure 3:**
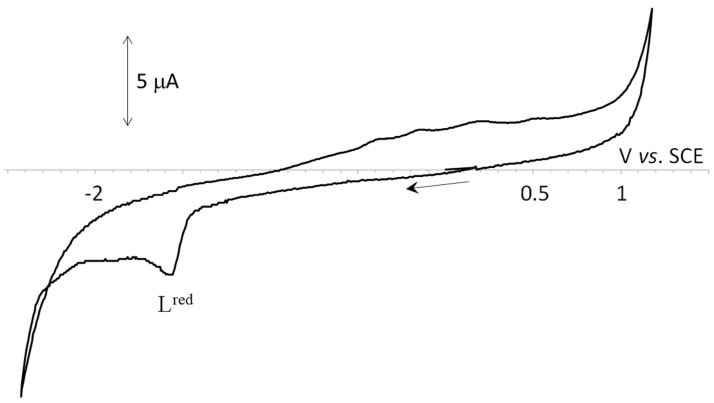
Cyclic voltammogram for H_2_L2 (C = 2.00 × 10^−3^ M) in [NBu_4_][BF_4_] 0.2 M in DMSO at a platinum disc working electrode (*d* = 0.5 mm) and at a scan rate of 0.2 V.s^−1^. The arrow indicates the cathodic initial scan direction.

The cyclic voltammetry studies were performed in DMSO/0.2 M [NBu_4_][BF_4_] solutions, at ambient temperature, in a three electrode cell and using a 0.5 mm diameter platinum disc working electrode. No oxidation waves were detected for H_2_L1-H_2_L3 pro-ligands in the available potential window, but they presented, at a scan rate of 0.2 V·s^−1^, an irreversible cathodic wave (wave L, Figure 3 and Figure S1 (in the Supplementary Material file); Table 2) at a potential value of −1.56 V vs. SCE (Table 2), which was insensitive to the structural differences (Y moiety, see Chart 1) of the compounds. No anodic processes were detected for H_2_L1-H_2_L3 in the available potential window.

**Figure 4:**
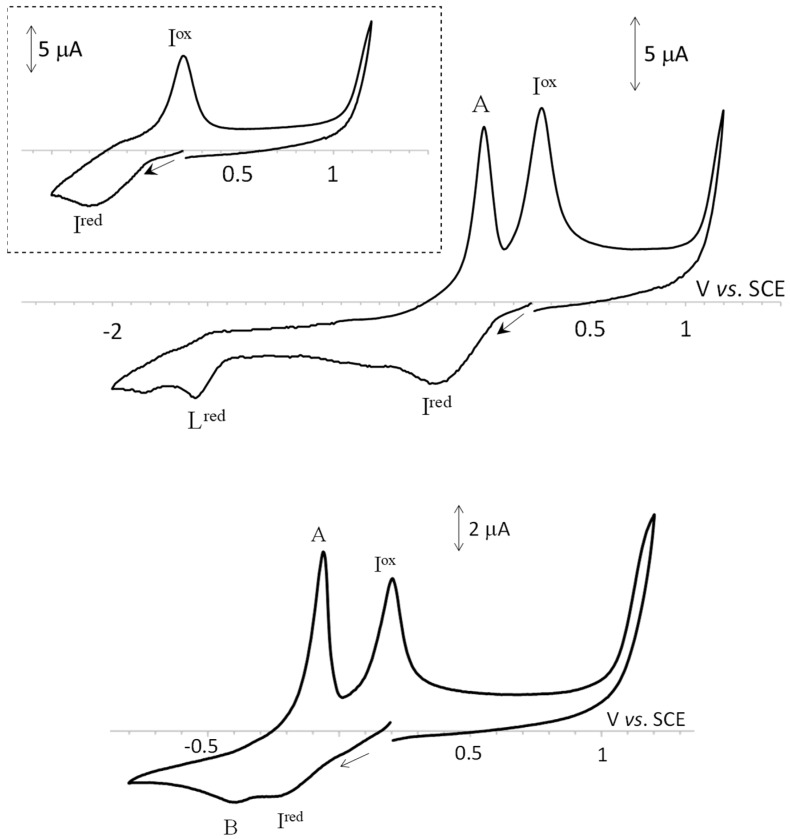
Cyclic voltammogram for complex **2** (C = 2.98 x 10^−3^ M) in [NBu_4_][BF_4_] 0.2 M in DMSO at a platinum disc working electrode (*d* = 0.5 mm), at 0.2 V·s^−1^ (top, including offset CV) and at 0.05 V·s^−1^ (bottom). The arrows indicate the cathodic initial scan direction.

Wave L^red^ was also detected in the derived Cu(II) complexes **1**–**3** at the same potential value (Figure 4 and Figure S2), therefore suggesting that the redox process was located far off from the influence of the metal center. In addition, the Cu(II) compounds presented, at the scan rate of 200 mV·s^−1^, a broad reduction wave (wave I^red^, Figure 4, top; Figure S2) between −0.26 and −0.32 V vs. SCE (Table 2), which was believed to involve the overlapped stepwise Cu^II^→Cu^I^ and Cu^I^→Cu^0^ reductions. Although at 0.2 V.s^−1^ the two cathodic processes were undifferentiated, they became evidently separated at lower scan rates (Figure 4, bottom), and a novel wave, B, (Figure 4, bottom) was detected at potentials of ca. −0.40 V vs. SCE.

Upon reversing the potential scan direction after the formation of wave L^red^, two anodic waves were observed, wave A and wave I^ox^ (Figure 4, top; Figure S2), assigned to the Cu^0^→Cu^I^ and Cu^I^→Cu^II^ oxidations, respectively. Such anodic waves (A and I^ox^) could also be detected upon reversing the potential scan direction after the formation of wave I^red^, provided the scan rate of the potential was sufficiently low, in particular for complex **2** (Figure 4, relate the top offset CV with the bottom one; compare with Figure S2). In view of the dicationic nature of compound **2**, its wave I^red^ potential was expected to be at a less negative value as compared to those of compounds **1** and **3**. The difficulty in rationalizing the redox potential values may be related not only to the broadness of this wave but also to the possible presence of different species in solution (see Section 2.2 and Section 2.3).

Our results contrast with those obtained for Cu(II) complexes with other TSC derivatives, for which only a cathodic wave corresponding to the quasi-reversible Cu^II^→Cu^I^ process was detected [107] at potential ranges from −0.68 to −0.49 V vs. SCE. Such reports contrast with our results, for which the stepwise reduction of Cu^II^ to Cu^0^ was observed at considerably lower negative potential values. This fact could be relevant from the biological point of view (see below). No rationalization between the cathodic and derived anodic potentials for **1**–**3** and the amine (Y) group could be achieved, in agreement with what was previously reported for other Cu(II) TSC compounds [108].

## 3. Crystal Structures of the Complexes 1, 2 and 3

Suitable crystals of the Cu(II) complexes of the ligands [**H_2_L1]** (complex **1**)**, [H_2_L2]** (complex **2**) and **[H_2_L3]** (complex **3**) were obtained by slow evaporation from diluted methanol solutions. The immediate coordination sphere around the Cu centers in all these complexes is the same with the substituted thiosemicarbazones acting as *ONS* tridentate ligands. However, the different substitutions on the TSC ligands (-NH_2_ in **1**, -NHMe in **2** and –NHEt in **3**) have an influence on the hydrogen-bond network and the resulting molecular packing. In all the complexes, the TSC ligand is flat (max. deviations from planarity based on the relevant lsq-planes ca. ± 0.03 Å). In two cases (compounds **1** and **3**), the asymmetric unit contains two independent molecules with different overall coordinations. In complex **2**, only one type of metal compound was located, so we start with its discussion. The metal oxidation state was the same in all three compounds, in agreement with the EPR data.

### 3.1. Solid State Structure of ***2***

**Figure 5:**
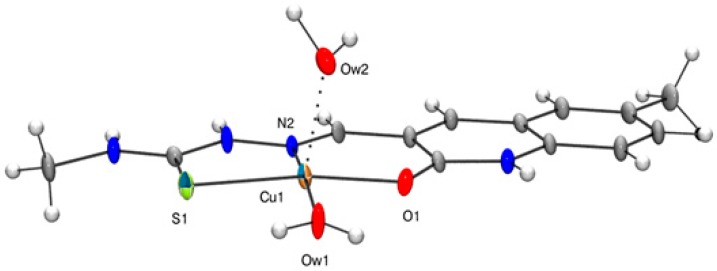
Ortep view of the cation in complex **2**. (Ellipsoids drawn at 50% probability; the numbering scheme used is just for comparison purposes in the following tables).

Figure 5 shows an Ortep view of **2**; selected bond lengths and angles are reported in Table 3, and an extended list of bond lengths and angles is given in Tables S5 and S6.

The presence of two NO_3_^−^ counterions unambiguously defines the metal oxidation state. The immediate coordination sphere consisted of the N, O, S atoms of the TSC ligand and the O atom of a water molecule (Cu-Ow1 1.928(3) Å); these ligands define a square planar geometry (Supplementary Materials, Table S7). A second clathrated H_2_O molecule (Cu-Ow2 2.226(4) Å) in an apical position resulted in a pseudo square-pyramidal environment. The Cu–ligand bond distances and angles are in the expected range and comparable with those of related structures [109].

There is a complex network of hydrogen bonds (Figures S4 and S6) involving the nitrate counter-ions as acceptors (A) and the O, N atoms of **2** as donors (D), with the D⋯A distances in the range 2.6–3.1 Å. Moreover, weak π-stacking interactions between the quinolinic rings could be observed (Figure S4), with *centroid*⋯*centroid* distances of ca. 3.8 Å.

### 3.2. Solid State Structure of ***1***

**Figure 6:**
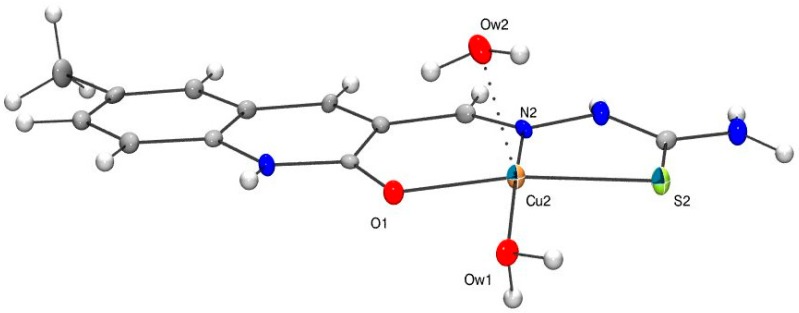
Ortep view of **1a**, one of the two independent cations in the unit cell of **1** (Ellipsoids drawn at 50% probability; the numbering scheme used is just for comparison purposes in the following tables).

**Figure 7:**
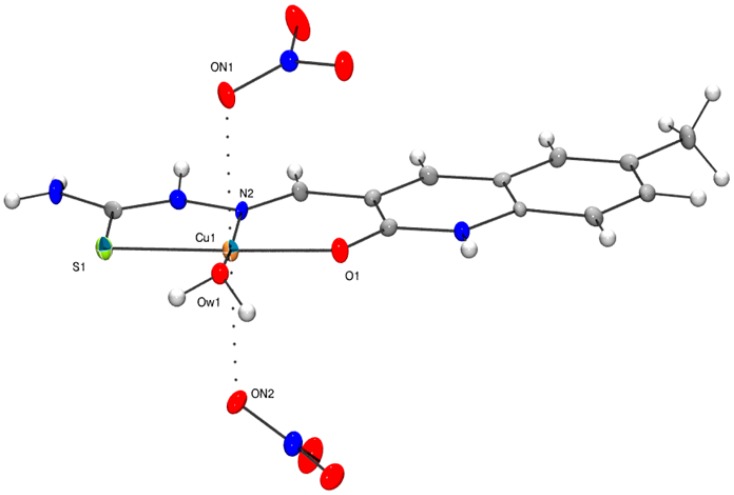
Ortep view of **1b**, of the second cation in **1** (Ellipsoids drawn at 50% probability).

The crystal structure determination revealed the presence of two independent Cu(II) cations in the unit cell (**1a** and **1b,** respectively), four nitrate ions and four clathrated water molecules. The immediate coordination sphere around the Cu center in **1a** and **1b** is identical to that found in **2** (Figure 6), with comparable distances and angles. However, while in **1a** even the weakly bound water molecule is present, in **1b** (Figure 7) there are weak interactions with oxygen atoms from two nitrates (2.695(4) Å and 2.525(4) Å) giving rise to a pseudo-octahedral copper environment. Moreover, the Cu-ON1 separation in **1b** (Cu–O 2.582(3) Å) was approximately equal to that found in the Cu–L complex (L = 2-oxo-1,2-dihydrobenzo[h]quinoline-3-carbaldehyde(2′-hydroxybenzoyl)hydrazine) [109], with a similar six-coordinated copper arrangement. The few small but statistically significant differences in bond lengths and angles (Table 3) around the two Cu centers (e.g., Cu–S at 2.266(2) Å in **2**, 2.289(2) Å in **1b** and 2.278(1) Å in **1a**) may be explained by the above-mentioned different overall environments and crystal packings. The intricate hydrogen-bond network (with donor⋯acceptor distances in the 2.6–2.9 Å range) involving the counter-ions and the O, N donors of the ligands is shown in Figures S10–S12. Due to the presence of the NH_2_ moiety in **1** acting as an H donor, the molecular packings here are quite different from those in **2** (Figure S11).

### 3.3. Solid State Structure of ***3***

**Figure 8:**
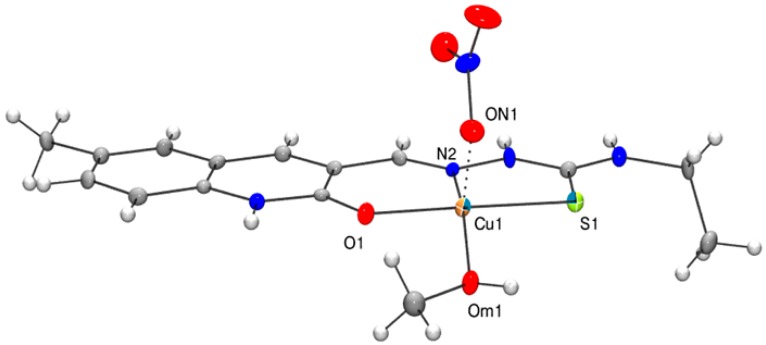
Ortep view of **3a** (Ellipsoids drawn at 50% probability; the numbering scheme used is just for comparison purposes in the following tables).

**Figure 9:**
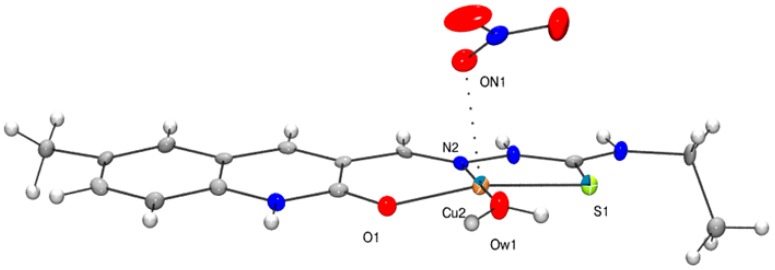
Ortep view of **3b** (Ellipsoids drawn at 50% probability).

The crystal structure determination revealed the presence of two independent molecules in the asymmetric unit (Figure 8 (**3a**) and Figure 9 (**3b**)), four nitrate ions and a clathrated water molecule. Selected bond lengths and angles are listed in Table 4; an extended list of bond lengths and angles is given in Table S10. In both cations, the metal centers are bonded to the three donor atoms of the HL3 ligand (i.e., S1, O1 and N2), while the fourth position of the slightly distorted square planar geometry is taken by a CH_3_OH molecule in **3a** and an H_2_O ligand in **3b**; a weakly interacting nitrate (at 2.318(3) Å and 2.388(3) Å, respectively) completed the coordination sphere. The bond lengths and angles involving the metal centers are in the expected range, comparable to those found in compounds **1** and **2**. The distances for the Cu-Om1 and Cu-Ow1 bonds fall at 1.989(3) and 1.931(3) Å, respectively. The Cu-Ow1 and Cu-ON1 separations in **3** are close to those found in the copper complex with 2-oxo-1,2-dihydrobenzo[h]quinoline-3-carbaldehyde(2′-hydroxybenzoyl)hydrazone (1.915(2) Å and 2.332(2) Å, respectively) [109]. The other bond lengths and angles are unexceptional. The differences in the geometrical parameters (Table 4) are easily accounted for by the different second-coordination sphere weak interactions or the aquo vs. methanol ligand coordination.

## 4. Biological Activity

The in vitro antiproliferative activities of the copper(II) complexes **1**–**3** and of the corresponding pro-ligands (**H_2_L1**, **H_2_L2** and **H_2_L3**) were evaluated against a panel of human tumor cell lines derived from solid tumors. The cytotoxicity parameters, expressed as IC_50_ (the median growth inhibitory concentration calculated from dose–survival curves), obtained after 72 h exposure, are listed in Table 5. Cell lines representative of colon (HCT-15), lung (A549) and pancreatic (BxPC3 and PSN-1) cancers, along with melanoma (A375), have been included. For comparison purposes, the cytotoxicity of cisplatin, the most widely used metal-based anticancer drug, was assessed under the same experimental conditions.

The uncoordinated ligands proved to be scarcely effective in decreasing the cancer cell viability (the average IC_50_ values were over 25 or 50 μM), whereas all Cu(II) complexes elicited IC_50_ values ranging from sub-micromolar to low micromolar levels (0.02–8.4 μM).

TSC Cu(II)complexes showed a higher potency than cisplatin against all the tested cancer cell lines. Compounds **2** and **3** showed a similar cytotoxicity profile, whereas **1** was endowed with the weakest in vitro antiproliferative effect. However, on average, complex **3** was the most effective derivative, showing IC_50_ values about five times lower than those calculated for cisplatin and about two times higher than those calculated for **1**. Notably, against human pancreatic cancer BxPC3 cells, complex **3** promoted a growth inhibitory effect about 46 times higher than that of cisplatin. It is worth noting that all Cu(II) complexes showed a prominent cytotoxicity towards human melanoma A375 cells. Tested against this cell line, complexes **2** and **3** elicited nanomolar IC_50_ values, attesting to a cytotoxicity roughly 56 times higher than that of the reference metal-based drug. For a comparison, the IC_50_ values against A549 cell lines for complexes **1**–**3** resulted in being much lower than those for related thiosemicarbazone complexes bearing Pd(II) [47], Co(III) [52] or Ni(II) [91], respectively.

The antiproliferative activity of the Cu(II) TSC complexes **1**–**3** was also investigated in two pairs of additional cell lines, including cisplatin-sensitive and -resistant human ovarian adenocarcinoma 2008/C13* cells, and oxaliplatin-sensitive and -resistant human colon carcinoma LoVo/LoVo-OXP cells. C13* cells and LoVo-OXP cells were suitably selected after a chronic treatment with the selected drug according to established protocols. Several mechanisms are known to cause the resistance to cisplatin of C13* cancer cells; these include (i) a reduced intracellular drug accumulation [110], (ii) high cellular levels of glutathione and metallothioneins [111], (iii) the enhanced repair of platinum-DNA adducts [112], and (iv) an overexpression of the Trx system [113]. On the other hand, oxaliplatin-resistant cells are mainly characterized by reduced drug accumulation and raised thiol levels whereas an increased tolerance to DNA platination seems to be less evident [114].

Cytotoxicity was assessed after 72 h of drug treatment by an MTT test, and the IC_50_ and RF values (RF = resistance factor, defined as the ratio of the IC_50_ of resistant cells over the IC_50_ of sensitive ones) are reported in Table 6. All new TSC Cu(II) complexes exhibited a similar cytotoxic potency both on sensitive and resistant cells, thus proving to be capable of overcoming both cisplatin and oxaliplatin resistance. Again, complex **3** was the most effective compound, showing in these cancer cell lines IC_50_ values in the low micromolar range.

As one of the main drawbacks of chemotherapeutic drugs is the possible toxic effect toward non-cancerous cells, we measured the cytotoxicity of **1**–**3** against non-cancerous cells (HEK293) and calculated the selectivity index (SI) by dividing the IC_50_ value in non-cancerous cells by that in the most sensitive A375 cancer cells. The cytotoxicity data for HEK293 cells and the selectivity indices are reported in Table 7. All TSC Cu(II) complexes showed IC_50_ values in the micromolar range against HEK293 non-cancerous cells. Despite the fact that a general selectivity towards cancer cells could not be attributed to complexes **1**–**3**, when considering A375 cancer cells a preferential cytotoxicity against tumor cells became evident. The SI _A375_ calculated for complexes **2** and **3** were roughly three-fold higher than that calculated for cisplatin, attesting to the preferential cytotoxicity of both complexes towards human melanoma cells.

The Cu(II) compounds **1**–**3** were also screened against 3D spheroids of colon cancer cells (HCT-15) and melanoma cells (A375). 3D cell cultures, comprising cancer cells in various cell growth stages, possess several features that more closely mimic the heterogeneity and complexity of in vivo tumors, being potentially more predictive for in vivo results than conventional 2D cell cultures [115]. The cancer spheroids were treated with Cu(II) complexes or cisplatin for 72 h, and the cell viability was assessed by means of the acid phosphatase (APH) assay (Table 8). Compounds **1**–**3** were markedly more effective than cisplatin against both 3D HCT-15 and A375 cultures. In particular, complex **2** was approximately eight- and four-fold more effective than cisplatin against HCT-15 and A375 cancer cell spheroids. These results clearly confirm the in vitro antiproliferative potential of these copper(II) TSC complexes.

**Figure 10:**
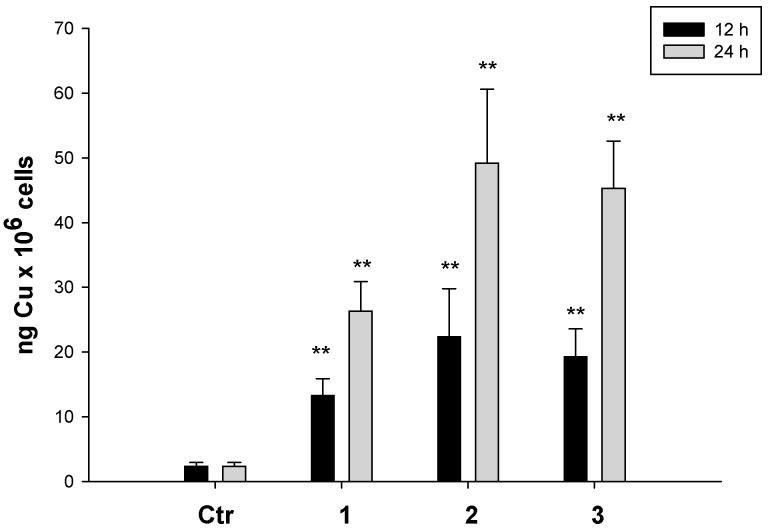
Cellular uptake in cancer cells. A375 cancer cells were incubated with 2 µM of copper complexes for 12 or 24 h, and the cellular copper content was detected by a GF-AAS analysis. Error bars indicate the standard deviation. ** *p* < 0.01.

As the biological activity of the compounds is determined by the extent of cellular accumulation, in the attempt to correlate the cytotoxicity potency with the ability of the tested complexes to enter cancer cells, we performed cellular uptake studies in A375 human melanoma cells. Cells were treated with 2 µM of copper complexes for 12 or 24 h, and the cellular copper content was quantified. The results, expressed as ng of Cu per 10^6^ cells, have been summarized in Figure 10.

All complexes were able to accumulate in a time-dependent manner, and compounds **2** and **3** were more efficient with respect to complex **1** in crossing the cellular periplasmic membrane. In addition, no saturation of the uptake was observed, therefore suggesting that carrier-mediated transport mechanisms can be excluded. Interestingly, by matching the cytotoxic activity values with the cellular uptake data recorded at 24 h, a direct and linear correlation was evidenced (R^2^ = 0.78, data not shown).

**Figure 11:**
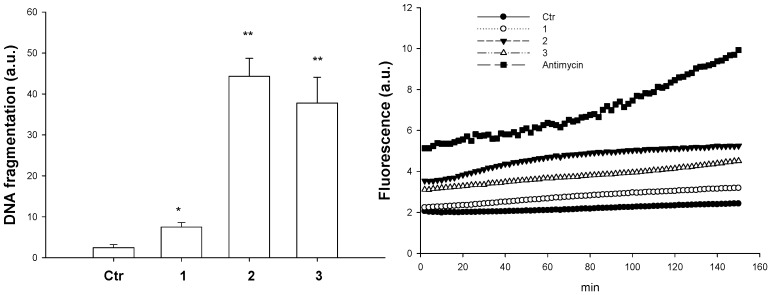
**Left**: DNA fragmentation. A375 cells were treated for 24 h with IC_50_ values of tested compounds and were then processed for nucleosome formation. The error bars indicate the S.D. **Right**: ROS production. A375 cells were pre-incubated in PBS/10 mM glucose medium for 20 min at 37 °C in the presence of 10 µM CM-H_2_DCFDA and were then treated with IC_50_ of compounds 1–3. The error bars indicate the S.D. ** *p* < 0.01; * *p* < 0.05.

Since the ′60s, DNA has been referenced as the main molecular target for various copper(II) complexes in many studies [28]. In addition, various TSCs have been recently recognized as DNA binding compounds [116]. On this basis, we evaluated the ability of Cu(II) complexes **1**–**3** to interact with DNA. Unfortunately, spectroscopic studies such as UV-titration and EB competition experiments with isolated DNA provided inconsistent results due to the very low solubility of tested complexes in Tris-HCl or other buffer systems. Similarly, DNA plasmid cleavage studies were hampered by the scarce solubility of tested complexes in serum-free media. Hence, the ability to induce DNA damage of compounds **2** and **3** was tested in intact cells by using the DNA damage ELISA kit. The results concerning A375 cancer cells treated with IC_50_ concentrations of **2** and **3** for 24 h have been reported in Figure 11 (left). A375 cancer cells treated with **2** and **3** showed a consistent increase (about 40% and 36%, respectively) in mono- and oligo-nucleosome formations, thus confirming the ability of the complexes to induce the fragmentation of nuclear DNA.

It has been widely reported that TSC copper complexes may catalyze hydrogen peroxide in the form of Fenton-like reactions inside the cell to produce ROS, thus altering cellular redox homeostasis and driving cells towards oxidative stress [117]. Based on this, we evaluated the intracellular levels of ROS in A375 cells after treatment with the copper complexes. As shown in Figure 11 (right), treatment of human melanoma cells with compounds **1**–**3** led to a very slight increase of the intracellular ROS accumulation. In particular, compound **2** was the most effective. The level of the generated ROS species was, however, significantly lower than that of antimycin, a classic inhibitor of the mitochondrial respiratory chain at the level of complex III.

**Figure 12:**
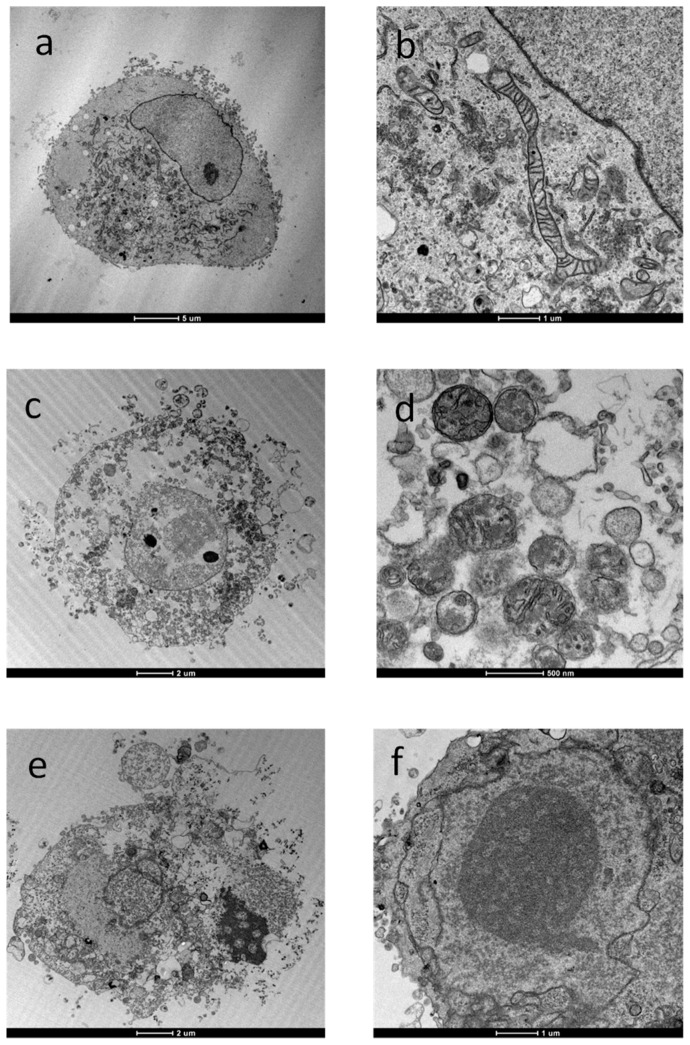
TEM analysis of A375 cells: (**a**,**b**): control cells; (**c**,**d**): A375 cells treated for 24 h with IC_50_ concentrations of **2**; (**e**,**f**): A375 cells treated for 24 h with IC_50_ concentrations of **3**.

Finally, in order to characterize the cellular morphological changes induced by the new Cu(II) TSC compounds, we observed treated A375 cancer cells using transmission electron microscopy (TEM), a valuable tool for investigating cells and their alterations at the ultrastructural level. Figure 12 shows representative TEM images of A375 cells after incubation for 48 h with IC_50_ doses of **2** and **3**. Cells treated with copper-TSC derivatives exhibited the characteristic ultrastructural features of apoptosis, such as cell shrinkage, chromatin condensation and formation of apoptotic bodies (Figure 12c–f with a,b control cells). However, mitochondria appeared to be quite conserved in terms of shape and internal structures, with minimal evidence of swelling features (increase in size and decrease in turbidity).

## 5. Materials and Methods

### 5.1. General

All reagents and solvents were commercially available and used without further purification. 6-methyl-2-oxo-1,2-dihydroquinoline-3-carbaldehyde was prepared according to the procedures previously reported in the literature [94].

The infrared spectra were obtained on a Spectrum 100 FT IR Spectrophotometer (Perkin-Elmer Italia, Milan, Italy) (KBr disks) in the range of 4000–400 cm^−1^; the wavenumbers ($\bar{v}$) are given in cm^−1^.

The electronic spectra of the complexes were recorded with a Jasco V-630 spectrophotometer (Jasco Europe, Milan, Italy) using DMSO as the solvent. Emission spectra were measured using a Jasco FP 6600 spectrofluorometer.

Elemental analyses (C, H, N, S) were performed on a Vario EL III Elementar analyzer instrument (Elementar Italia, Italy, Milan).

^1^H and ^13^C NMR spectra were obtained at 298 K on a AMX 600 NMR (Bruker, Milan, Italy) operating at 600.13 and 150.9 MHz, respectively; δ values (ppm) are relative to Me_4_Si for ^1^H and ^13^C. Suitable integral values for the proton spectra were obtained with a pre-scan delay of 10 s. The assignments of the proton resonances were performed by standard homonuclear chemical shift correlations (COSY, NOESY, TOCSY). The ^13^C resonances were attributed through 2D-heterocorrelated COSY experiments: the heteronuclear multiple quantum correlation (HMQC) with bilinear rotation decoupling [118] and quadrature along F1 were achieved using the time proportional phase increment method for the hydrogen-bonded carbon atoms, and the heteronuclear multiple bond correlation (HMBC) [119,120] for the quaternary ones.

The CW-EPR spectra were acquired on a ECS106 instrument (Bruker, Milan, Italy), equipped with a cryostat for low temperature measurements. The samples were prepared by dissolving the complexes **HL1Cu** and **HL3Cu** in ethanol (conc. 1 × 10^−3^ M), followed by quick freezing in liquid nitrogen. The acquisition parameters were: field sweep 70.0 mT, center field 305.0 mT, microwave power 743 μW, modulation amplitude 0.1 mT and microwave frequency 9.679 GHz.

Cyclic voltammetric studies were performed at ambient temperature on an EG&G PAR 273A potentiostat/galvanostat connected to a computer through a general-purpose interface bus (GPIB) interface. A three electrode cell was used, filled with 5 mL DMSO solutions of 0.2 M [NBu_4_][BF_4_] electrolyte, using a platinum disc (0.5 mm diameter) as the working electrode. A platinum auxiliary electrode was employed, and the potential was controlled vs. a Luggin capillary connected to a silver wire pseudo reference. Redox potential values were measured using ferrocene as the internal standard, for which the $E_{1/2}^{ox}$ value of 0.44 V vs. SCE was measured under the same experimental conditions, at 0.2 V·s^−1^, and using the saturated calomel electrode (SCE) as reference.

The ESI MS analyses were performed using a Finningan LCQ-Duo ion-trap instrument (Thermo Fischer Scientific, Milan, Italy), operating in positive ion mode (sheath gas flow N_2_ 30 a.u., source voltage 4.0 kV, capillary voltage 21 V, capillary temperature 200 °C). The He pressure inside the trap was kept constant. The pressure, directly read by an ion gauge (in the absence of the N_2_ stream), was 1.33 × 10^−5^ Torr. Sample solutions were prepared by dissolving the compounds (3 mg) in MeOH (5 mL) and diluting 1 mL of the solution in MeOH (5 mL) immediately before analysis.

### 5.2. Preparation of the Compounds

#### 5.2.1. Synthesis of 6-Methyl-2-oxo-1,2-dihydro-quinoline-3-carbaldehyde thiosemicarbazone (H_2_L1)

Thiosemicarbazide (0.91 g, 0.01 mol) dissolved in warm methanol (50 mL) was added to a methanol solution (50 mL) containing 6-methyl-2-oxo-1,2-dihydroquinoline-3-carbaldehyde (1.87 g, 0.01 mol). The mixture was refluxed for an hour, during which a yellow precipitate was formed. The reaction mixture was then cooled to room temperature, and the solid was filtered, washed with methanol and dried under vacuum. Yield 2.50 g (90%). MP: 248–250 °C. Elemental Analysis Anal. C 51.72%; H 4.97%, N 19.97%, S 11.41%. Calcd. for: C_12_H_12_N_4_OS·H_2_O (MW 278.33) C 51.78%, H 5.07%, N 20.13%, S 11.52%. UV-visible (DMSO; λ_max_, nm): 262, 352, 405 (л→л*, n→л*). IR (KBr, cm^−1^): 3435, 3164 (s) ν(NH); 3281 (s) ν(NH_2_); 1658 (s) ν(C=O); 1598, 1564 (s) ν(C=N) + ν(C=C); 845 (m) ν(C=S). ^1^H NMR (DMSO-d_6_, 600 MHz) δ 11.64 (1H, s, N(2)-H), 11.94 (1H, s, N(3)-H), 8.31 and 8.09 (2H, m, NH_2_), 8.29 (1H, s, C(1)-H), 8.71 (1H, s, C(6)-H), 7.24 (1H, d, J = 8.4 Hz, C(7)-H), 7.37 (1H,dd, J = 8.4 Hz, J = 1.8 Hz,C(8)-H), 7.43 (1H,d, J = 1.8 Hz, C(10)-H), 2.36 (3H, s, C(11)-H). ^13^C{^1^H} NMR (DMSO-d6, 600 MHz) δ 137.3 (C-1), 125.7 (C-2), 161.3 (C-3), 137.4 (C-4), 116.7 (C-5), 135.3 (C-6), 115.6 (C-7), 132.7 (C-8), 132.8 (C-9), 128.3 (C-10), 20.4 (C-11), 161.3 (C-12). ^15^N NMR (DMSO-d_6_, 600 MHz) δ 320 (s, N(1)); 173 (d, J = 95 Hz, N(2)H), 151 (d, J = 90 Hz, N(3)H), 109 (t, J = 90 Hz, N(4)H). ESI (H_2_L1 = C_12_H_12_N_4_OS, MW 260.31) m/z 261 [H_3_L1]^+^ (30), 283 [H_2_L1Na]^+^ (10), 244 [H_3_L1 - NH_3_]^+^ (20), 266 [H_2_L1Na–NH_3_]^+^ (55), 227 [H_3_L1-H_2_S]^+^ (40), 803 [3H_2_L1Na]^+^ (20), 845 [C_36_H_32_N_12_O_3_S_3_Na_3_]^+^(80), 915 [C_44_H_27_N_12_O_4_S_4_]^+^ (100).

#### 5.2.2. Synthesis of 6-Methyl-2-oxo-1,2-dihydroquinoline-3-carbaldehyde-4N-methylthiosemicarbazone (H_2_L2)

It was prepared using the same procedure as described for H_2_L1 with 4-methyl-3-thiosemicarbazide (1.052 g, 0.01 mol) and 6-methyl-2-oxo-1,2-dihydroquinoline-3-carbaldehyde (1.87 g, 0.01 mol). A yellow product was obtained. Yield 2.30 g (84%). MP: 261–263 °C. Elemental Analysis Anal C 56.83%, H 5.10%, N 20.28%, S 11.63%. Calcd. for C_13_H_14_N_4_OS (MW 274.32) C 56.91%, H 5.14%, N 20.49%, S, 11.69%. UV-visible (DMSO; λ_max_, nm): 265, 354, 408 (л→л*, n→л*). IR (KBr, cm^−1^) 3374, 3167 (s) ν(NH); 1651 (s) ν(C=O); 1556, 1602 (s) ν(C=N) + ν(C=C); 849 (m) ν(C=S). ^1^H NMR (DMSO-d_6_, 600 MHz; atoms labeling according to X-Ray structure of compound **2**) δ 11.71 (1H, s, N(3)-H), 11.95 (1H, s, N(1)-H), 8.60 (1H, s, N(4)-H), 8.29 (1H, s, C(1)-H), 8.63 (1H, s, C(6)-H), 7.22 (1H,d, J = 8.2 Hz, C(8)-H), 7.37 (1H,d, J = 8.2 Hz, C(7)-H), 7.40 (1H, s, C(5)-H), 2.37 (3H, s, C(11)-H), 3.07 (3H, s, C(13)-H). ^13^C{^1^H} NMR (DMSO-d_6_, 600 MHz) δ 136.9(C-1), 125.8 (C-2), 161.4 (C-10), 134.9 (C-3), 137.4 (C-4), 134.9 (C-9), 115.6 (C-8), 132.8 (C-7), 131.8 (C-6), 128.3 (C-5), 20.9 (C-11), 178.1 (C-12), 31.3 (C-13). ESI(H_2_L2 = C_13_H_14_N_4_OS) m/z 275 [H_3_L2]^+^ (60), 297 [H_2_L2Na]^+^ (100), 244 [H_3_L2–NH_2_Me]^+^ (50), 241 [H_3_L2-H_2_S]^+^ (10), 571 [2H_2_L2Na]^+^ (40), 1119 [4H_2_L2Na]^+^ (50), 1393 [5H_2_L2Na]^+^ (65), 1667 [6H_2_L2Na]^+^ (80), 1941 [7H_2_L2Na]^+^ (65), 705 [C_40_H_49_N_8_O_4_]^+^ (30), 979 [C_40_H_49_N_8_O_4_+H_2_L2]^+^ (20), 1253 [C_40_H_49_N_8_O_4_+2H_2_L2]^+^ (20), 1527 [C_40_H_49_N_8_O_4_+3H_2_L2]^+^ (30), 1801 [C_40_H_49_N_8_O_4_+4H_2_L2]^+^ (30).

#### 5.2.3. Synthesis of 6-Methyl-2-oxo-1,2-dihydro-quinoline-3-carbaldehyde-4N-ethylthiosemicarbazone (H_2_L3)

It was prepared using the same procedure as described for H_2_L1 with 4-ethyl-3-thiosemicarbazide (1.20 g, 0.01 mol) and 6-methyl-2-oxo-1,2-dihydroquinoline-3-carbaldehyde (1.87 g, 0.01 mol). A yellow product was obtained. Yield 2.45 g (85%). MP: 265–267 °C. Elemental Analysis Anal. C 58.25%, H 5.56%, N 19.32%, S 11.06%. Calcd. for C_14_H_16_N_4_OS (MW 288.37) C 58.31%, H 5.59%, N 19.43%, S 11.12%. UV-visible (DMSO; λ_max_, nm): 265, 354, 410 (л→л*, n→л*). IR (KBr, cm^−1^) 3374, 3153 (s) ν(NH); 1648 (s) ν(C=O); 1540, 1603 (s) ν(C=N) + ν(C=C); 840 (m) ν(C=S). ^1^H NMR (DMSO-d_6_, 600 MHz) δ 11.64 (1H, s, N(2)-H), 11.95 (1H, s, N(3)-H), 8.60 (1H, s, N(4)-H), 8.29 (1H, s, C(1)-H), 8.62 (1H, s, C(6)-H), 7.24 (1H,d, J = 8.3 Hz, C(7)-H), 7.37 (1H,d, J = 8.3 Hz, C(8)-H), 7.48 (1H,s, C(10)-H), 2.36 (3H,s,C(11)-H), 3.64 (2H, q, J = 8.2 Hz, C(13)-H), 1.20 (3H,t, J = 8.2 Hz, C(14)-H). ^13^C{^1^H} NMR (DMSO-d_6_, 600 MHz) δ 137.1 (C-1), 125.8 (C-2), 161.3 (C-3), 137.4 (C-4), 119.5 (C-5), 135.0 (C-6), 115.6 (C-7), 132.7 (C-8), 131.8 (C-9), 128.3 (C-10), 20.9 (C-11), 177.2 (C-12), 38.7 (C-13), 15.0 (C-14). ^15^N NMR (DMSO-d_6_, 600 MHz) δ 317 (s, N(1)), 172 (d, J = 89 Hz, N(2)-H), 150 (d, J = 89 Hz, N(3)-H), 119 (t, J = 89 Hz, N(4)-H). ESI (H_2_L3 = C_14_H_16_N_4_OS) m/z 311 [H_2_L3Na]^+^ (100), 289 [H_3_L3]^+^ (10), 244 [H_3_L2–NH_2_Et]^+^ (5), 255 [H_3_L3–H_2_S]^+^ (15), 266 [H_2_L3Na–H_2_NEt]^+^ (10), 599 [2H_2_L3Na]^+^ (65), 887 [3H_2_L3Na]^+^ (35), 1175 [4H_2_L3Na]^+^ (50), 1463 [5H_2_L3Na]^+^ (70), 1750 [6H_2_L3Na]^+^ (55).

#### 5.2.4. Synthesis of the Complex 1 (H2L1Cu)

A warm methanol solution (20 mL) containing H_2_L1 (130 mg, 0.5 mmol) was added to a methanol solution (20 mL) of Cu(NO_3_)_2_.3H_2_O (121 mg, 0.5 mmol). The resulting greenish solution was refluxed for 30 min. The solution was reduced to a small volume (3 mL), and by addition of Et_2_O a green powder precipitated, which was filtered off, washed with Et_2_O and dried under vacuum. Yield 213 mg (85%). Mp. 275–277 °C (dec). Elemental Analysis Anal. C 28.57%; H 3.55%, N 16.98%, S 6.28%. Calcd. for [C_12_H_12_N_4_OSCu(H_2_O)_3_](NO_3_)_2_ (MW 501.89) C 28.71%, H 3.61%, N 16.74%, S 6.39%. UV-visible (DMSO; λ_max_, nm; ε, cm^−1^M^−1^): 386 (19,020) (LMCT). IR (KBr disks, cm^−1^): 3376, 3138(s) ν(NH); 3266 (s) ν(NH_2_); 1637(s) ν(C=O); 1605, 1549 (s) ν(C=N) + ν(C=C); 825 (m) ν(C=S); 525 (w) ν(Cu-O); 469 (w) ν(Cu-N). ESI m/z 322 [HL1Cu]^+^ (100), 353 [HL1Cu+MeOH]^+^ (20), 644 [(HL1Cu)_2_]^+^ (30), 966 [(HL1Cu)_3_]^+^ (25), 1288 [(HL1Cu)_4_]^+^ (35), 1610 [(HL1Cu)_5_]^+^ (20), 1932 [(HL1Cu)_6_]^+^ (10).

#### 5.2.5. Synthesis of the Complex 2 (H_2_L2Cu)

It was prepared by the same procedure as described for **1** using **H_2_L2** (137 mg, 0.5 mmol) and Cu(NO_3_)_2_.3H_2_O (121 mg, 0.5 mmol). Yield 219 mg (88%). Mp. 288–290 °C (dec). Elemental Analysis Anal. C 31.39%, H 3.5%, N 17.00%, S 6.43%. Calcd. for [C_13_H_14_N_4_OSCu(H_2_O)_2_](NO_3_)_2_ (MW 497.90) C 31.36%, H 3.64%, N 16.88%, S 6.44%. UV-visible (DMSO; λ_max_, nm; ε, cm^−1^M^−1^): 391 (22,530) (LMCT). IR (KBr disks, cm^−1^): 3239 ν(NH); 1635(s) ν(C=O); 1605, 1548 (s) ν(C=N) + ν(C=C); 824 (m) ν(C=S); 523 (w) ν(Cu-O); 463 (w) ν(Cu-N). ESI m/z 336 [HL2Cu]^+^ (100), 367 [HL2Cu+MeOH]^+^ (15), 672 [(HL2Cu)_2_]^+^ (75), 1008 [(HL2Cu)_3_]^+^ (55), 1344 [(HL2Cu)_4_]^+^ (55), 1680 [(HL2Cu)_5_]^+^ (30).

#### 5.2.6. Synthesis of the Complex 3 (H_2_L3Cu)

It was prepared by the same procedure as described for **1** using **H_2_L3** (144 mg, 0.5 mmol) and Cu(NO_3_)_2_.3H_2_O (121 mg, 0.5 mmol). Yield 210 mg (82%). Mp. 292–294 °C (dec). Elemental Analysis Anal. C 32.55%, H 3.82%, N 16.28%, S 6.19%. Calcd. for [C_14_H_16_N_4_OSCu(H_2_O)_2_](NO_3_)_2_ (MW 511.93) C 32.84%, H 3.94%, N 16.42%, S 6.26%. UV-visible (DMSO; λ_max_, nm; ε, cm^−1^M^−1^): 392 (19,000) (LMCT). IR (KBr disks, cm^−1^): 3435, 3210 (s) ν(NH); 1646(s) ν(C=O); 1608, 1547 (s) ν(C=N) + ν(C=C); 820 (m) ν(C=S); 525 (w) ν(Cu-O); 463 (w) ν(Cu-N). ESI m/z 350 [HL3Cu]^+^ (100), 381 [HL3Cu+MeOH]^+^ (15), 700 [(HL3Cu)_2_]^+^ (90), 1050 [(HL3Cu)_3_]^+^ (50), 1400 [(HL3Cu)_4_]^+^ (30), 1750 [(HL3Cu)_5_]^+^ (15).

### 5.3. X-ray Crystallography

Single-crystal X-ray diffraction data of the complexes **1**–**3** were collected on GADDS (Bruker, Madison, WI, USA) or on APEX II CCD (Bruker, Milan, Italy) diffractometers at 110(2) K. The extended lists of crystallographic, experimental and data reduction parameters, refinement results and geometrical parameters are given in the Supplementary Information (Tables S1–S17) and in the deposited cif-files. CCDC 1,987,299, CCDC 841,988 and CCDC 841,992 contain the supplementary crystallographic data for this paper. The data can be obtained free of charge from The Cambridge Crystallographic Data Centre via www.ccdc.cam.ac.uk/structures. The structures of the complexes were solved by direct methods and refined by full matrix least-squares (the function minimized being [w (F_o_^2^–(1/k) F_c_^2^)^2^]). The hydrogen atoms were either placed in calculated positions or found in Fourier difference maps (in particular those of the water ligands). All non-hydrogen atoms were refined using anisotropic displacement parameters, while hydrogens were refined using a riding model unless otherwise specified.

Crystal Data for **1**: C_24_H_36_Cu_2_N_12_O_20_S_2_ (M = 1003.85 g/mol): triclinic, space group P1 (no. 1), a = 8.5216(3) Å, b = 10.0064(4) Å, c = 12.0178(5) Å, α = 110.055(2)°, β = 110.055(2)°, γ = 90.040(2)°, V = 954.56(6) Å^3^, Z = 1, T = 110(2) K, μ(CuKα) = 3.270 mm^−1^, Dcalc = 1.746 g/cm^3^, 21,621 reflections measured (3.95° ≤ Θ ≤ 60.0°), 5333 unique (Rint = 0.0472) which were used in all calculations. The final R1 was 0.0268 (I > 2σ(I)), and wR2 was 0.0611 (all data).

Crystal Data for **2**: C_13_H_18_CuN_6_O_9_S (M = 497.93 g/mol): triclinic, space group P-1 (no. 2), a = 8.146(3) Å, b = 10.159(3) Å, c = 12.635(4) Å, α = 93.59(4)°, β = 92.39(4)°, γ = 112.15(4)°, V = 964.2(6) Å^3^, Z = 2, T = 110(2) K, μ(MoKα) = 1.303 mm^−1^, Dcalc = 1.715 g/cm^3^, 6853 reflections measured (1.62° ≤ Θ ≤ 25.25°), 3485 unique (Rint = 0.0395) which were used in all calculations. The final R1 was 0.0560 (I > 2σ(I)), and wR2 was 0.1588 (all data).

Crystal Data for **3**: C_29_H_40_Cu_2_N_12_O_17_S_2_ (M = 1019.33 g/mol): triclinic, space group P-1 (no. 2), a = 10.3073(5) Å, b = 13.3389(7) Å, c = 15.2916(8) Å, α = 109.017(4)°, β = 94.252(4)°, γ = 94.665(4)°, V = 1969.8(2) Å^3^, Z = 2, T = 110(2) K, μ(CuKα) = 3.123 mm-1, Dcalc = 1.720 g/cm^3^, 30,628 reflections measured (3.53° ≤ Θ ≤ 59.99°), 5585 unique (Rint = 0.0677) which were used in all calculations. The final R1 was 0.0484 (I > 2σ(I)), and wR2 was 0.1303 (all data).

### 5.4. Experiments with Human Cells

Cu(II) complexes were dissolved in DMSO just before the experiment, and a calculated amount of drug solution was added to the cell growth medium until a final solvent concentration of 0.5% was obtained, which had no detectable effects on the cell viability. Cisplatin and oxaliplatin were dissolved in 0.9% sodium chloride solution. MTT (3-(4,5-dimethylthiazol-2-yl)-2,5-diphenyltetrazolium bromide) and cisplatin were obtained from Sigma Chemical Co, St. Louis, MO, USA.

#### 5.4.1. Cell Cultures

Human lung (A549), colon (LoVo and HCT-15) and pancreatic (BxPC3, PSN-1) carcinoma cell lines, along with melanoma (A375) cells, were obtained from the American Type Culture Collection (ATCC, Rockville, MD, USA). Embryonic kidney HEK293 cells were obtained from the European Collection of Cell Cultures (ECACC, Salisbury, UK). A431 are human cervical carcinoma cells kindly provided by Prof. F. Zunino (Division of Experimental Oncology B, Istituto Nazionale dei Tumori, Milan, Italy). The 2008 cells and cisplatin-resistant variant, C13*, are human ovarian adenocarcinoma cell lines that were kindly provided by Prof. G. Marverti (Dept. of Biomedical Science of Modena University, Italy). The LoVo OXP cells were derived, using a standard protocol, by growing LoVo cells in increasing concentrations of oxaliplatin and following nine months of selection of resistant clones. Cell lines were maintained in the logarithmic phase at 37 °C in a 5% carbon dioxide atmosphere using the following culture media containing 10% fetal calf serum (Euroclone, Milan, Italy), antibiotics (50 units/mL penicillin and 50 μg/mL streptomycin) and 2 mM l-glutamine: i) RPMI-1640 medium (Euroclone, Milan, Italy) for BxPC3, PSN-1, 2008 and C13* cells; ii) F-12 HAM′S (Sigma Chemical Co., Darmstadt, Germany) for A549, LoVo and LoVo OXP cells; and iii) DMEM for A375 and HEK293 cells.

#### 5.4.2. Cytotoxic Activity

The growth inhibitory effect towards tumor cells was evaluated by means of the MTT assay. Briefly, (3–8) × 10^3^ cells/well, depending on the growth characteristics of the cell line, were seeded in 96-well microplates in growth medium (100 μL). After 24 h, the medium was removed and replaced with fresh medium containing the compound to be studied at the appropriate concentration. Triplicate cultures were established for each treatment. After 72 h, each well was treated with 10 μL of a 5 mg/mL MTT saline solution, and following 5 h of incubation 100 μL of a sodium dodecylsulfate (SDS) solution in 0.01 M HCl were added. After an overnight incubation, cell growth inhibition was detected by measuring the absorbance of each well at 570 nm using a Bio-Rad 680 microplate reader (Bio-Rad Laboratories, Milan, Italy). The mean absorbance for each drug dose was expressed as a percentage of the absorbance of the control untreated well and plotted vs. the drug concentration. Data were fitted to a dose–response curve and the IC_50_ values (the drug concentrations that reduce the mean absorbance values at 570 nm to 50% of those of untreated cells’ control wells) were calculated with the four-parameter logistic model (4-PL). The final value was the mean ± S.D. of at least three independent experiments performed in triplicate.

#### 5.4.3. Spheroid Cultures and Acid Phosphatase (APH) Assay

Spheroid cultures were obtained by seeding 2.5 × 10^3^ cancer cells/well in a round bottom non-tissue culture treated 96-well plate (Greiner Bio-one, Kremsmünster, Austria) in phenol red free RPMI-1640 medium (Sigma Chemical Co., Darmstadt, Germany), containing 10% FCS and supplemented with 20% methyl cellulose stock solution. After 72 h, the medium was removed and replaced with fresh medium containing the compound to be studied at the appropriate concentration. A modified APH assay, which is based on the quantification of the cytosolic acid phosphatase activity, was used for determining the cell viability in spheroids [121]. Triplicate cultures were established for each treatment. After 72 h, each well was treated with 100 μL of the assay buffer (0.1 M sodium acetate, 0.1% Triton-X-100, supplemented with ImmunoPure p-nitrophenyl phosphate; Sigma Chemical Co., Darmstadt, Germany), and, following 3 h of incubation, 10 μL of 1 M NaOH solution were added. The inhibition of the cell growth induced by the tested complexes was detected by measuring the absorbance of each well at 405 nm, using a Bio-Rad 680 microplate reader. The mean absorbance for each drug dose was expressed as a percentage of the control untreated well absorbance (T/C) and plotted vs. the drug concentration. IC50 values (the drug concentrations that reduce the mean absorbance values at 405 nm to 50% of those in the untreated control wells) were calculated by the four-parameter logistic (4-PL) model. The evaluation was based on the means of at least four independent experiments.

#### 5.4.4. Cellular Accumulation of Cu

A375 cells (2 ×10^6^) were seeded in 75 cm^2^ flasks in growth medium (20 mL). After 24 h, the medium was replaced and the cells were incubated for 12 or 24 h in the presence of the tested complexes. Cell monolayers were washed twice with cold phosphate-buffered saline (PBS), harvested and counted. Samples were subjected to three freeze–thaw cycles at −80 °C and were then vigorously vortexed. The samples were treated with highly pure nitric acid (1 mL; Cu ≤ 0.005 mg kg^−1^, Trace-SELECT Ultra, Sigma Chemical Co., Darmstadt, Germany) and transferred into a microwave Teflon vessel. Subsequently, the samples were submitted to standard mineralization procedures. The samples were analyzed for platinum by using a Varian AA Duo graphite furnace atomic absorption spectrometer (Varian, Palo Alto, CA, USA) at the wavelength of 324 nm. The calibration curve was obtained using known concentrations of standard solutions purchased from Sigma Chemical Co. (Sigma Chemical Co., Darmstadt, Germany)

#### 5.4.5. DNA Fragmentation

A375 cells (5 ×∙10^6^) were seeded in 10 cm Petri dishes in 10 mL of culture medium. Subsequently, cells were treated with tested complexes for 24 h. Nuclear DNA fragmentation (mono- and oligo-nucleosome formation) was detected with the DNA damage ELISA kit (Enzo Life Sciences, Farmingdale, NY, USA) according to the manufacturer’s instructions.

#### 5.4.6. Reactive Oxygen Species (ROS) Production

The production of ROS was measured in A375 cells (10^4^ per well) grown for 24 h in a 96-well plate in RPMI medium without phenol red (Sigma Chemical Co., Darmstadt, Germany). Cells were then washed with PBS and loaded with 10 μM 5-(and-6)-chloromethyl-2′,7′-dichlorodihydrofluorescein diacetate acetyl ester (CM–H_2_DCFDA) (Molecular Probes-Invitrogen, Eugene, OR) for 25 min, in the dark. Afterwards, cells were washed with PBS and incubated with increasing concentrations of tested complexes. The fluorescence increase was estimated utilizing the wavelengths of 485 nm (excitation) and 527 nm (emission) in a Fluoroskan Ascent FL (Labsystem, Finland) plate reader. Antimycin (3 μM, Sigma Chemical Co., Darmstadt, Germany), a potent inhibitor of Complex III in the electron transport chain, was used as the positive control.

#### 5.4.7. Transmission Electron Microscopy (TEM) Analyses

About 10^6^ A375 cells were seeded in 24-well plates and, after 24 h incubation, treated with the tested compounds and incubated for additional 24 h. Cells were then washed with cold PBS, harvested and directly fixed with 1.5% glutaraldehyde buffer with 0.2 M sodium cacodylate, pH 7.4. After washing with buffer and after post-fixation with 1% OsO4 in 0.2 M cacodylate buffer, specimens were dehydrated and embedded in epoxy resin (Epon Araldite from Sigma Chemical Co., Darmstadt, Germany). Sagittal serial sections (1 μm) were counterstained with toluidine blue; thin sections (90 nm) were given contrast by staining with uranyl acetate and lead citrate. Micrographs were taken with a Hitachi H-600 electron microscope (Hitachi, Tokyo, Japan) operating at 75 kV. All photos were typeset in Corel Draw 11.

#### 5.4.8. Statistical Analysis

All the values are the means ± SD of no fewer than three measurements. Multiple comparisons were made by ANOVA followed by a Tukey–Kramer multiple comparison test (** *p* < 0.01; * *p* < 0.05).

## 6. Conclusions

In order to shed more light on the widely reported antimicrobial and antiproliferative activity of TSCs, the electronic and steric properties of the X, Z and Y moieties (Chart 1) have been previously investigated, also through computational analysis [122] based on Triapine as the model. In particular, some aspects appeared to be relevant for biological activity, such as: chelating ability, the presence of nitrogen atoms with hydrogen bond donor (*N*H) or acceptor (N-*N*=C) properties, the presence of a C=*S* moiety as a hydrogen bond donor, the presence of other hydrogen bond acceptor atoms (i.e., F, cfr. Fluorouracil), a suitable o/w partition coefficient to ensure the passive diffusion of the compound through the cell membrane [123], and additional hydrophobic moieties able to be involved in π-π interactions with biomolecules modifying the biological activity. The coordination of TSCs by a metal ion, in particular copper, can confer to the molecule specific chemical properties which in turn modulate intrinsic characteristics (such as cyclization and redox potential) as well as the biological fate (such as distribution, and target and off-target reactivity) [124,125].

Concerning the copper complexes considered in this paper, their biological activity is characterized by some peculiar properties:

1. the IC_50_ values against some human tumor cell lines derived from solid tumors are significantly lower (at nanomolar level) than those elicited by cisplatin; it is worth noting that a higher cytotoxicity, with respect to the reference compound, was retained in the 3D spheroids of colon cancer and melanoma cells, which is undoubtedly more predictive of in vivo results than standard 2D monolayer cultures;
2. the remarkable cancer cell-killing effect is consistent with their ability to accumulate in human melanoma cells, which may be favored by a suitable balance between the cationic nature of the overall complexes [126] and the hydrophobicity of the pro-ligand skeleton;
3. complexes **1**–**3** are markedly effective against human cancer cells with a developed resistance to cisplatin, with an RF factor of 1,8, 1.1 and 1.6 for **1**, **2** and **3**, respectively;
4. complexes **2** and **3** exhibit a significant preferential cytotoxicity against human melanoma cancer cells with respect to non-cancerous HEK293 cells;
5. Despite other copper thiosemicarbazone complexes having been shown to be primarily localized in mitochondria, thus compromising mitochondrial functions [127], TEM images of treated A375 cells indicated that mitochondria were quite conserved and that no alterations in internal structures were detected. Hence, the modest ROS formation induced by the tested complexes do not contribute in a significant manner to the DNA damage induced by the tested complexes. In addition, the morphological changes such as cell shrinkage and the formation of apoptotic bodies suggest the occurrence of the apoptotic process, likely due to the induction of DNA damage as revealed by DNA fragmentation. Further studies will be performed in order to better characterize the mechanism of cell death induced by the tested complexes.

In summary, a more promising in vitro antiproliferative activity for complexes **2** (bearing the -NHMe moiety) and **3** (bearing the -NHEt moiety) has been observed with respect to complex **1** (with the -NH_2_ group). It is worth noting that the presence of the -NHEt group within a thiosemicarbazone structure was reported to improve the biological activity, including anticancer, antifungal and antibacterial action, in other TSC Cu(II) [57,93,128,129,130] complexes and in Ru(II) TSC derivatives [131].

Overall, the results reported here can open interesting possibilities for the design of optimized metal-based thiosemicarbazone anticancer agents: further studies to better characterize the potential of this class of copper(II) complexes as anticancer agents are ongoing.

## Figures and Tables

**Table 1:** Parameters obtained from the simulations for the CW-EPR spectra of samples **1** and **3** in ethanol at 80 K. Hyperfine constants *A_xx_*, *A_yy_* and *A_zz_* are in cm^−1^.

*Assignment*	*g_zz_*	*A* *_zz_*	*g_xx_*	*g_yy_*	*A* *_xx_*	*A_yy_*
***1a***	2.388	137 × 10^−4^	2.07	2.08	3.3 × 10^−4^	3.3 × 10^−4^
***1b***	2.260	173 × 10^−4^	2.07	2.10	5.0 × 10^−4^	5.0 × 10^−4^
***3a***	2.385	147 × 10^−4^	2.01	2.08	4.0 × 10^−4^	4.0 × 10^−4^
***3b***	2.350	182 × 10^−4^	2.07	2.10	5.0 × 10^−4^	5.0 × 10^−4^

**Table 2:** Cyclic voltammetry data ^1^ for the thiosemicarbazone pro-ligands and their Cu(II) complexes.

Compound	Cathodic Peak Potential		Anodic Peak Potential ^3^	
	L^red^	I^red 2^	A	I^ox^
H_2_L1 H_2_L2 H_2_L3	−1.56			
**1**	−1.59	−0.26	−0.05	0.36
**2**	−1.56	−0.32	−0.05	0.27
**3**	−1.56	−0.31	−0.07	0.24

^1^ Potential values (in Volt ± 0.02) relative to SCE, measured by CV at a Pt electrode in DMSO/0.2 M [NBu_4_][BF_4_] at 0.2 V·s^−1^. ^2^ Broad wave which split into two at low scan rates (see text). ^3^ Redox processes detected only upon reversing the potential scan direction at a low cathodic potential limit, or after the formation of wave I^red^ at sufficiently low scan rates.

**Table 3:** Selected Bond Lengths (Å) and Angles (deg) for **2** and **1.**

	2	1a *	1b *
Cu1–O1	1.948(3)	1.973(3)	1.946(3)
Cu1–S1	2.266(2)	2.289(2)	2.278(1)
Cu1–N2	1.977(3)	1.975(3)	1.969(3)
Cu1–Ow1	1.928(3)	1.961(2)	1.943(3)
Cu1–Ow2	2.226(4)	2.254(2)	
Cu1–ON1			2.695(4)
Cu1–ON2			2.525(4)
N2–Cu1–Ow2	96.6(1)	100.7(1)	88.5(3)
Ow1–Cu1–Ow2	90.9(2)	97.0(1))	82.2(2)
Ow1–Cu1–ON1			113.2(2)
N2–Cu1–Ow1	172.4(1)	169.2(1)	170.6(1)
ON1–Cu1–ON2			158.1(2)

* **1a** and **1b** refer to the two independent molecules in the unit cell of compound **1**.

**Table 4:** Selected bond lengths (Å) and angles (deg) for **3.**

	3a(Cu1) *	3b(Cu2) *
Cu–O1	1.930(3)	1.929(3)
Cu–S1	2.263(1)	2.258(1)
Cu–N2	1.986(3)	1.977(3)
Cu–Om1	1.989(3)	
Cu–Ow1		1.931(3)
Cu–ON1	2.318(3)	2.388(3)
N2–Cu–O1	90.8(1)	91.3(1)
N2–Cu–S1	87.0(1)	87.4(1)
N2–Cu–ON1	122.3(1)	86.4(1)
ON1–Cu–Om1	80.1(1)	
ON1–Cu–Ow1		91.7(1)
S1–Cu–ON1	90.86(8)	99.93(8)
N2–Cu–Om1	157.6(1)	
N2–Cu–Ow1		178.0(1)
S1–Cu–O1	177.16(9)	168.12(9)

* **3a** and **3b** refer to the two independent molecules in the unit cell of compound **3**, shown in Figure 8 and Figure 9, respectively.

**Table 5:** Cytotoxicity studies.

IC_50_(µM) ± S.D.					
Compounds	HCT-15	A549	BxPC3	PSN-1	A375
**1**	8.4 ± 1.7	6.8 ± 1.3	7.8 ± 0.5	9.2 ± 1.6	0.6 ± 0.2
**H_2_L1**	>25	>50	16.8 ± 2.6	18.3 ± 2.8	17.5 ± 3.2
**2**	2.6 ± 1.2	5.6 ± 0.5	0.1 ± 0.08	4.6 ± 2.0	0.027 ± 0.005
**H_2_L2**	>25	>50	>25	>100	>25
**3**	1.7 ± 1.5	3.2 ± 0.6	0.3 ± 0.2	5.3 ± 0.3	0.031 ± 0.001
**H_2_L3**	23.4 ± 1.0	>25	>25	>100	>100
**cisplatin**	15.5 ± 3.8	6.9 ± 2.1	13.9 ± 5.9	13.5 ± 4.1	1.3 ± 0.6

The IC_50_ values were calculated by the four parameter logistic model (*p* < 0.05). Cells (3–8·10^3^·mL^−1^) were treated for 72 h with increasing concentrations of tested compounds dissolved in DMSO (Cytotoxicity was assessed by MTT test). S.D. = standard deviation.

**Table 6:** Cross-resistance profiles.

IC_50_(µM) ± S.D.						
Compounds	2008	C13*	R.F.	LoVo	LoVo-OXP	R.F.
**1**	9.7 ± 3.3	6.7 ± 1.2	0.7	0.7 ± 0.07	1.2 ± 0.6	1.8
**2**	11.2 ± 2.0	8.5 ± 3.5	0.8	1.9 ± 0.6	2.0 ± 0.04	1.1
**3**	2.8 ± 0.9	0.9 ± 0.7	0.3	0.7 ± 0.5	1.1 ± 0.1	1.6
**cisplatin**	2.1 ± 0.6	26.9 ± 4.3	12.8			
**oxaliplatin**				3.1 ± 0.3	53.4 ± 0.8	17.2

The IC_50_ values were calculated by the four parameter logistic model (*p* < 0.05). Cells (3–8·10^3^·mL^−1^) were treated for 72 h with increasing concentrations of tested compounds dissolved in DMSO. Cytotoxicity was assessed by MTT test. R.F. = IC_50_ (resistant subline)/IC_50_ (wild-type cells). S.D. = standard deviation.

**Table 7:** Cytotoxicity against non-cancerous cells.

Compounds	HEK293 IC_50_(µM) ± S.D.	SI A375
**1**	3.9 ± 3.1	6.5
**2**	1.3 ± 0.8	41.9
**3**	1.1 ± 0.2	47.8
**cisplatin**	21.6 ± 3.5	16.6

The IC_50_ values were calculated by a four parameter logistic model (*p* < 0.05). Cells (5·10^3^·mL^−1^) were treated for 72 h with increasing concentrations of tested compounds dissolved in DMSO. Cytotoxicity was assessed by MTT test. SI _A375_ = IC_50_ HEK293 non-cancer cells/IC_50_ A375 cancer cells. S.D. = standard deviation.

**Table 8:** Cytotoxicity towards human cancer cell spheroids.

IC_50_(µM) ± S.D.		
Compounds	HCT-15	A375
**1**	14.9 ± 2.3	18.6 ± 3.9
**2**	6.50 ± 0.80	7.6 ± 1.7
**3**	16.0 ± 2.8	11.3 ± 2.5
**cisplatin**	54.75 ± 1.26	32.8 ± 6.1

Spheroids (3∙10^3^ cells/well) were treated for 72 h with increasing concentrations of tested compounds. The growth inhibitory effect was evaluated by means of the APH assay. IC_50_ values were calculated from the dose-survival curves by the four-parameter logistic model (*p* < 0.05). S.D. = standard deviation.

## Supplementary Materials

The Supplementary Materials are available online. Figure S1: Cyclic voltammograms for H_2_L1 (top) and H_2_L3 (bottom). Figure S2: Cyclic voltammograms for **1** (top; C = 3.06 × 10^−3^ M) and for **3** (bottom; C = 3.02 × 10^−3^ M). Figure S3: ORTEP view of compound **2** with the full numbering scheme. Figure S4: Packing view of **2** showing the π-π stacking between the aromatic part of the ligands. Figure S5: View of the molecular packing in **2** along the *a* axis. Figure S6: Selected short intermolecular hydrogen bond interactions in **2**. Table S1: Crystal data and structure refinement for **2**. Table S2: Atomic coordinates (×10^4^) and equivalent isotropic displacement parameters (Å^2^ × 10^3^) for **2**. Table S3: Anisotropic displacement parameters (Å^2^ × 10^3^) for **2**. Table S4: Hydrogen coordinates (×10^4^) and isotropic displacement parameters (Å^2^ × 10^3^) for **2**. Table S5: Bond lengths [Å] and angles [°] for **2**. Table S6: Torsion angles [°] for **2**. Table S7: Least squares planes for **2**. Figure S7: ORTEP view of compound **1** showing the two independent molecules in the unit cell (**1a** (Cu2) and **1b** (Cu1)) with the numbering scheme. Figure S8: Molecular Packing of **1**. Figure S9: Molecular Packing of **1** down the *b* axis. Figure S10: Selected short H-bonding interactions in **1**. Figure S11: A view of the short H-bonding interactions involving the NH_2_ groups (N4 and N8, respectively). Table S8: Crystal data and structure refinement for **1**. Table S9: Atomic coordinates (×10^4^) and equivalent isotropic displacement parameters (Å^2^ × 10^3^) for **1**. Table S10: Bond lengths [Å] and angles [°] for **1**. Table S11: Anisotropic displacement parameters (Å^2^ × 10^3^) for **1**. Table S12: Hydrogen coordinates (×10^4^) and isotropic displacement parameters (Å^2^ × 10^3^) for **1**. Figure S12: ORTEPs with the numbering schemes of **3a**, **3b**, nitrate counter ions and clathrated water molecule. Figure S13: ORTEP view of the unit cell of **3**. Figure S14: Molecular packing of **3**. Figure S15: Molecular packing of **3**. Figure S16: Selected short H-bonding interactions in **3**. Table S13 Crystal data and structure refinement for **3**. Table S14: Atomic coordinates (×10^4^) and equivalent isotropic displacement parameters (Å^2^ × 10^3^) for **3**. Table S15: Bond lengths [Å] and angles [°] for **3**. Table S16: Anisotropic displacement parameters (Å^2^ × 10^3^) for **3**. Table S17: Hydrogen coordinates (×10^4^) and isotropic displacement parameters (Å^2^ × 10^3^) for **3**. Table S18: Cytotoxicity in A375 cancer cells. Figure S17 Dose-response curves for cell viability assessed in A375 cells.
