# Supplementary material for: Synthesis, Characterization and Biological Activity of Novel Cu(II) Complexes of 6-Methyl-2-Oxo-1,2-Dihydroquinoline-3-Carbaldehyde-4n-Substituted Thiosemicarbazones
Source: Molecules. 2020 Apr 17;25(8):1868. doi: 10.3390/molecules25081868 (PMC7221752; doi:10.3390/molecules25081868)
Supplement: Supplementary file 1 [file molecules-25-01868-s001.pdf]

Supplementary Material

Synthesis, Characterization and Biological Activity of Novel Cu(II) Complexes of 6-Methyl-2-Oxo-1,2-Dihydroquinoline-3-Carbaldehyde-4n-Substituted Thiosemicarbazones

E. Ramachandran, V. Gandin, R. Bertani, P. Sgarbossa, K. Natarajan, N. S. P. Bhuvanesh, A. Venzo, A. Zoleo, M. Mozzon, A. Dolmella, A. Albinati, C. Castellano, N. R. Conceição, M. F. C. Guedes da Silva and C. Marzano

List of Figures and Tables

Figure S1 Cyclic voltammograms for H_2L1 (top) and H_2L3 (bottom).

Figure S2 Cyclic voltammograms for **1** (top; C = 3.06 × 10^{-3} M) and for **3** (bottom; C = 3.02 × 10^{-3} M).

Figure S3 ORTEP view of compound **2** with the full numbering scheme.

Figure S4 Packing view of **2** showing the π-π stacking between the aromatic part of the ligands.

Figure S5 View of the molecular packing in **2** along the *a* axis

Figure S6 Selected short intermolecular hydrogen bond interactions in **2**.

Table S1 Crystal data and structure refinement for **2**

Table S2 Atomic coordinates (×10^4) and equivalent isotropic displacement parameters (Å^2 × 10^3) for **2.**

Table S3 Anisotropic displacement parameters (Å^2 × 10^3) for **2**.

Table S4 Hydrogen coordinates (×10^4) and isotropic displacement parameters (Å^2 × 10^3) for **2.**

Table S5 Bond lengths [Å] and angles [°] for **2.**

Table S6 Torsion angles [°] for **2.**

Table S7 Least Squares Planes for **2**.

Figure S7 ORTEP view of compound **1** showing the two independent molecules in the unit cell (**1a** (Cu2) and **1b** (Cu1)) with the numbering scheme.

Figure S8 Molecular Packing of **1.**

Figure S9 Molecular Packing of **1** down the *b* axis.

Figure S10 Selected short H-bonding interactions in **1**.

Figure S11 A view of the short H-bonding interactions involving the NH_2 groups (N4 and N8 respectively).

Table S8 Crystal data and structure refinement for **1.**

Table S9 Atomic coordinates (×10^4) and equivalent isotropic displacement parameters (Å^2 × 10^3) for **1.**

Table S10 Bond lengths [Å] and angles [°] for **1.**

Table S11 Anisotropic displacement parameters (Å^2 × 10^3) for **1.**

Table S12 Hydrogen coordinates (×10^4) and isotropic displacement parameters (Å^2 × 10^3) for **1**.

Figure S12 ORTEPs with the numbering schemes of **3a**, **3b**, nitrate counter ions and clathrated water molecule.

Figure S13 ORTEP view of the unit cell of **3**.

Figure S14 Molecular Packing of **3.**

Figure S15 Molecular Packing of **3**.

Figure S16 Selected short H-bonding interactions in **3**.

Table S13 Crystal data and structure refinement for **3.**

Table S14 Atomic coordinates (×10^4) and equivalent isotropic displacement parameters (Å^2 × 10^3) for **3**.

Table S15 Bond lengths [Å] and angles [°] for **3.**

Table S16 Anisotropic displacement parameters (Å^2 × 10^3) for **3**.

Table S17 Hydrogen coordinates (×10^4) and isotropic displacement parameters (Å^2 × 10^3) for **3.**

Table S18 Cytotoxicity in A375 cancer cells.

Figure S17 Dose-response curves for cell viability assessed in A375 cells.

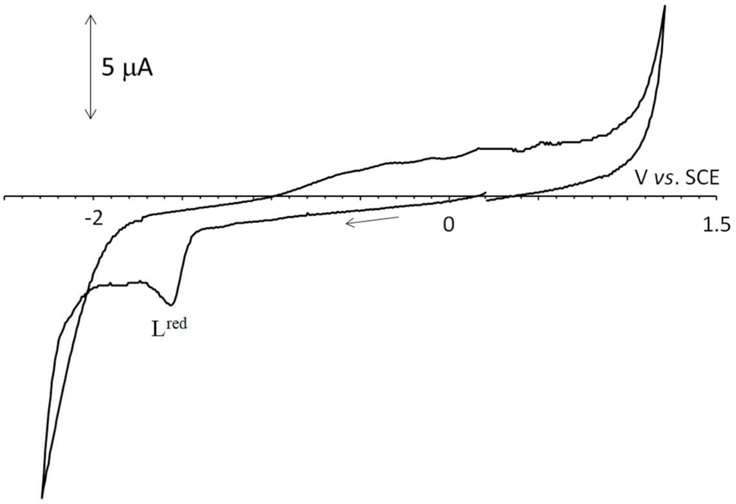

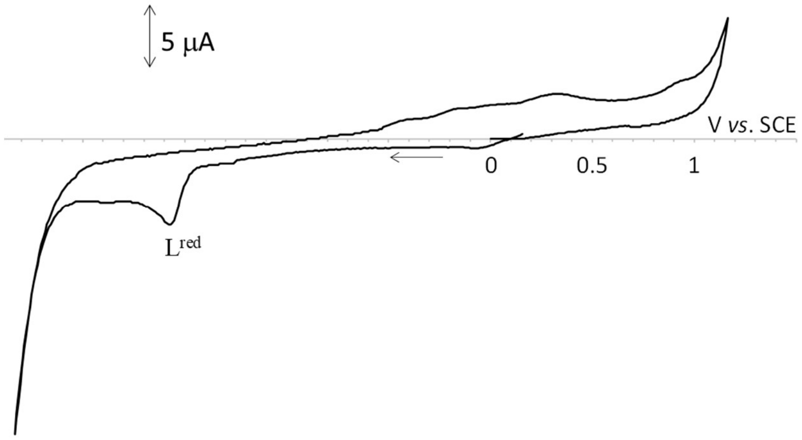

Figure S1. Cyclic voltammograms for H_2L1 (top) and H_2L3 (bottom), both at a concentration of 2.00 × 10^{-3} M, in $[NBu_4][BF_4]$ 0,2 M in DMSO at a platinum disc working electrode (*d* = 0.5 mm) and at a scan rate of 0.2 V.s^{-1}. The arrows indicate the cathodic initial scan direction.

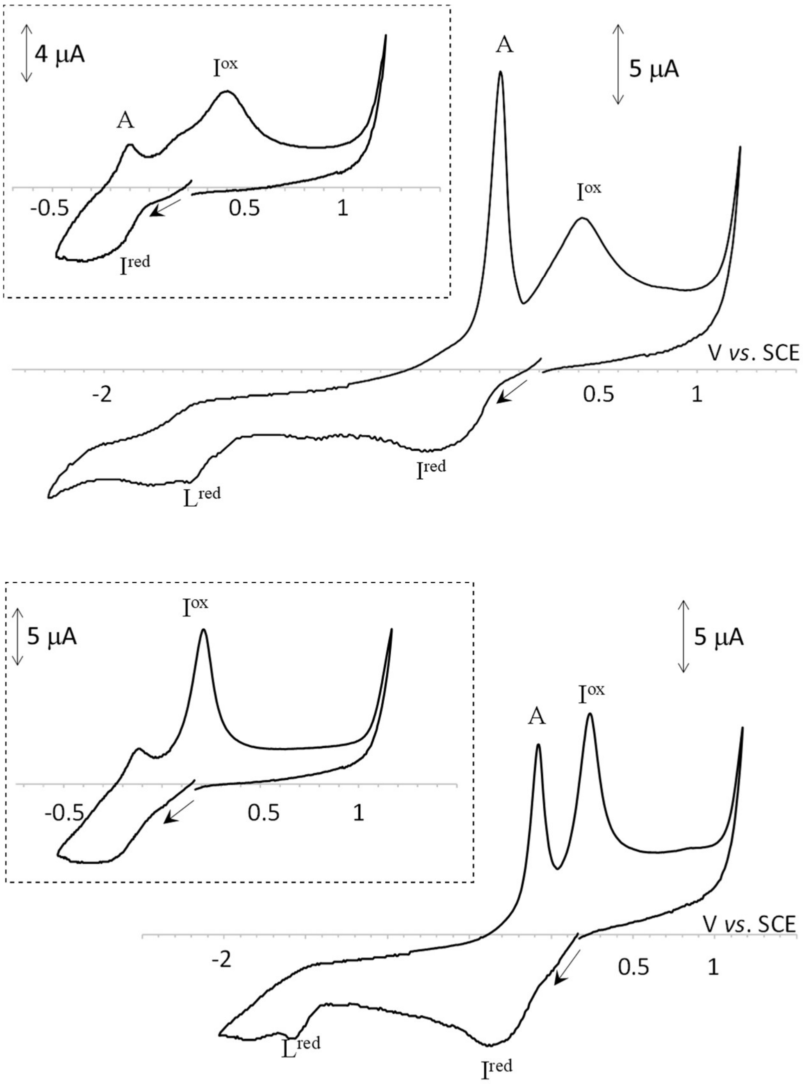

Figure S2. Cyclic voltammograms for **1** (top; C = 3.06 × 10^{-3} M) and for **3** (bottom; C = 3.02 × 10^{-3} M) in $[NBu_4][BF_4]$ 0,2 M in DMSO at a platinum disc working electrode (*d* = 0.5 mm) and at a scan rate of 0.2 V.s^{-1}. The arrows indicate the cathodic initial scan direction.

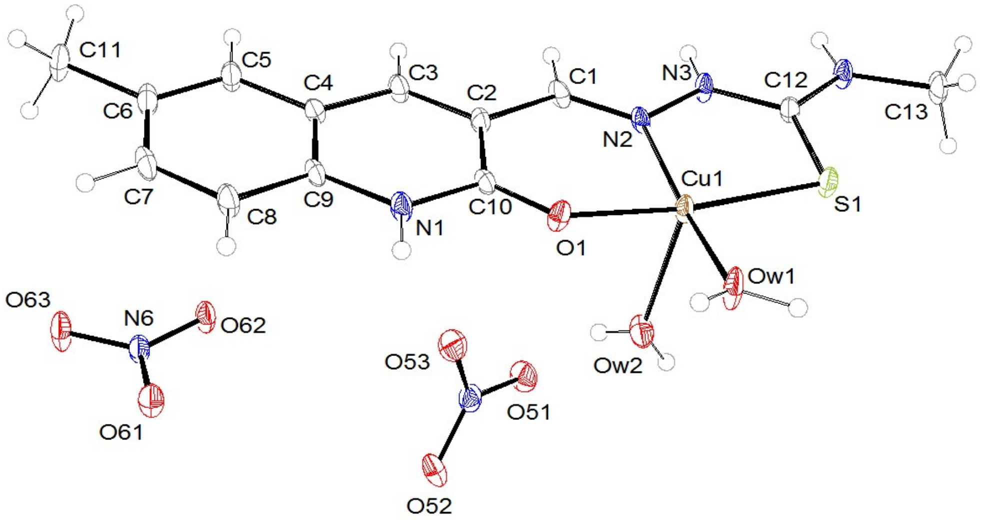

Figure S3. Ortep view of compound **2** with the full numbering scheme (Ellipsoids drawn at 50% probability).

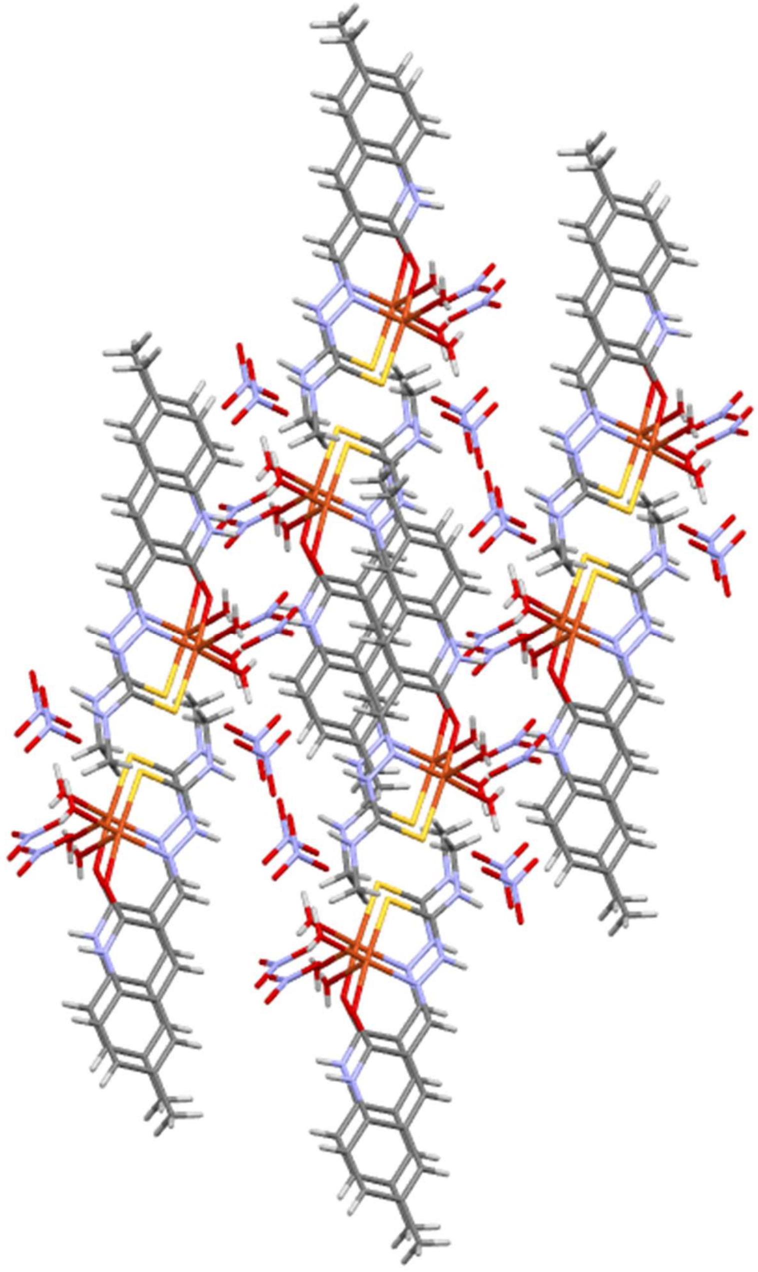

Figure S4. Packing view of **2** showing the π-π stacking between the aromatic part of the ligands.

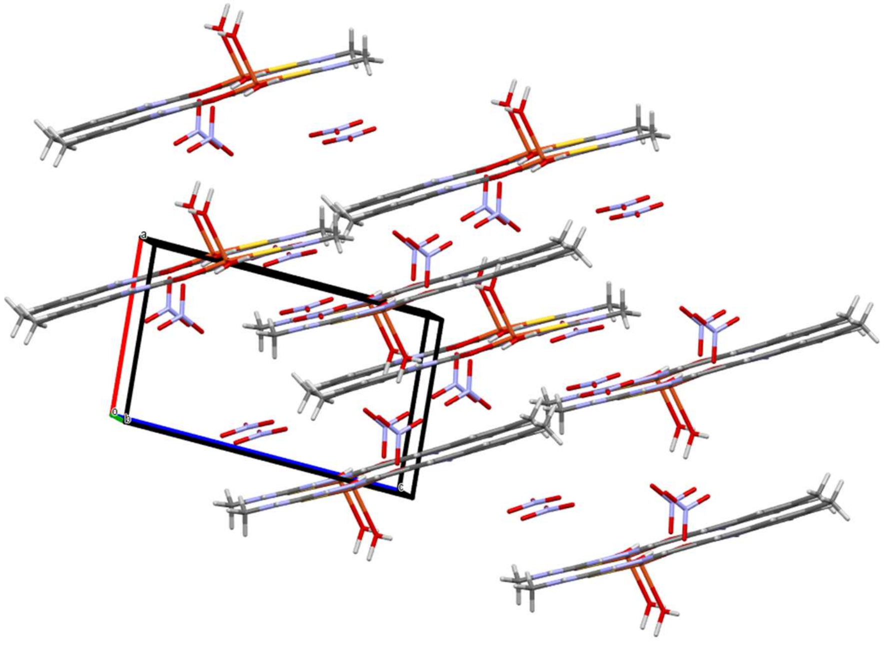

Figure S5. View of the molecular packing in **2** along the *a* axis.

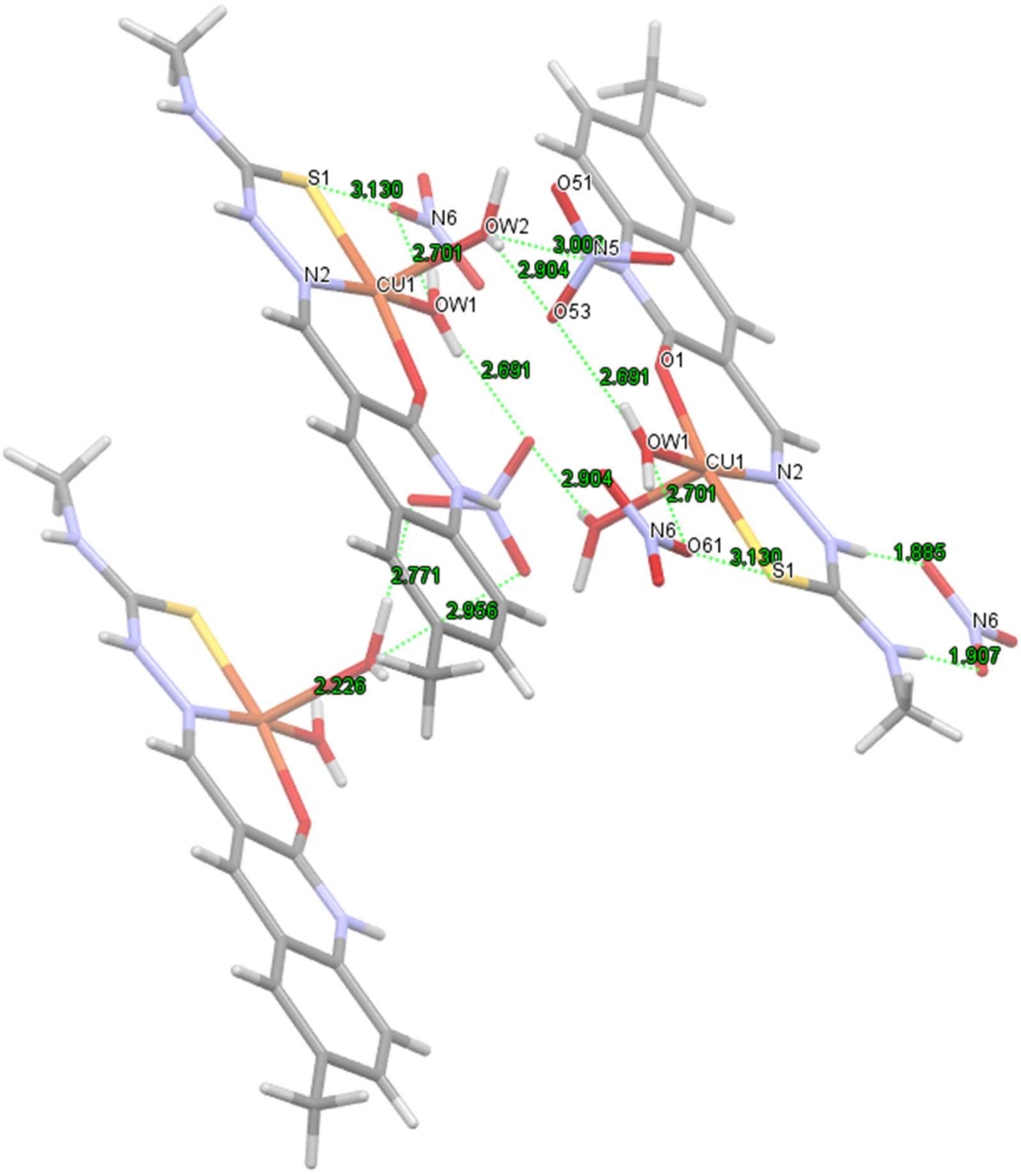

Figure S6. Selected short intermolecular hydrogen bond interactions in **2**.

A crystal of compound **2** was mounted on a Bruker APEXII diffractometer for the unit cell, space group determination and data collection and cooled using a cold nitrogen stream to 110(2)K.

Data were corrected for Lorentz and polarization factors with the data reduction software SAINT **[1]** and, empirically, for absorption using the SADABS program **[2]**

The cell constants were refined by least-squares, at the end of the data collection, using 5250 reflections ($\theta_{max} \leq 61.06$ °). The centrosymmetric choice for the space group (P−1) was confirmed by the successful refinement. The data were collected using ω scans, in steps of 0.5 °. For each of the 1440 collected frames counting time was 20 sec.

The structure was solved by direct methods **[3]** and refined by full matrix least-squares **[4]** (the function minimized being $\Sigma[w(F_o^2 - (1/k)\ F_c^2)^2]$).

The least-squares refinement was carried out using anisotropic displacement parameters for all non-hydrogen atoms. The contribution of hydrogen atoms, in their calculated positions, was included in the refinement using a riding model ($B(H) = a \times B_{(C_{bonded})}$ (Å^2), with a = 1.3 for the methyl groups and a = 1.2 for the remaining ones). The hydrogen atoms bonded to the coordinated water molecules were found from a Fourier Difference map and refined without constraints.

The scattering factors used, corrected for the real and imaginary parts of the anomalous dispersion, were taken from the literature. All calculations were carried out by using the WINGX **[5]**, SHELX**[3]** ORTEP **[6]** and MERCURY **[7]** programs.

More details of the data collection parameters, coordinates, ADPs and molecular geometry are listed in the deposited cif file.

References

1. APEX2 "Program for Data Collection and Integration on Area Detectors" BRUKER AXS Inc., 5465 East Cheryl Parkway, Madison, WI 53711–5373 USA.
2. G. M. Sheldrick, "SADABS (version 2008/1): Program for Absorption Correction for Data from Area Detector Frames", University of Gottingen, 2008.
3. G.M. Sheldrick, *Acta Cryst. A* (2008) 64, 112–122; *Acta Cryst. C* (2015) 71, 3–8.
4. M.C. Burla, R. Caliandro, B. Carrozzini, G. L. Cascarano, C. Cuocci, C. Giacovazzo, M.Mallamo, A. Mazzone, G. Polidori, *J. Appl. Cryst.* (2015). 48, 306–309; SIR 2019 vs.19.03.
5. L.J. Farrugia, *J. Appl. Cryst*. (2012) 45, 849–854; WinGX Version 2018.3.
6. L.J. Farrugia, *J. Appl. Cryst*. (1997) 30, 565; ORTEP for Windows vs. 2.0
7. Mercury 4.2.0, CCDC, Cambridge (2019)

Table S1. Crystal data and structure refinement for **2.**

Empirical formula	$C_{13}H_{18}CuN_6O_9S$	
Formula weight	497.93	
Temperature	110(2) K	
Wavelength	0.71073 Å	
Crystal system	Triclinic	
Space group	P − 1	
Unit cell dimensions	a = 8.146(3) Å	α = 93.59(4)°
	b = 10.159(3) Å	β = 92.39(4)°
	c = 12.635(4) Å	γ = 112.15(4)°
Volume	964.2(6) Å3	
Z	2	
Density (calculated)	1.715 Mg/m3	
Absorption coefficient	1.303 mm^{-1}	
F(000)	510	
Theta range for data collection	1.619 - 25.249°	
Index ranges	−9 <= h <= 9, −12 <= k <= 12, −15 <= l <= 15	
Reflections collected	6853	
Independent reflections	3485 [$R_{(int)}$ = 0.0395]	
Completeness to theta = 25.242°	99.60%	
Refinement method	Full-matrix least-squares on F2	
Data / restraints / parameters	3485 / 0 / 287	
Goodness-of-fit on F2	1.055	
Final R indices [I > 2σ(I)]	R1 = 0.0560, wR2 = 0.1434	
R indices (all data)	R1 = 0.0713, wR2 = 0.1588	
Extinction coefficient	n/a	

Table S2. Atomic coordinates (× 10^4) and equivalent isotropic displacement parameters ($Å^2$ × 10^3) for **2.** U(eq) is defined as one third of the trace of the orthogonalized U^{ij} tensor.

	x	y	z	U(eq)
Cu(1)	9806(1)	5898(1)	7698(1)	20(1)
S(1)	8405(1)	5445(1)	6050(1)	23(1)
OW1	9883(5)	4023(3)	7677(3)	33(1)
OW2	7559(5)	5261(4)	8750(3)	31(1)
O(1)	11324(4)	6400(3)	9012(2)	23(1)
O(51)	5670(4)	6615(3)	10307(3)	32(1)
O(52)	6065(4)	5746(4)	11774(2)	36(1)
O(53)	8304(4)	6793(3)	10845(3)	32(1)
O(61)	11510(4)	7615(3)	14157(2)	31(1)
O(62)	10619(4)	9073(3)	13359(2)	27(1)
O(63)	12082(5)	9712(3)	14910(3)	36(1)
N(1)	12711(5)	7581(4)	10540(3)	22(1)
N(2)	10030(4)	7897(3)	7631(3)	18(1)
N(3)	9251(5)	8180(4)	6741(3)	21(1)
N(4)	7811(5)	7517(4)	5115(3)	23(1)
N(5)	6670(5)	6386(4)	10984(3)	25(1)
N(6)	11408(5)	8796(4)	14144(3)	24(1)
C(1)	10799(5)	8947(4)	8340(3)	20(1)
C(2)	11714(5)	8860(4)	9325(3)	20(1)
C(3)	12448(5)	10060(5)	10016(3)	22(1)
C(4)	13373(5)	10062(5)	10990(3)	20(1)
C(5)	14200(6)	11295(5)	11705(3)	24(1)
C(6)	15094(6)	11246(5)	12645(3)	27(1)
C(7)	15146(6)	9926(5)	12881(3)	28(1)
C(8)	14380(6)	8708(5)	12210(3)	26(1)
C(9)	13485(5)	8772(4)	11249(3)	21(1)
C(10)	11892(5)	7547(4)	9582(3)	20(1)
C(11)	15979(6)	12566(5)	13403(4)	33(1)
C(12)	8480(5)	7137(4)	5957(3)	19(1)

Table S3. Anisotropic displacement parameters (Å^2 × 10^3) for **2**. The anisotropic displacement factor exponent takes the form: $-2\pi^2[h^2a \times {}^2U^{11} + ... + 2\ h\ k\ a \times b \times U^{12}]$.

	U11	U22	U33	U23	U13	U12
Cu(1)	31(1)	14(1)	14(1)	−1(1)	−8(1)	10(1)
S(1)	36(1)	15(1)	18(1)	−3(1)	−9(1)	10(1)
OW1	57(2)	21(2)	24(2)	−9(1)	−25(2)	22(2)
OW2	39(2)	33(2)	19(2)	−3(2)	−1(1)	12(2)
O(1)	31(2)	13(1)	23(2)	−1(1)	−12(1)	10(1)
O(51)	39(2)	32(2)	28(2)	6(1)	−9(1)	18(2)
O(52)	44(2)	45(2)	16(2)	4(1)	−3(1)	15(2)
O(53)	32(2)	34(2)	29(2)	4(1)	−5(1)	13(1)
O(61)	51(2)	17(2)	26(2)	−1(1)	−13(1)	16(1)
O(62)	43(2)	20(2)	20(2)	−2(1)	−14(1)	14(1)
O(63)	60(2)	26(2)	24(2)	−9(1)	−20(2)	22(2)
N(1)	31(2)	20(2)	16(2)	1(1)	−7(1)	12(2)
N(2)	26(2)	19(2)	11(2)	2(1)	−5(1)	11(1)
N(3)	37(2)	14(2)	14(2)	−1(1)	−11(1)	12(2)
N(4)	33(2)	19(2)	15(2)	−1(1)	−10(1)	11(2)
N(5)	34(2)	20(2)	21(2)	−3(2)	−2(2)	12(2)
N(6)	33(2)	19(2)	18(2)	−2(1)	−7(2)	9(2)
C(1)	32(2)	21(2)	13(2)	4(2)	−3(2)	17(2)
C(2)	23(2)	21(2)	13(2)	1(2)	−4(2)	7(2)
C(3)	27(2)	24(2)	15(2)	1(2)	−4(2)	12(2)
C(4)	22(2)	25(2)	13(2)	1(2)	0(2)	7(2)
C(5)	29(2)	25(2)	16(2)	−2(2)	−5(2)	10(2)
C(6)	28(2)	33(2)	16(2)	−6(2)	−5(2)	10(2)
C(7)	32(2)	38(3)	14(2)	1(2)	−7(2)	14(2)
C(8)	33(2)	29(2)	17(2)	1(2)	−7(2)	14(2)
C(9)	25(2)	25(2)	12(2)	2(2)	−3(2)	10(2)
C(10)	25(2)	22(2)	11(2)	3(2)	−2(2)	8(2)
C(11)	37(3)	33(3)	23(2)	−11(2)	−13(2)	10(2)
C(12)	24(2)	18(2)	15(2)	−2(2)	−1(2)	8(2)

Table S4. Hydrogen coordinates (x 10^4) and isotropic displacement parameters ($Å^2 \times 10^3$).

	x	y	z	U(eq)
HW11	9280(60)	3320(60)	7150(40)	28(13)
HW12	10500(70)	3870(60)	8090(40)	27(14)
HW21	6180(70)	5030(50)	8640(40)	28(12)
HW22	7600(70)	5700(60)	9210(50)	29(16)
H(1)	12756	6776	10729	26
H(3)	9253	9039	6681	26
H(4)	7895	8407	5122	27
H(1A)	10762	9846	8206	24
H(3A)	12333	10917	9836	26
H(5)	14138	12173	11533	29
H(7)	15737	9876	13533	34
H(8)	14451	7836	12391	31
H(11A)	16028	13410	13048	50
H(11B)	17187	12655	13620	50
H(11C)	15294	12490	14032	50
H(13A)	7651	6005	3966	45
H(13B)	6835	7121	3591	45
H(13C)	5757	5912	4334	45

Table S5. Bond lengths [Å] and angles [°] for **2.**

Cu(1)-OW1	1.928(3)
Cu(1)-O(1)	1.948(3)
Cu(1)-N(2)	1.977(3)
Cu(1)-OW2	2.226(4)
Cu(1)-S(1)	2.2663(15)
S(1)-C(12)	1.708(4)
OW1-HW11	0.92(6)
OW1-HW12	0.77(6)
OW2-HW21	1.06(5)
OW2-HW22	0.70(6)
O(1)-C(10)	1.245(5)
O(51)-N(5)	1.248(5)
O(52)-N(5)	1.242(5)
O(53)-N(5)	1.260(5)
O(61)-N(6)	1.235(5)
O(62)-N(6)	1.262(5)
O(63)-N(6)	1.250(5)
N(1)-C(10)	1.349(5)
N(1)-C(9)	1.380(6)
N(1)-H(1)	0.88
N(2)-C(1)	1.289(6)
N(2)-N(3)	1.366(5)
N(3)-C(12)	1.346(5)
N(3)-H(3)	0.88
N(4)-C(12)	1.319(5)
N(4)-C(13)	1.457(5)
N(4)-H(4)	0.88
C(1)-C(2)	1.448(5)
C(1)-H(1A)	0.95
C(2)-C(3)	1.372(6)
C(2)-C(10)	1.450(6)
C(3)-C(4)	1.415(6)
C(3)-H(3A)	0.95
C(4)-C(9)	1.405(6)
C(4)-C(5)	1.418(6)
C(5)-C(6)	1.380(6)
C(5)-H(5)	0.95
C(6)-C(7)	1.408(7)
C(6)-C(11)	1.513(6)
C(7)-C(8)	1.372(7)
C(7)-H(7)	0.95
C(8)-C(9)	1.408(6)
C(8)-H(8)	0.95
C(11)-H(11A)	0.98
C(11)-H(11B)	0.98
C(11)-H(11C)	0.98
C(13)-H(13A)	0.98
C(13)-H(13B)	0.98
C(13)-H(13C)	0.98
OW1-Cu(1)-O(1)	87.95(14)
OW1-Cu(1)-N(2)	172.41(15)
O(1)-Cu(1)-N(2)	91.19(13)
OW1-Cu(1)-OW2	90.86(16)
O(1)-Cu(1)-OW2	85.35(14)
N(2)-Cu(1)-OW2	96.58(14)
OW1-Cu(1)-S(1)	92.53(11)

O(1)-Cu(1)-S(1)	171.79(10)
N(2)-Cu(1)-S(1)	87.25(11)
OW2-Cu(1)-S(1)	102.83(11)
C(12)-S(1)-Cu(1)	95.24(15)
Cu(1)-OW1-HW11	122(3)
Cu(1)-OW1-HW12	120(4)
HW11-OW1-HW12	119(5)
Cu(1)-OW2-HW21	135(3)
Cu(1)-OW2-HW22	119(5)
HW21-OW2-HW22	89(5)
C(10)-O(1)-Cu(1)	128.7(3)
C(10)-N(1)-C(9)	125.6(4)
C(10)-N(1)-H(1)	117.2
C(9)-N(1)-H(1)	117.2
C(1)-N(2)-N(3)	116.9(3)
C(1)-N(2)-Cu(1)	127.1(3)
N(3)-N(2)-Cu(1)	115.9(3)
C(12)-N(3)-N(2)	119.6(3)
C(12)-N(3)-H(3)	120.2
N(2)-N(3)-H(3)	120.2
C(12)-N(4)-C(13)	124.3(4)
C(12)-N(4)-H(4)	117.8
C(13)-N(4)-H(4)	117.8
O(52)-N(5)-O(51)	120.8(4)
O(52)-N(5)-O(53)	120.3(4)
O(51)-N(5)-O(53)	118.9(4)
O(61)-N(6)-O(63)	119.8(3)
O(61)-N(6)-O(62)	119.9(3)
O(63)-N(6)-O(62)	120.3(3)
N(2)-C(1)-C(2)	124.9(4)
N(2)-C(1)-H(1A)	117.5
C(2)-C(1)-H(1A)	117.5
C(3)-C(2)-C(1)	118.7(4)
C(3)-C(2)-C(10)	119.7(4)
C(1)-C(2)-C(10)	121.6(4)
C(2)-C(3)-C(4)	122.0(4)
C(2)-C(3)-H(3A)	119
C(4)-C(3)-H(3A)	119
C(9)-C(4)-C(3)	117.8(4)
C(9)-C(4)-C(5)	118.8(4)
C(3)-C(4)-C(5)	123.4(4)
C(6)-C(5)-C(4)	121.3(4)
C(6)-C(5)-H(5)	119.3
C(4)-C(5)-H(5)	119.3
C(5)-C(6)-C(7)	118.0(4)
C(5)-C(6)-C(11)	121.1(4)
C(7)-C(6)-C(11)	120.9(4)
C(8)-C(7)-C(6)	122.7(4)
C(8)-C(7)-H(7)	118.6
C(6)-C(7)-H(7)	118.6
C(7)-C(8)-C(9)	118.9(4)
C(7)-C(8)-H(8)	120.6
C(9)-C(8)-H(8)	120.6
N(1)-C(9)-C(4)	118.6(4)
N(1)-C(9)-C(8)	121.1(4)
C(4)-C(9)-C(8)	120.3(4)
O(1)-C(10)-N(1)	117.9(4)
O(1)-C(10)-C(2)	125.9(4)

N(1)-C(10)-C(2)	116.1(4)
C(6)-C(11)-H(11A)	109.5
C(6)-C(11)-H(11B)	109.5
H(11A)-C(11)-H(11B)	109.5
C(6)-C(11)-H(11C)	109.5
H(11A)-C(11)-H(11C)	109.5
H(11B)-C(11)-H(11C)	109.5
N(4)-C(12)-N(3)	115.5(4)
N(4)-C(12)-S(1)	122.7(3)
N(3)-C(12)-S(1)	121.9(3)
N(4)-C(13)-H(13A)	109.5
N(4)-C(13)-H(13B)	109.5
H(13A)-C(13)-H(13B)	109.5
N(4)-C(13)-H(13C)	109.5
H(13A)-C(13)-H(13C)	109.5
H(13B)-C(13)-H(13C)	109.5

Table S6. Torsion angles [°] for **2.**

C(1)-N(2)-N(3)-C(12)	177.8(4)
Cu(1)-N(2)-N(3)-C(12)	−3.5(5)
N(3)-N(2)-C(1)-C(2)	−179.1(4)
Cu(1)-N(2)-C(1)-C(2)	2.3(6)
N(2)-C(1)-C(2)-C(3)	−178.9(4)
N(2)-C(1)-C(2)-C(10)	2.1(7)
C(1)-C(2)-C(3)-C(4)	−179.6(4)
C(10)-C(2)-C(3)-C(4)	−0.6(6)
C(2)-C(3)-C(4)-C(9)	−1.5(6)
C(2)-C(3)-C(4)-C(5)	177.7(4)
C(9)-C(4)-C(5)-C(6)	−0.5(6)
C(3)-C(4)-C(5)-C(6)	−179.7(4)
C(4)-C(5)-C(6)-C(7)	−0.7(6)
C(4)-C(5)-C(6)-C(11)	179.8(4)
C(5)-C(6)-C(7)-C(8)	1.3(7)
C(11)-C(6)-C(7)-C(8)	−179.2(4)
C(6)-C(7)-C(8)-C(9)	−0.7(7)
C(10)-N(1)-C(9)-C(4)	2.6(6)
C(10)-N(1)-C(9)-C(8)	−177.1(4)
C(3)-C(4)-C(9)-N(1)	0.7(6)
C(5)-C(4)-C(9)-N(1)	−178.6(4)
C(3)-C(4)-C(9)-C(8)	−179.6(4)
C(5)-C(4)-C(9)-C(8)	1.1(6)
C(7)-C(8)-C(9)-N(1)	179.2(4)
C(7)-C(8)-C(9)-C(4)	−0.5(7)
Cu(1)-O(1)-C(10)-N(1)	170.4(3)
Cu(1)-O(1)-C(10)-C(2)	−7.8(6)
C(9)-N(1)-C(10)-O(1)	177.0(4)
C(9)-N(1)-C(10)-C(2)	−4.6(6)
C(3)-C(2)-C(10)-O(1)	−178.2(4)
C(1)-C(2)-C(10)-O(1)	0.7(7)
C(3)-C(2)-C(10)-N(1)	3.5(6)
C(1)-C(2)-C(10)-N(1)	−177.5(4)
C(13)-N(4)-C(12)-N(3)	179.2(4)
C(13)-N(4)-C(12)-S(1)	−0.1(6)
N(2)-N(3)-C(12)-N(4)	−178.0(3)
N(2)-N(3)-C(12)-S(1)	1.3(6)
Cu(1)-S(1)-C(12)-N(4)	−179.7(3)
Cu(1)-S(1)-C(12)-N(3)	1.1(4)

Table S7. Squares Planes for **2**.

Least-squares planes (x,y,z in crystal coordinates) and deviations from them		
	(* indicates atom used to define plane)	
	7.0664 (0.0066) x − 0.7771 (0.0106) y − 5.9994 (0.0131) z = 1.8713 (0.0126)	
	*0.0156 (0.0015)	S1
	*−0.0076 (0.0024)	C12
	*−0.0143 (0.0026)	N3
	*0.0248 (0.0020)	N2
	*−0.0185 (0.0012)	Cu1
	−0.0048 (0.0051)	N4
	0.0198 (0.0066)	C13
	−2.1881 (0.0043)	OW2
	0.1943 (0.0046)	OW1
Rms deviation of fitted atoms = 0.0171		
6.7710 (0.0076) x − 0.5716 (0.0122) y − 6.7952 (0.0136) z = 1.1226 (0.0142)		
Angle to previous plane (with approximate esd) = 4.245 (0.160)		
* 0.0553 (0.0023)	O1	
* −0.0133 (0.0028)	C10	
* −0.0340 (0.0028)	C2	
* 0.0107 (0.0029)	C1	
* 0.0322 (0.0024)	N2	
* −0.0509 (0.0016)	Cu1	
−0.1112 (0.0053)	N1	
−0.0752 (0.0056)	C3	
−2.2509 (0.0042)	OW2	
0.1228 (0.0051)	OW1	
Rms deviation of fitted atoms = 0.0368		
6.9081 (0.0069) x − 0.6846 (0.0128) y − 6.4568 (0.0103) z = 1.4673 (0.0129)		
Angle to previous plane (with approximate esd) = 1.855 (0.162)		

* 0.0440 (0.0032)	C10
* −0.0027 (0.0033)	C2
* −0.0239 (0.0034)	C3
* −0.0143 (0.0036)	C4
* 0.0115 (0.0034)	C5
* 0.0254 (0.0035)	C6
* −0.0009 (0.0036)	C7
* −0.0136 (0.0035)	C8
* −0.0150 (0.0037)	C9
* −0.0106 (0.0031)	N1
0.0986 (0.0044)	O1
−0.0048 (0.0053)	C1
Rms deviation of fitted atoms = 0.0201	
6.8630 (0.0063) x − 0.5639 (0.0106) y − 6.5133 (0.0098) z = 1.4810 (0.0089)	
Angle to previous plane (with approximate esd) = 0.718 (0.140)	
* 0.0402 (0.0013)	S1
* −0.0974 (0.0012)	Cu1
* 0.0602 (0.0014)	O1
* −0.0062 (0.0024)	C1
* −0.0126 (0.0031)	N2
* 0.0159 (0.0024)	N3
0.0563 (0.0045)	C12
0.1241 (0.0047)	N4
−0.0149 (0.0049)	C2
0.0137 (0.0049)	C10
Rms deviation of fitted atoms = 0.0503	

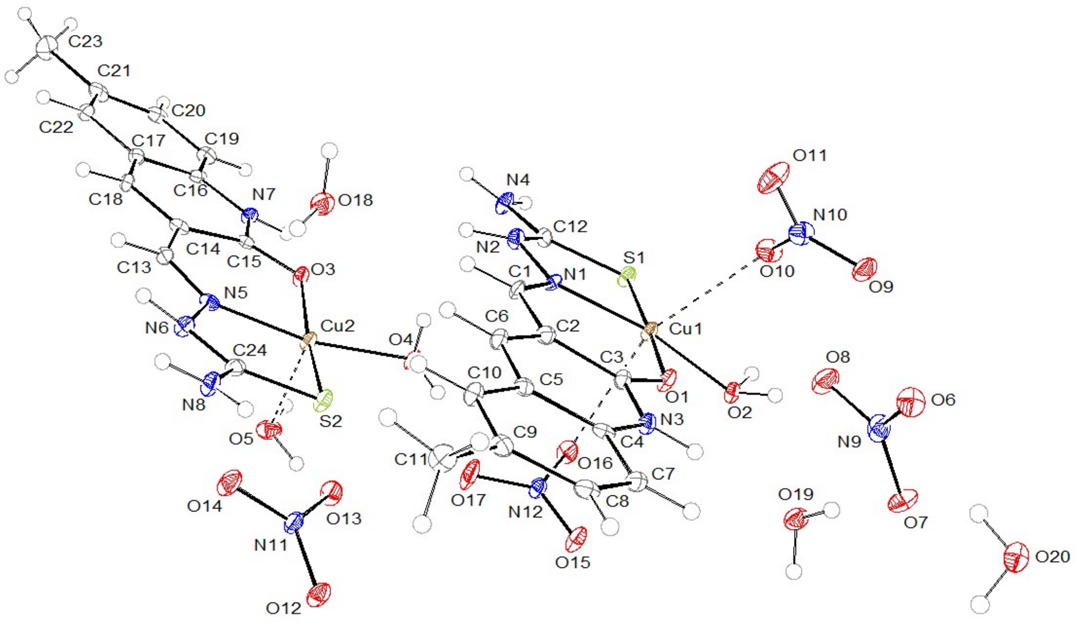

Figure S7. Ortep view of compound **1** showing the two independent molecules in the unit cell (**1a** (Cu2) and **1b** (Cu1)) with the numbering scheme. (Ellipsoids drawn at 50% probability).

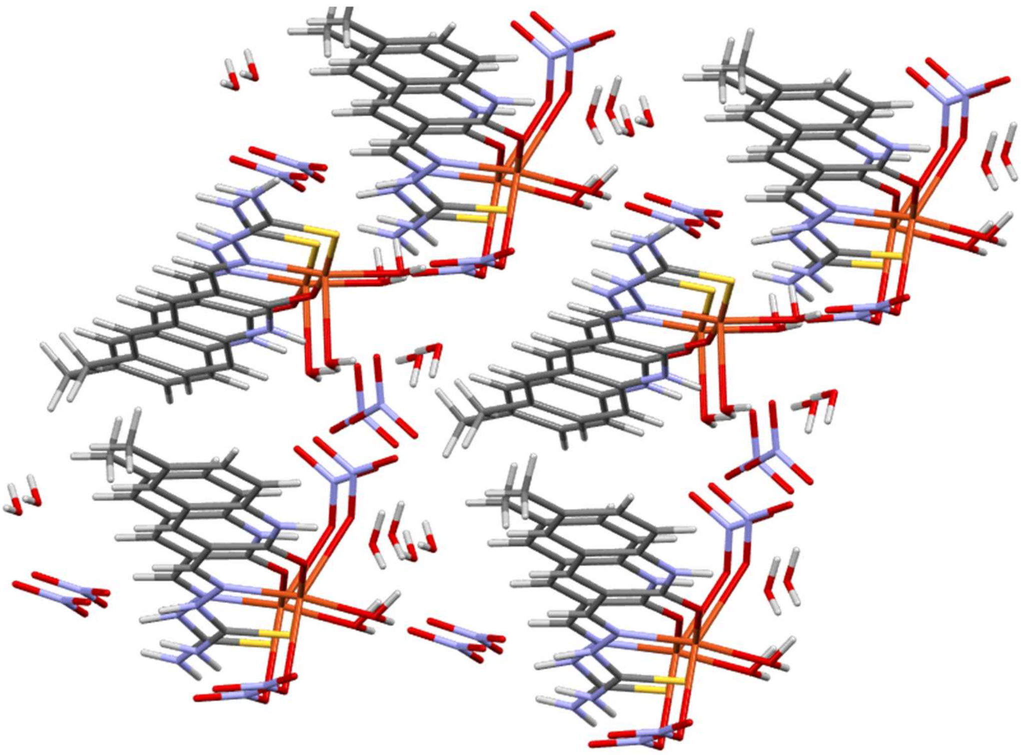

Figure S8. Molecular Packing of **1.**

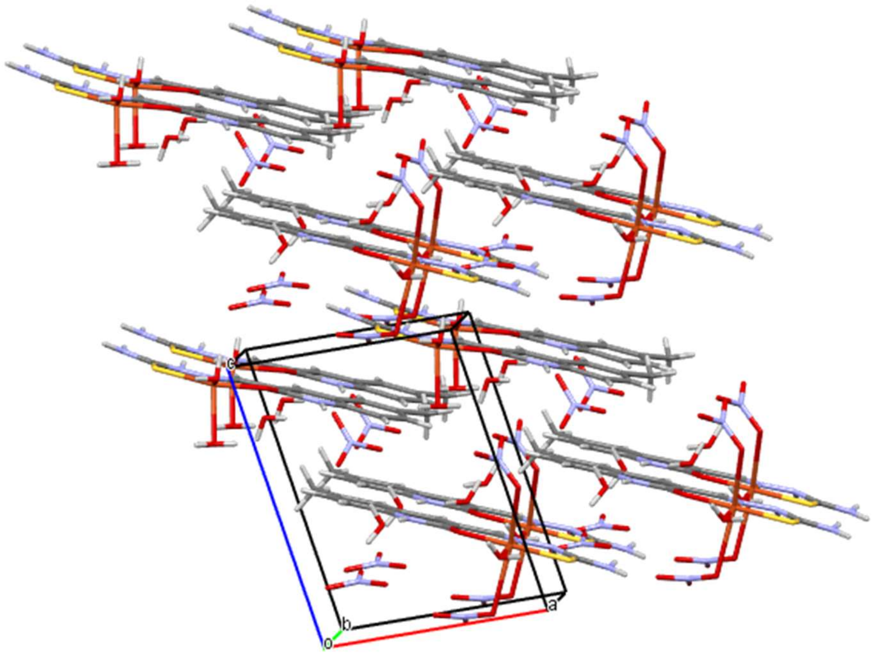

Figure S9. Molecular Packing of **1** down the *b* axis.

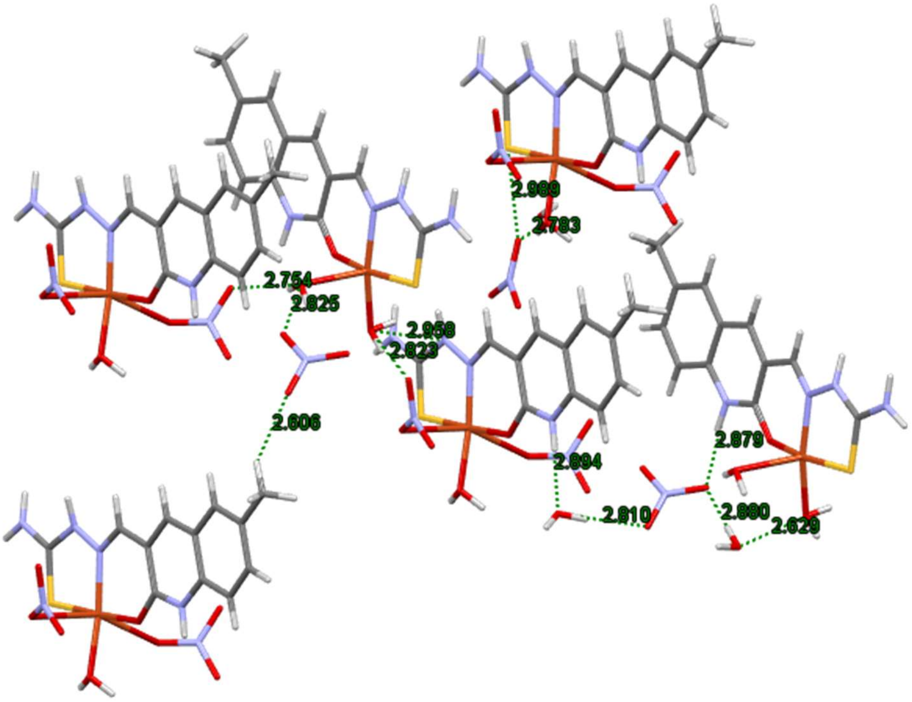

Figure S10. Selected short H-bonding interactions in **1**.

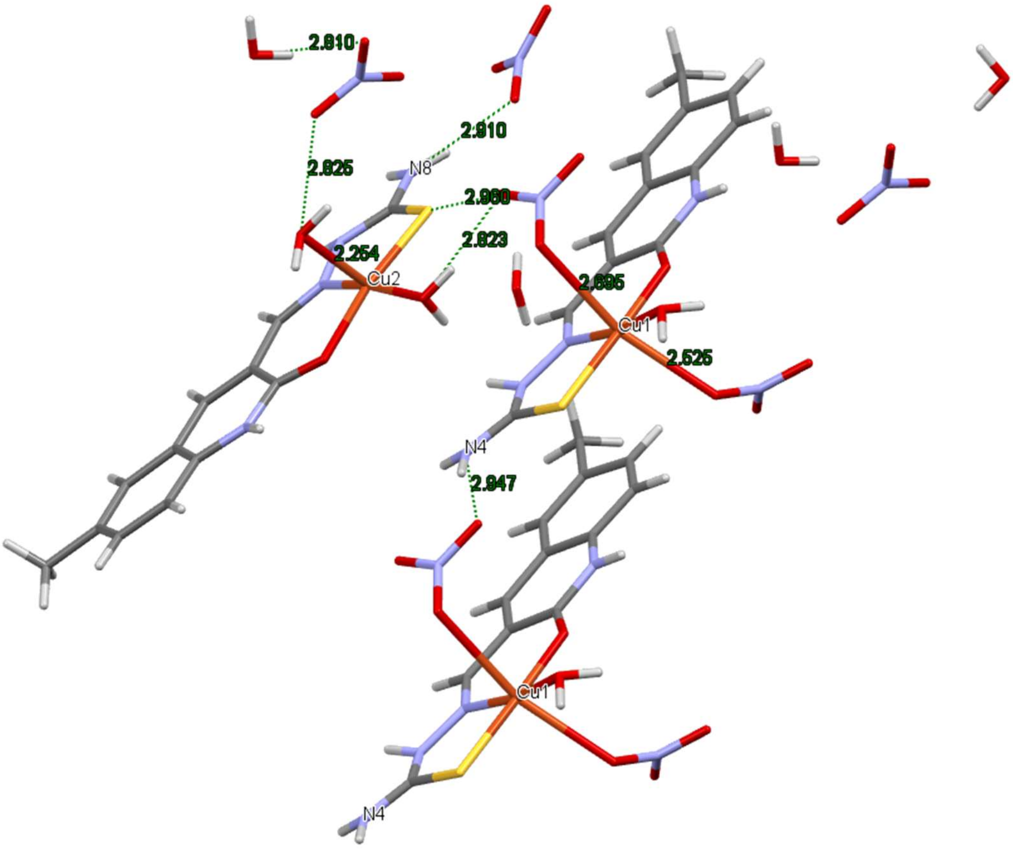

Figure S11. A view of the short H-bonding interactions involving the NH_2 groups (N4 and N8 respectively).

A suitable green crystal, with approximate dimensions 0.06 x 0.04 x 0.03 mm^3, was mounted on a nylon loop and placed in a cold nitrogen stream at 110(2) K, on a BRUKER GADDS X-ray diffractometer that was used for the unit cell determination and the data collection. The goniometer was controlled using the FRAMBO software, v.4.1.05.[1] The crystal to detector distance was set at 5.0 cm. The X-ray radiation employed was from a Cu X-ray tube (K_α = 1.5418 Å at 40 kV and 40 mA) and monochromated with a graphite monochromator (175 mm collimator with 0.5 mm pinholes).

For the final cell constants determination 180 data frames were collected with $\Delta\omega$ = 0.5 °. These reflections were used to determine the unit cell using the program Cell Now.[2]

Integrated intensities were obtained by using the APEX2 program.[3] Data were corrected for Lorentz and polarization factors, as well as for crystal decay. The SADABS [4] program was used for the absorption correction. Statistical tests indicated a non-centrosymmetric space group (*P*1); this choice was later confirmed by the successful refinement.

The structure was solved by direct methods **[5]** and refined by full matrix least-squares **[5]** (the function minimized being $\Sigma[w(F_o^2 - (1/k)\ F_c^2)^2]$).

While the hydrogen atoms bound to carbons were placed in idealized positions, those bound to the nitrogen and oxygen atoms were located from difference Fourier maps, and refined using a riding model. H4P and H18P could not be located from the difference Fourier maps and were modeled based on the hydrogen bonding acceptors positions. All non-hydrogen atoms were refined using Anisotropic Displacements Parameters. Program "X-seed" was employed for the final data presentation and structure plots.[6]

Three clathrated water molecules were also located from the Fourier difference maps.

References

1. FRAMBO v. 4.1.05 "Program for Data Collection on Area Detectors" BRUKER-Nonius Inc., 5465 East Cheryl Parkway, Madison, WI 53711–5373 USA
2. Sheldrick, G. M. "Cell_Now (version 2008/1): Program for Obtaining Unit Cell Constants from Single Crystal Data": University of Göttingen, Germany
3. APEX2 "Program for Data Collection and Integration on Area Detectors" BRUKER AXS Inc., 5465 East Cheryl Parkway, Madison, WI 53711–5373 USA
4. Sheldrick, G.M. "SADABS (version 2008/1): Program for Absorption Correction for Data from Area Detector Frames", University of Göttingen, 2008
5. Sheldrick, G.M. (2008).ActaCryst. A64, 112–122; as implemented in SHELXTL, BRUKER AXS Inc., 5465 East Cheryl Parkway, Madison, WI 53711–5373 USA
6. Barbour, L.J.,(2001) "X-Seed (version 2.0): A Software Tool for Supramolecular Crystallography" *J. Supramol. Chem.* **2001**, *1,* 189–191.

Table S8. Crystal data and structure refinement for **1.**

Empirical formula	$C_{24}H_{36}Cu_2N_{12}O_{20}S_2$	
Formula weight	1003.85	
Temperature	110(2) K	
Wavelength	1.54178 Å	
Crystal system	Triclinic	
Space group	P1	
Unit cell dimensions	a = 8.5216(3) Å	α = 110.055(2)°
	b = 10.0064(4) Å	β = 96.958(3)°
	c = 12.0178(5) Å	γ = 90.040(2)°
Volume	954.56(6) Å3	
Z	1	
Density (calculated)	1.746 Mg/m3	
Absorption coefficient	3.270 mm−1	
F(000)	514	
Crystal size	0.06 × 0.04 × 0.03 mm3	
Theta range for data collection	3.95 to 60.00 °.	
Index ranges	−9 <= h <= 9, −10 <= k <= 10, −13 <= l <= 13	
Reflections collected	21621	
Independent reflections	5333 [R(int) = 0.0472]	
Completeness to theta = 60.00 °	97.90%	
Absorption correction	Semi-empirical from equivalents	
Max. and min. transmission	0.9083 and 0.8279	
Refinement method	Full-matrix least-squares on F2	
Data / restraints / parameters	5333 / 3 / 544	
Goodness-of-fit on F2	1.024	
Final R indices [I > 2sigma(I)]	R1 = 0.0268, wR2 = 0.0601	
R indices (all data)	R1 = 0.0293, wR2 = 0.0611	
Absolute structure parameter	0.023(16)	
Extinction coefficient	0.0043(2)	

Table S9. Atomic coordinates (× 10^4) and equivalent isotropic displacement parameters (Å^2 × 10^3) for **1.** U(eq) is defined as one third of the trace of the orthogonalized U^{ij} tensor.

	x	y	z	U(eq)
Cu(1)	9216(1)	1531(1)	2326(1)	14(1)
Cu(2)	8637(1)	5238(1)	−737(1)	15(1)
S(1)	11516(1)	1160(1)	1499(1)	17(1)
S(2)	6345(1)	5538(1)	119(1)	18(1)
C(1)	8407(4)	4481(4)	2660(3)	14(1)
C(2)	6975(4)	4363(4)	3137(3)	15(1)
C(3)	6488(4)	3037(4)	3282(3)	14(1)
C(4)	4134(5)	4169(4)	4067(3)	13(1)
C(5)	4585(4)	5459(4)	3910(3)	14(1)
C(6)	6019(4)	5502(4)	3443(3)	17(1)
C(7)	2755(4)	4067(4)	4543(3)	17(1)
C(8)	1815(4)	5220(4)	4843(3)	18(1)
C(9)	2199(4)	6508(4)	4680(3)	16(1)
C(10)	3587(4)	6607(4)	4225(3)	16(1)
C(11)	1094(5)	7735(4)	4994(4)	22(1)
C(12)	11705(4)	2827(4)	1432(3)	15(1)
C(13)	9522(4)	8009(4)	−894(3)	15(1)
C(14)	10984(4)	7515(4)	−1349(3)	15(1)
C(15)	11496(4)	6102(4)	−1485(3)	12(1)
C(16)	13782(5)	6633(4)	−2309(3)	14(1)
C(17)	13337(4)	8039(4)	−2092(3)	16(1)
C(18)	11918(4)	8448(4)	−1613(3)	13(1)
C(19)	15143(5)	6147(4)	−2833(3)	18(1)
C(20)	16083(4)	7095(4)	−3103(3)	19(1)
C(21)	15710(4)	8513(4)	−2858(3)	20(1)
C(22)	14358(4)	8975(4)	−2377(3)	16(1)
C(23)	16790(5)	9510(5)	−3168(4)	29(1)
C(24)	6183(4)	7266(4)	231(3)	16(1)
N(1)	9399(4)	3501(3)	2345(3)	12(1)
N(2)	10715(3)	3844(3)	1904(3)	17(1)
N(3)	5115(3)	3042(3)	3738(2)	14(1)
N(4)	12858(3)	3191(3)	929(3)	18(1)
N(5)	8497(4)	7245(3)	−637(3)	14(1)
N(6)	7166(3)	7925(3)	−219(3)	16(1)
N(7)	12846(3)	5741(3)	−1974(2)	14(1)
N(8)	5021(4)	8038(3)	741(3)	18(1)
N(9)	3906(4)	2130(3)	6658(3)	22(1)
N(10)	9939(4)	1752(4)	5242(3)	24(1)
N(11)	1680(4)	7079(3)	1696(3)	16(1)
N(12)	6239(4)	1402(3)	179(2)	16(1)
O(1)	7247(3)	1920(3)	3024(2)	18(1)
O(2)	8813(3)	-473(3)	2060(2)	15(1)
O(3)	10800(3)	5200(3)	−1173(2)	16(1)
O(4)	9025(3)	3417(3)	−500(2)	17(1)
O(5)	7905(3)	4110(3)	−2708(2)	24(1)
O(6)	3544(3)	2824(3)	7691(2)	24(1)
O(7)	3173(3)	980(3)	6044(2)	25(1)
O(8)	4997(3)	2581(3)	6247(2)	27(1)
O(9)	9168(3)	966(3)	5633(2)	26(1)
O(10)	10424(3)	1248(3)	4253(2)	25(1)
O(11)	10235(4)	3026(3)	5872(2)	36(1)
O(12)	352(3)	6502(3)	1676(2)	20(1)
O(13)	2862(3)	6339(3)	1475(2)	19(1)
O(14)	1794(3)	8393(3)	1927(2)	24(1)

O(15)	5065(3)	854(3)	434(2)	22(1)
O(16)	7539(3)	815(3)	138(2)	21(1)
O(17)	6108(3)	2529(3)	−29(3)	35(1)
O(18)	7249(3)	9002(3)	3670(2)	22(1)
O(19)	4228(3)	276(3)	3795(2)	22(1)
O(20)	670(3)	1569(3)	8017(2)	22(1)

Table S10. Bond lengths [Å] and angles [°] for **1.**

Cu(1)-O(2)	1.943(3)
Cu(1)-O(1)	1.946(3)
Cu(1)-N(1)	1.969(3)
Cu(1)-S(1)	2.2776(10)
Cu(2)-O(4)	1.961(2)
Cu(2)-O(3)	1.973(3)
Cu(2)-N(5)	1.975(3)
Cu(2)-O(5)	2.254(2)
Cu(2)-S(2)	2.2888(10)
Cu(2)-H(4P)	2.0848
S(1)-C(12)	1.706(4)
S(2)-C(24)	1.694(4)
C(1)-N(1)	1.282(5)
C(1)-C(2)	1.430(5)
C(1)-H(1A)	0.95
C(2)-C(6)	1.373(5)
C(2)-C(3)	1.463(5)
C(3)-O(1)	1.255(4)
C(3)-N(3)	1.350(5)
C(4)-N(3)	1.380(5)
C(4)-C(7)	1.387(6)
C(4)-C(5)	1.425(5)
C(5)-C(10)	1.403(5)
C(5)-C(6)	1.412(5)
C(6)-H(6A)	0.95
C(7)-C(8)	1.374(5)
C(7)-H(7A)	0.95
C(8)-C(9)	1.411(5)
C(8)-H(8A)	0.95
C(9)-C(10)	1.379(5)
C(9)-C(11)	1.519(5)
C(10)-H(10A)	0.95
C(11)-H(11A)	0.98
C(11)-H(11B)	0.98
C(11)-H(11C)	0.98
C(12)-N(4)	1.326(5)
C(12)-N(2)	1.339(4)
C(13)-N(5)	1.291(5)
C(13)-C(14)	1.441(5)
C(13)-H(13A)	0.95
C(14)-C(18)	1.367(5)
C(14)-C(15)	1.441(5)
C(15)-O(3)	1.262(4)
C(15)-N(7)	1.348(4)
C(16)-N(7)	1.386(5)
C(16)-C(19)	1.395(5)
C(16)-C(17)	1.401(5)
C(17)-C(18)	1.403(5)
C(17)-C(22)	1.429(5)
C(18)-H(18A)	0.95
C(19)-C(20)	1.386(5)
C(19)-H(19A)	0.95
C(20)-C(21)	1.392(6)
C(20)-H(20A)	0.95
C(21)-C(22)	1.362(5)
C(21)-C(23)	1.523(6)

C(22)-H(22A)	0.95
C(23)-H(23A)	0.98
C(23)-H(23B)	0.98
C(23)-H(23C)	0.98
C(24)-N(8)	1.333(5)
C(24)-N(6)	1.336(5)
N(1)-N(2)	1.387(4)
N(2)-H(2N)	0.9
N(3)-H(3N)	0.9
N(4)-H(4M)	0.9
N(4)-H(4N)	0.9
N(5)-N(6)	1.381(4)
N(6)-H(6N)	0.9
N(7)-H(7N)	0.8999
N(8)-H(8M)	0.9
N(8)-H(8N)	0.9
N(9)-O(7)	1.251(4)
N(9)-O(8)	1.256(4)
N(9)-O(6)	1.274(4)
N(10)-O(10)	1.243(4)
N(10)-O(11)	1.246(4)
N(10)-O(9)	1.265(4)
N(11)-O(14)	1.248(4)
N(11)-O(13)	1.251(4)
N(11)-O(12)	1.265(4)
N(12)-O(17)	1.239(4)
N(12)-O(16)	1.252(4)
N(12)-O(15)	1.259(4)
O(2)-H(2O)	0.85
O(2)-H(2P)	0.85
O(4)-H(4O)	0.85
O(4)-H(4P)	0.85
O(5)-H(5O)	0.85
O(5)-H(5P)	0.85
O(18)-H(18O)	0.85
O(18)-H(18P)	0.85
O(19)-H(19O)	0.85
O(19)-H(19P)	0.8501
O(20)-H(20O)	0.85
O(20)-H(20P)	0.85
O(2)-Cu(1)-O(1)	88.29(11)
O(2)-Cu(1)-N(1)	170.60(12)
O(1)-Cu(1)-N(1)	90.80(12)
O(2)-Cu(1)-S(1)	93.83(8)
O(1)-Cu(1)-S(1)	177.77(8)
N(1)-Cu(1)-S(1)	86.99(10)
O(4)-Cu(2)-O(3)	87.56(11)
O(4)-Cu(2)-N(5)	167.95(11)
O(3)-Cu(2)-N(5)	90.48(12)
O(4)-Cu(2)-O(5)	91.00(10)
O(3)-Cu(2)-O(5)	84.96(10)
N(5)-Cu(2)-O(5)	100.67(11)
O(4)-Cu(2)-S(2)	93.35(8)
O(3)-Cu(2)-S(2)	169.23(8)
N(5)-Cu(2)-S(2)	86.40(9)
O(5)-Cu(2)-S(2)	105.74(7)
O(4)-Cu(2)-H(4P)	24
O(3)-Cu(2)-H(4P)	86.3

N(5)-Cu(2)-H(4P)	144
O(5)-Cu(2)-H(4P)	114.7
S(2)-Cu(2)-H(4P)	90.2
C(12)-S(1)-Cu(1)	95.49(13)
C(24)-S(2)-Cu(2)	95.27(13)
N(1)-C(1)-C(2)	125.8(3)
N(1)-C(1)-H(1A)	117.1
C(2)-C(1)-H(1A)	117.1
C(6)-C(2)-C(1)	119.7(3)
C(6)-C(2)-C(3)	119.1(3)
C(1)-C(2)-C(3)	121.2(3)
O(1)-C(3)-N(3)	118.6(3)
O(1)-C(3)-C(2)	125.1(3)
N(3)-C(3)-C(2)	116.3(3)
N(3)-C(4)-C(7)	121.5(3)
N(3)-C(4)-C(5)	117.9(3)
C(7)-C(4)-C(5)	120.5(3)
C(10)-C(5)-C(6)	123.7(3)
C(10)-C(5)-C(4)	118.5(3)
C(6)-C(5)-C(4)	117.7(3)
C(2)-C(6)-C(5)	122.8(3)
C(2)-C(6)-H(6A)	118.6
C(5)-C(6)-H(6A)	118.6
C(8)-C(7)-C(4)	118.9(4)
C(8)-C(7)-H(7A)	120.5
C(4)-C(7)-H(7A)	120.5
C(7)-C(8)-C(9)	122.4(3)
C(7)-C(8)-H(8A)	118.8
C(9)-C(8)-H(8A)	118.8
C(10)-C(9)-C(8)	118.2(3)
C(10)-C(9)-C(11)	121.6(3)
C(8)-C(9)-C(11)	120.2(3)
C(9)-C(10)-C(5)	121.4(3)
C(9)-C(10)-H(10A)	119.3
C(5)-C(10)-H(10A)	119.3
C(9)-C(11)-H(11A)	109.5
C(9)-C(11)-H(11B)	109.5
H(11A)-C(11)-H(11B)	109.5
C(9)-C(11)-H(11C)	109.5
H(11A)-C(11)-H(11C)	109.5
H(11B)-C(11)-H(11C)	109.5
N(4)-C(12)-N(2)	116.5(3)
N(4)-C(12)-S(1)	121.8(3)
N(2)-C(12)-S(1)	121.7(3)
N(5)-C(13)-C(14)	125.3(3)
N(5)-C(13)-H(13A)	117.4
C(14)-C(13)-H(13A)	117.4
C(18)-C(14)-C(13)	118.0(3)
C(18)-C(14)-C(15)	120.2(3)
C(13)-C(14)-C(15)	121.7(3)
O(3)-C(15)-N(7)	118.3(3)
O(3)-C(15)-C(14)	125.5(3)
N(7)-C(15)-C(14)	116.2(3)
N(7)-C(16)-C(19)	120.7(3)
N(7)-C(16)-C(17)	118.3(3)
C(19)-C(16)-C(17)	121.1(3)
C(16)-C(17)-C(18)	118.4(3)
C(16)-C(17)-C(22)	117.9(3)

C(18)-C(17)-C(22)	123.7(3)
C(14)-C(18)-C(17)	121.6(3)
C(14)-C(18)-H(18A)	119.2
C(17)-C(18)-H(18A)	119.2
C(20)-C(19)-C(16)	118.7(4)
C(20)-C(19)-H(19A)	120.7
C(16)-C(19)-H(19A)	120.7
C(19)-C(20)-C(21)	121.8(4)
C(19)-C(20)-H(20A)	119.1
C(21)-C(20)-H(20A)	119.1
C(22)-C(21)-C(20)	119.3(4)
C(22)-C(21)-C(23)	121.0(4)
C(20)-C(21)-C(23)	119.6(3)
C(21)-C(22)-C(17)	121.2(4)
C(21)-C(22)-H(22A)	119.4
C(17)-C(22)-H(22A)	119.4
C(21)-C(23)-H(23A)	109.5
C(21)-C(23)-H(23B)	109.5
H(23A)-C(23)-H(23B)	109.5
C(21)-C(23)-H(23C)	109.5
H(23A)-C(23)-H(23C)	109.5
H(23B)-C(23)-H(23C)	109.5
N(8)-C(24)-N(6)	115.8(3)
N(8)-C(24)-S(2)	121.6(3)
N(6)-C(24)-S(2)	122.6(3)
C(1)-N(1)-N(2)	116.2(3)
C(1)-N(1)-Cu(1)	127.6(3)
N(2)-N(1)-Cu(1)	116.0(2)
C(12)-N(2)-N(1)	119.5(3)
C(12)-N(2)-H(2N)	119.8
N(1)-N(2)-H(2N)	116.2
C(3)-N(3)-C(4)	126.2(3)
C(3)-N(3)-H(3N)	111.7
C(4)-N(3)-H(3N)	122
C(12)-N(4)-H(4M)	114.4
C(12)-N(4)-H(4N)	123.1
H(4M)-N(4)-H(4N)	121
C(13)-N(5)-N(6)	116.0(3)
C(13)-N(5)-Cu(2)	127.6(3)
N(6)-N(5)-Cu(2)	116.4(2)
C(24)-N(6)-N(5)	118.7(3)
C(24)-N(6)-H(6N)	126.6
N(5)-N(6)-H(6N)	109.8
C(15)-N(7)-C(16)	125.1(3)
C(15)-N(7)-H(7N)	112.1
C(16)-N(7)-H(7N)	122.3
C(24)-N(8)-H(8M)	119
C(24)-N(8)-H(8N)	120.1
H(8M)-N(8)-H(8N)	119.8
O(7)-N(9)-O(8)	119.4(3)
O(7)-N(9)-O(6)	119.8(3)
O(8)-N(9)-O(6)	120.9(3)
O(10)-N(10)-O(11)	120.7(3)
O(10)-N(10)-O(9)	120.2(3)
O(11)-N(10)-O(9)	119.1(3)
O(14)-N(11)-O(13)	120.5(3)
O(14)-N(11)-O(12)	119.2(3)
O(13)-N(11)-O(12)	120.3(3)

O(17)-N(12)-O(16)	119.8(3)
O(17)-N(12)-O(15)	119.8(3)
O(16)-N(12)-O(15)	120.4(3)
C(3)-O(1)-Cu(1)	129.4(2)
Cu(1)-O(2)-H(2O)	113.9
Cu(1)-O(2)-H(2P)	119.8
H(2O)-O(2)-H(2P)	92.2
C(15)-O(3)-Cu(2)	128.0(2)
Cu(2)-O(4)-H(4O)	117.7
Cu(2)-O(4)-H(4P)	86.2
H(4O)-O(4)-H(4P)	100.2
Cu(2)-O(5)-H(5O)	94.9
Cu(2)-O(5)-H(5P)	91.2
H(5O)-O(5)-H(5P)	117
H(18O)-O(18)-H(18P)	113.9
H(19O)-O(19)-H(19P)	106.2
H(20O)-O(20)-H(20P)	99.1

Table S11. Anisotropic displacement parameters ($Å^2 \times 10^3$) for **1.** The anisotropic displacement factor exponent takes the form: $-2\pi^2[h^2a \times {}^2U^{11} + ... + 2 h k a \times b \times U^{12}]$

	U11	U22	U33	U23	U13	U12
Cu(1)	14(1)	10(1)	20(1)	6(1)	5(1)	1(1)
Cu(2)	15(1)	10(1)	20(1)	6(1)	5(1)	0(1)
S(1)	16(1)	13(1)	24(1)	7(1)	6(1)	2(1)
S(2)	16(1)	13(1)	27(1)	9(1)	8(1)	1(1)
C(1)	13(2)	11(2)	21(2)	8(2)	4(2)	−1(2)
C(2)	15(2)	18(2)	13(2)	5(2)	2(2)	−1(2)
C(3)	17(2)	12(2)	14(2)	5(1)	3(2)	1(2)
C(4)	8(2)	19(2)	9(2)	2(2)	0(2)	1(2)
C(5)	17(2)	14(2)	8(2)	2(1)	0(2)	0(2)
C(6)	18(2)	12(2)	22(2)	8(2)	1(2)	0(2)
C(7)	14(2)	17(2)	21(2)	8(2)	0(2)	0(2)
C(8)	12(2)	25(2)	14(2)	5(2)	0(2)	−1(2)
C(9)	19(2)	14(2)	13(2)	2(1)	0(2)	2(2)
C(10)	20(2)	12(2)	18(2)	5(2)	5(2)	1(2)
C(11)	17(2)	20(2)	29(2)	7(2)	8(2)	6(2)
C(12)	20(2)	8(2)	16(2)	3(1)	1(2)	5(2)
C(13)	18(2)	11(2)	13(2)	2(1)	1(2)	2(2)
C(14)	12(2)	22(2)	11(2)	5(2)	0(2)	1(2)
C(15)	12(2)	15(2)	8(2)	3(1)	−2(1)	0(1)
C(16)	12(2)	18(2)	10(2)	4(2)	−1(2)	−3(2)
C(17)	15(2)	18(2)	16(2)	8(2)	1(2)	−1(2)
C(18)	15(2)	12(2)	13(2)	6(1)	5(2)	1(1)
C(19)	18(2)	19(2)	14(2)	3(2)	2(2)	3(2)
C(20)	13(2)	28(2)	14(2)	6(2)	1(2)	0(2)
C(21)	19(2)	33(3)	12(2)	11(2)	2(2)	0(2)
C(22)	16(2)	16(2)	17(2)	7(2)	2(2)	−2(2)
C(23)	20(3)	36(3)	38(3)	21(2)	11(2)	−1(2)
C(24)	17(2)	17(2)	12(2)	4(2)	0(2)	−3(2)
N(1)	9(2)	13(2)	14(2)	5(1)	5(1)	−3(1)
N(2)	17(2)	17(2)	22(2)	10(1)	4(1)	3(1)
N(3)	14(2)	13(2)	18(2)	7(1)	5(1)	2(1)
N(4)	15(2)	14(2)	28(2)	10(1)	12(1)	2(1)
N(5)	10(2)	16(2)	13(2)	2(1)	0(1)	4(1)
N(6)	15(2)	14(2)	20(2)	5(1)	6(1)	1(1)
N(7)	11(2)	13(2)	18(2)	5(1)	4(1)	1(1)
N(8)	19(2)	13(2)	27(2)	9(1)	8(1)	3(1)
N(9)	20(2)	18(2)	32(2)	14(2)	4(2)	6(1)
N(10)	20(2)	28(2)	23(2)	7(2)	6(2)	0(1)
N(11)	18(2)	13(2)	19(2)	7(1)	3(1)	−2(1)
N(12)	18(2)	11(2)	20(2)	6(1)	4(1)	2(1)
O(1)	16(2)	12(2)	28(2)	10(1)	8(1)	1(1)
O(2)	17(2)	13(1)	20(1)	8(1)	6(1)	3(1)
O(3)	18(2)	12(2)	22(1)	8(1)	5(1)	2(1)
O(4)	20(2)	11(1)	24(1)	8(1)	5(1)	−3(1)
O(5)	17(2)	24(2)	28(1)	4(1)	2(1)	1(1)
O(6)	28(2)	23(2)	21(2)	6(1)	6(1)	3(1)
O(7)	26(2)	16(2)	30(1)	4(1)	3(1)	−8(1)
O(8)	24(2)	28(2)	31(2)	13(1)	5(1)	−7(1)
O(9)	26(2)	30(2)	26(2)	11(1)	6(1)	−10(1)
O(10)	23(2)	36(2)	16(1)	6(1)	6(1)	−2(1)
O(11)	48(2)	17(2)	39(2)	1(1)	21(2)	−6(1)
O(12)	13(2)	18(2)	32(2)	12(1)	9(1)	1(1)
O(13)	13(2)	18(2)	30(2)	11(1)	8(1)	6(1)
O(14)	20(2)	12(2)	41(2)	10(1)	5(1)	2(1)

O(15)	21(2)	15(2)	36(2)	12(1)	13(1)	1(1)
O(16)	17(2)	15(2)	32(2)	8(1)	7(1)	7(1)
O(17)	24(2)	24(2)	74(2)	34(2)	13(2)	5(1)
O(18)	18(2)	21(2)	27(2)	9(1)	2(1)	−2(1)
O(19)	19(2)	24(2)	23(2)	8(1)	4(1)	−3(1)
O(20)	26(2)	19(2)	22(1)	9(1)	5(1)	2(1)

Table S12. Hydrogen coordinates (× 10^4) and isotropic displacement parameters (Å^2 × 10^3) for **1**.

	x	y	z	U(eq)
H(1A)	8643	5362	2568	17
H(6A)	6336	6356	3336	20
H(7A)	2464	3213	4661	20
H(8A)	873	5149	5172	21
H(10A)	3873	7471	4122	20
H(11A)	1589	8571	4905	32
H(11B)	885	7963	5821	32
H(11C)	96	7463	4457	32
H(13A)	9294	8974	−772	18
H(18A)	11599	9396	−1469	15
H(19A)	15420	5184	−3000	21
H(20A)	17007	6768	−3465	22
H(22A)	14089	9936	−2228	19
H(23A)	16800	10470	−2574	43
H(23B)	16398	9538	−3961	43
H(23C)	17866	9165	−3171	43
H(2N)	10653	4616	1681	26
H(3N)	4869	2187	3780	21
H(4M)	13518	2491	635	27
H(4N)	13113	4100	1024	27
H(6N)	7320	8872	−25	24
H(7N)	13010	4805	−2160	21
H(8M)	5014	8968	830	28
H(8N)	4372	7683	1119	28
H(2O)	9641	−953	2007	23
H(2P)	8561	−725	2625	23
H(4O)	8256	2995	−349	26
H(4P)	9429	3911	205	26
H(5O)	7202	3567	−2616	36
H(5P)	8821	3784	−2795	36
H(18O)	7962	9471	4226	33
H(18P)	6355	9378	3705	33
H(19O)	3438	−205	3327	32
H(19P)	4012	434	4500	32
H(20O)	76	1405	7359	33
H(20P)	1456	1977	7871	33

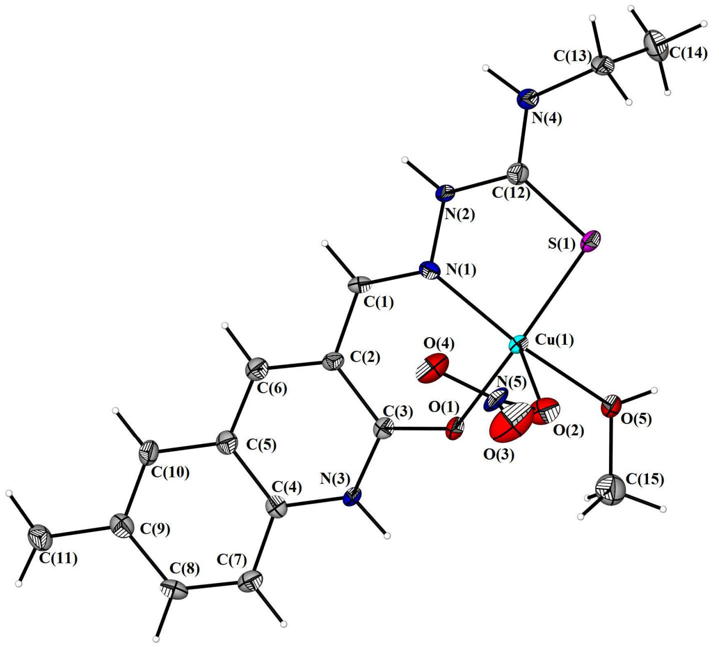

3a

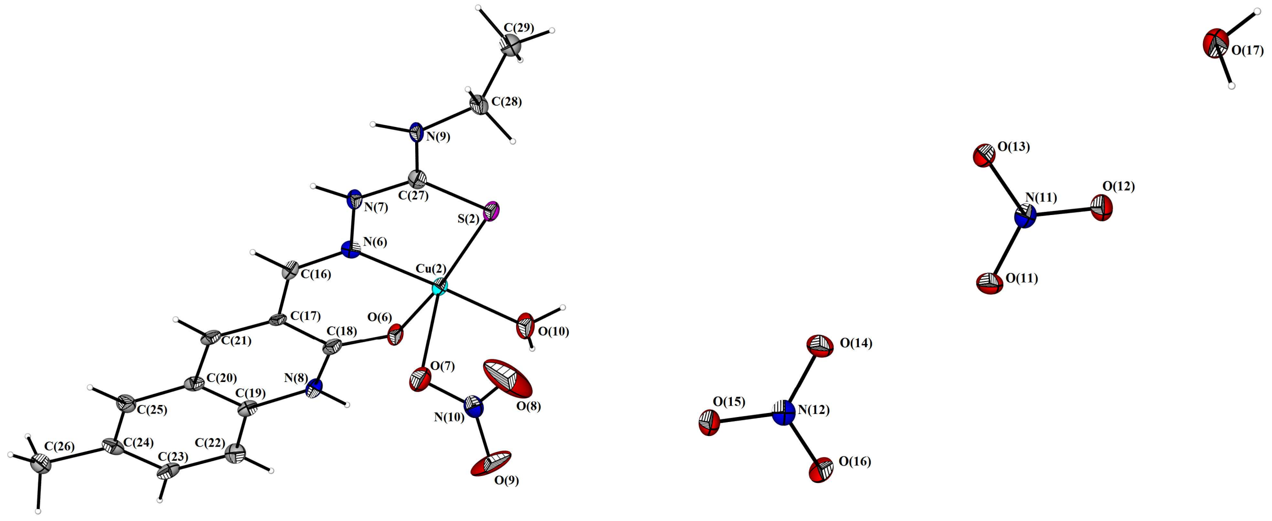

3b

Figure S12. 3a, **3b**, nitrate counter ions and clathrated, water molecule with the numbering.

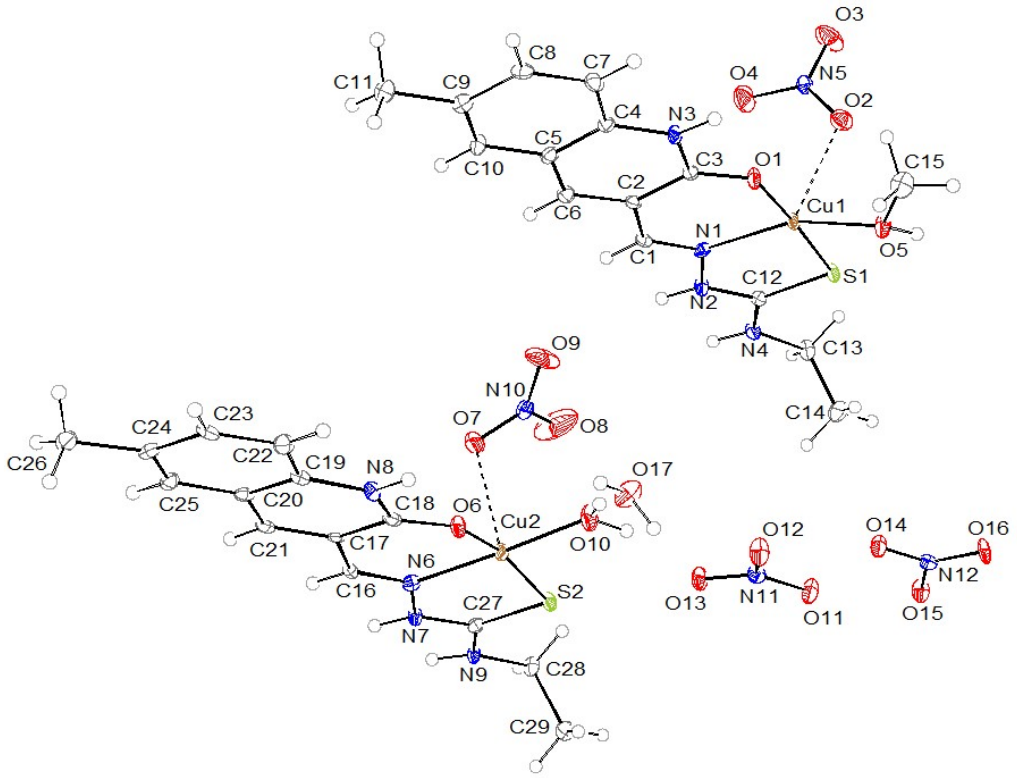

Figure S13. ORTEP view of the unit cell of **3**.

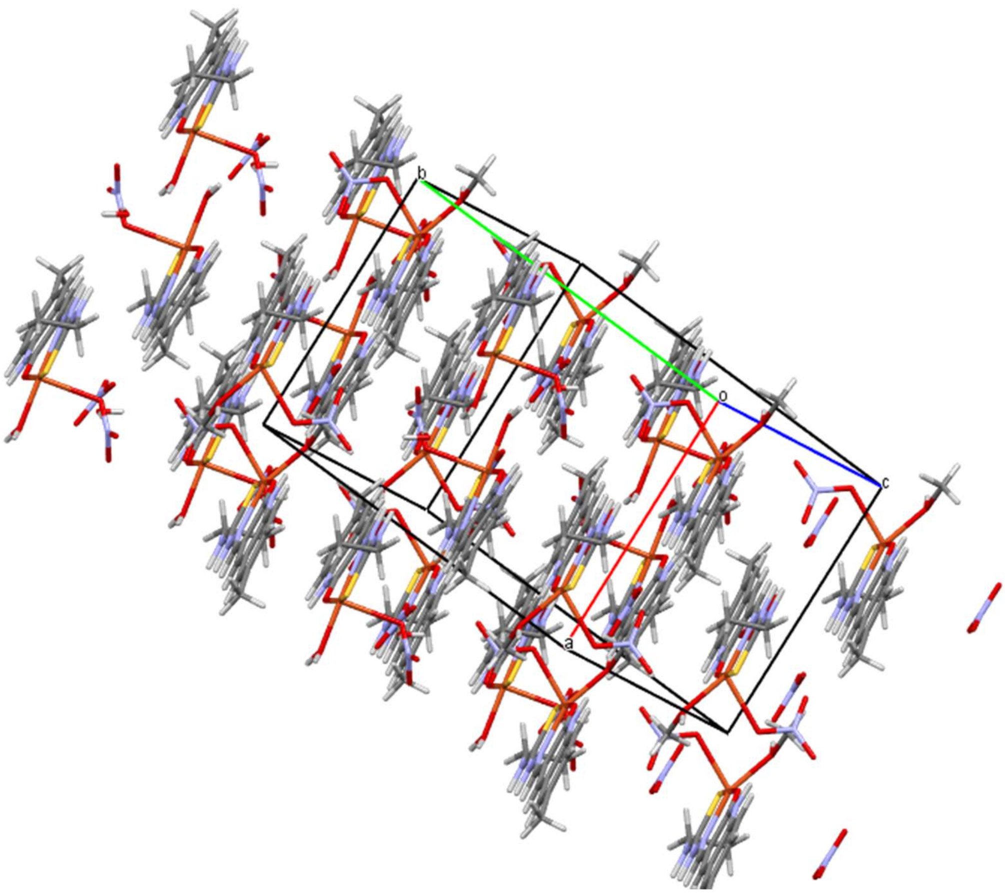

Figure S14. Molecular Packing of **3**.

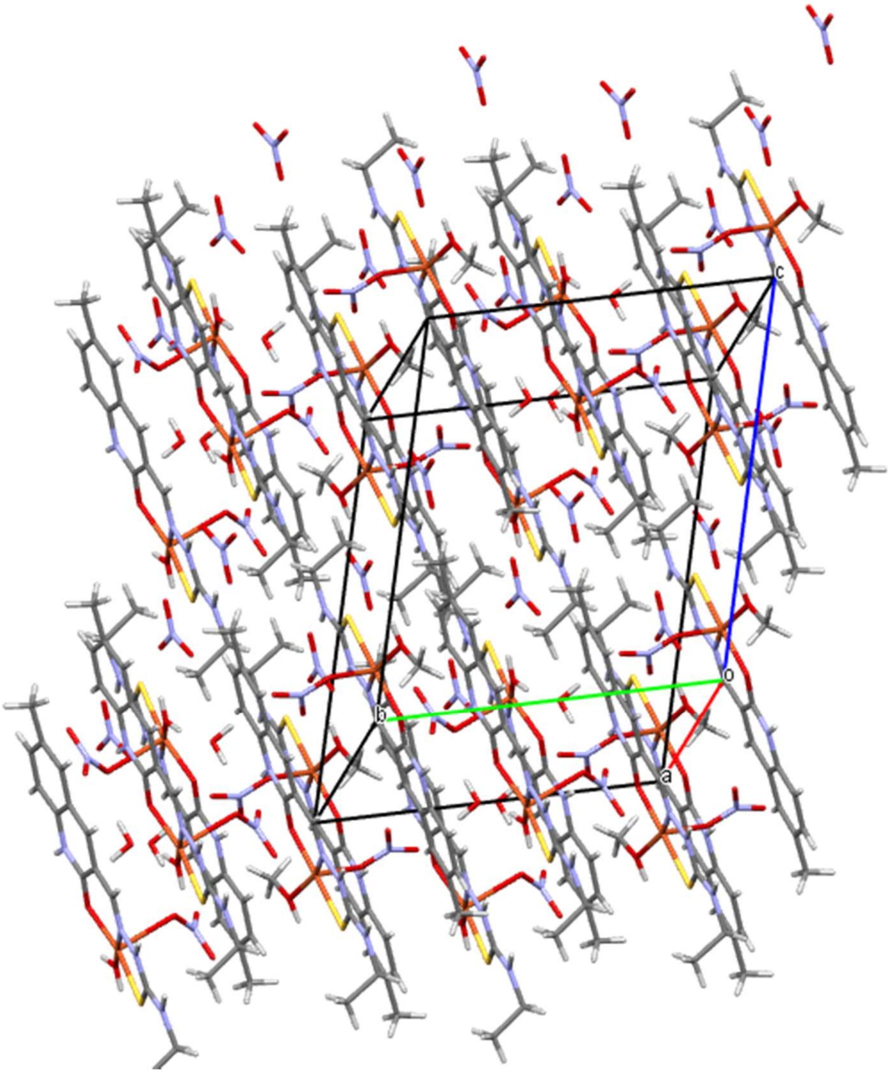

Figure S15. Molecular Packing of **3**.

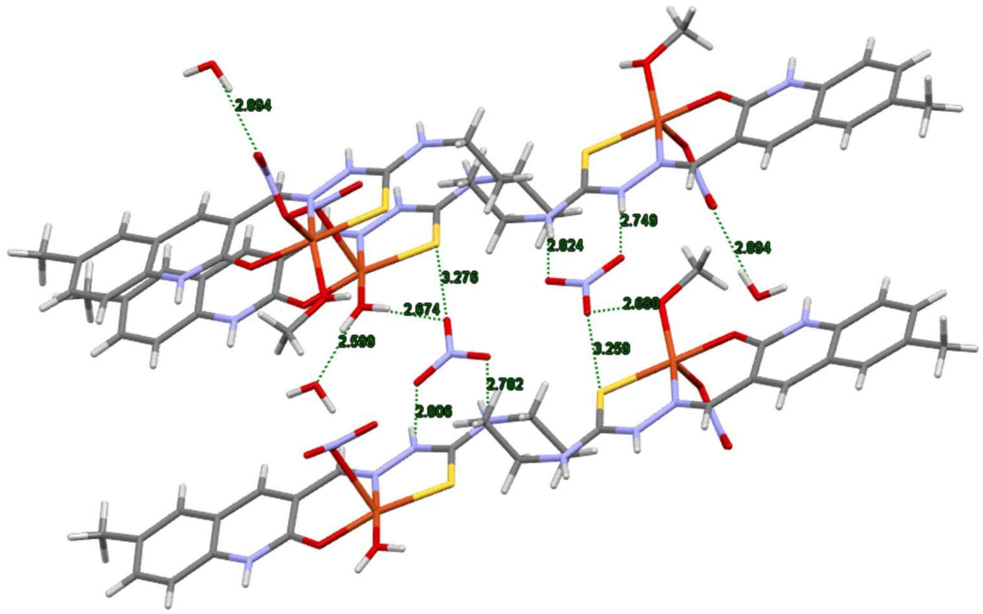

Figure S16. Selected short H-bonding interactions in **3**.

A suitable crystal (a green block with dimensions 0.08 × 0.06 × 0.04 mm^3) was mounted on a nylon loop and placed in a cold nitrogen stream at 110(2) K on a BRUKER GADDS diffractometer (controlled using the FRAMBO software, v.4.1.05) for the crystal screening, unit cell determination, and data collection. Experimental set up and data collection parameters were as described previously.

Data reduction, structure solution and refinement were carried out as for the previous structure. Statistical tests indicated a centrosymmetric space group (*P*−1). This choice was later confirmed by the successful refinement.

At the end of the refinement, we noted that the thermal parameters of the O8 and O9 atoms were significantly elongated indicating a possible disorder. However, no model for disorder was found, that could improve the description or the agreement factors significantly.

A clathrated water molecule was found from the Fourier difference maps.

Table S13. Crystal data and structure refinement for **3.**

Empirical formula	$C_{29}H_{40}Cu_2N_{12}O_{17}S_2$	
Formula weight	1019.93	
Temperature	110 (2) K	
Wavelength	1.54178 Å	
Crystal system	Triclinic	
Space group	P−1	
Unit cell dimensions	a = 10.3073(5) Å	α= 109.017(4)°
	b = 13.3389(7) Å	β= 94.252(4)°
	c = 15.2916(8) Å	γ = 94.665(4)°
Volume	1969.75(17) Å3	
Z	2	
Density (calculated)	1.720 Mg/m3	
Absorption coefficient	3.123 mm^{-1}	
F(000)	1048	
Crystal size	0.08 x 0.06 x 0.04 mm^3	
Theta range for data collection	3.53 to 59.99°	
Index ranges	−11 <= h <= 11, −14 <= k <= 14, −17 <= l <= 17	
Reflections collected	30628	
Independent reflections	5585 [R(int) = 0.0677]	
Completeness to theta = 59.99°	95.50%	
Absorption correction	Semi-empirical from equivalents	
Max. and min. transmission	0.8853 and 0.7882	
Refinement method	Full-matrix least-squares on F2	
Data / restraints / parameters	5585 / 0 / 564	
Goodness-of-fit on F2	1.047	
Final R indices [I > 2sigma(I)]	R1 = 0.0484, wR2 = 0.1250	
R indices (all data)	R1 = 0.0630, wR2 = 0.1303	

Table S14. Atomic coordinates (× 10^4) and equivalent isotropic displacement parameters (Å^2 × 10^3) for **3**. U(eq) is defined as one third of the trace of the orthogonalized U^{ij} tensor.

	x	y	z	U(eq)
Cu(1)	1734(1)	9977(1)	1706(1)	15(1)
Cu(2)	6881(1)	5162(1)	1839(1)	16(1)
S(1)	2174(1)	10912(1)	3242(1)	17(1)
S(2)	7330(1)	5988(1)	3389(1)	17(1)
C(1)	4250(4)	9348(3)	996(3)	14(1)
C(2)	3619(4)	8804(3)	65(3)	13(1)
C(3)	2231(4)	8748(3)	−160(3)	13(1)
C(4)	2483(4)	7732(3)	−1757(3)	14(1)
C(5)	3827(4)	7755(3)	−1550(3)	17(1)
C(6)	4383(4)	8304(3)	−619(3)	17(1)
C(7)	1875(4)	7212(3)	−2661(3)	18(1)
C(8)	2639(4)	6740(3)	−3348(3)	19(1)
C(9)	4002(4)	6732(3)	−3171(3)	19(1)
C(10)	4583(4)	7240(3)	−2275(3)	19(1)
C(11)	4780(4)	6174(3)	−3943(3)	22(1)
C(12)	3816(4)	10834(3)	3293(3)	16(1)
C(13)	4172(4)	11834(3)	4977(3)	20(1)
C(14)	3686(5)	11089(4)	5471(3)	28(1)
C(15)	−1151(4)	9137(4)	1011(3)	32(1)
C(16)	9323(4)	4463(3)	1084(3)	13(1)
C(17)	8648(4)	3944(3)	164(3)	12(1)
C(18)	7242(4)	3825(3)	−10(3)	16(1)
C(19)	7417(4)	2814(3)	−1627(3)	16(1)
C(20)	8778(4)	2934(3)	−1480(3)	14(1)
C(21)	9381(4)	3498(3)	−558(3)	15(1)
C(22)	6751(4)	2254(3)	−2516(3)	20(1)
C(23)	7487(4)	1803(3)	−3235(3)	19(1)
C(24)	8865(4)	1898(3)	−3109(3)	19(1)
C(25)	9498(4)	2464(3)	−2241(3)	18(1)
C(26)	9620(4)	1335(3)	−3901(3)	20(1)
C(27)	8961(4)	5869(3)	3421(3)	17(1)
C(28)	9367(4)	6897(3)	5095(3)	18(1)
C(29)	8830(4)	6197(3)	5627(3)	24(1)
N(1)	3638(3)	9846(2)	1691(2)	14(1)
N(2)	4397(3)	10311(3)	2532(2)	16(1)
N(3)	1752(3)	8237(2)	−1053(2)	14(1)
N(4)	4609(3)	11265(3)	4071(2)	17(1)
N(5)	1588(3)	12069(3)	1268(2)	23(1)
N(6)	8745(3)	4914(2)	1812(2)	15(1)
N(7)	9518(3)	5332(2)	2651(2)	15(1)
N(8)	6719(3)	3261(3)	−881(2)	15(1)
N(9)	9772(3)	6274(3)	4195(2)	16(1)
N(10)	6745(4)	7347(3)	1540(3)	23(1)
N(11)	2961(3)	5860(3)	3509(2)	17(1)
N(12)	2178(3)	9206(3)	6622(2)	17(1)
O(1)	1420(3)	9132(2)	403(2)	19(1)
O(2)	811(3)	11396(2)	1448(2)	28(1)
O(3)	1222(3)	12911(3)	1266(3)	44(1)
O(4)	2674(3)	11791(3)	1048(2)	39(1)
O(5)	−136(3)	9570(2)	1802(2)	23(1)
O(6)	6461(3)	4191(2)	582(2)	18(1)
O(7)	7453(3)	6618(2)	1304(2)	29(1)
O(8)	6885(6)	7976(3)	2334(3)	91(2)
O(9)	5937(3)	7473(3)	967(3)	60(1)

O(10)	5068(3)	5429(2)	1834(2)	26(1)
O(11)	2474(3)	6262(2)	4251(2)	26(1)
O(12)	2250(3)	5349(2)	2774(2)	26(1)
O(13)	4169(3)	5982(2)	3502(2)	21(1)
O(14)	2654(3)	8794(2)	5875(2)	24(1)
O(15)	2920(3)	9598(2)	7377(2)	24(1)
O(16)	972(3)	9205(2)	6623(2)	24(1)
O(17)	3084(3)	4271(2)	710(2)	32(1)

Table S15. Bond lengths [Å] and angles [°] for **3**.

Cu(1)-O(1)	1.930(3)
Cu(1)-N(1)	1.986(3)
Cu(1)-O(5)	1.989(3)
Cu(1)-S(1)	2.2632(11)
Cu(1)-O(2)	2.318(3)
Cu(2)-O(6)	1.929(3)
Cu(2)-O(10)	1.931(3)
Cu(2)-N(6)	1.977(3)
Cu(2)-S(2)	2.2582(11)
Cu(2)-O(7)	2.388(3)
S(1)-C(12)	1.701(4)
S(2)-C(27)	1.700(4)
C(1)-N(1)	1.295(5)
C(1)-C(2)	1.445(5)
C(1)-H(1A)	0.93
C(2)-C(6)	1.383(5)
C(2)-C(3)	1.437(5)
C(3)-O(1)	1.261(4)
C(3)-N(3)	1.346(5)
C(4)-N(3)	1.382(5)
C(4)-C(5)	1.394(6)
C(4)-C(7)	1.401(5)
C(5)-C(6)	1.422(5)
C(5)-C(10)	1.424(6)
C(6)-H(6A)	0.93
C(7)-C(8)	1.375(6)
C(7)-H(7A)	0.93
C(8)-C(9)	1.413(6)
C(8)-H(8A)	0.93
C(9)-C(10)	1.380(6)
C(9)-C(11)	1.501(5)
C(10)-H(10A)	0.93
C(11)-H(11A)	0.96
C(11)-H(11B)	0.96
C(11)-H(11C)	0.96
C(12)-N(4)	1.321(5)
C(12)-N(2)	1.358(5)
C(13)-N(4)	1.470(5)
C(13)-C(14)	1.509(6)
C(13)-H(13A)	0.97
C(13)-H(13B)	0.97
C(14)-H(14A)	0.96
C(14)-H(14B)	0.96
C(14)-H(14C)	0.96
C(15)-O(5)	1.466(5)
C(15)-H(15A)	0.96
C(15)-H(15B)	0.96
C(15)-H(15C)	0.96
C(16)-N(6)	1.295(5)
C(16)-C(17)	1.444(5)
C(16)-H(16A)	0.93
C(17)-C(21)	1.382(5)
C(17)-C(18)	1.441(5)
C(18)-O(6)	1.261(5)
C(18)-N(8)	1.344(5)
C(19)-N(8)	1.387(5)

C(19)-C(20)	1.392(6)
C(19)-C(22)	1.411(6)
C(20)-C(25)	1.423(5)
C(20)-C(21)	1.430(5)
C(21)-H(21A)	0.93
C(22)-C(23)	1.383(6)
C(22)-H(22A)	0.93
C(23)-C(24)	1.409(6)
C(23)-H(23A)	0.93
C(24)-C(25)	1.377(6)
C(24)-C(26)	1.510(5)
C(25)-H(25A)	0.93
C(26)-H(26A)	0.96
C(26)-H(26B)	0.96
C(26)-H(26C)	0.96
C(27)-N(9)	1.325(5)
C(27)-N(7)	1.359(5)
C(28)-N(9)	1.468(5)
C(28)-C(29)	1.522(5)
C(28)-H(28A)	0.97
C(28)-H(28B)	0.97
C(29)-H(29A)	0.96
C(29)-H(29B)	0.96
C(29)-H(29C)	0.96
N(1)-N(2)	1.379(4)
N(2)-H(2N)	0.8999
N(3)-H(3N)	0.9001
N(4)-H(4N)	0.9
N(5)-O(3)	1.214(5)
N(5)-O(4)	1.244(4)
N(5)-O(2)	1.264(4)
N(6)-N(7)	1.380(4)
N(7)-H(7N)	0.9
N(8)-H(8N)	0.9
N(9)-H(9N)	0.9
N(10)-O(8)	1.219(5)
N(10)-O(9)	1.225(5)
N(10)-O(7)	1.238(5)
N(11)-O(13)	1.243(4)
N(11)-O(11)	1.248(4)
N(11)-O(12)	1.251(4)
N(12)-O(16)	1.243(4)
N(12)-O(14)	1.251(4)
N(12)-O(15)	1.265(4)
O(5)-H(5O)	0.85
O(10)-H(10O)	0.85
O(10)-H(10P)	0.8501
O(17)-H(17O)	0.8499
O(17)-H(17P)	0.85
O(1)-Cu(1)-N(1)	90.78(12)
O(1)-Cu(1)-O(5)	87.45(11)
N(1)-Cu(1)-O(5)	157.64(13)
O(1)-Cu(1)-S(1)	177.16(9)
N(1)-Cu(1)-S(1)	87.04(9)
O(5)-Cu(1)-S(1)	93.88(8)
O(1)-Cu(1)-O(2)	91.84(11)
N(1)-Cu(1)-O(2)	122.28(12)
O(5)-Cu(1)-O(2)	80.07(11)

S(1)-Cu(1)-O(2)	90.86(8)
O(6)-Cu(2)-O(10)	88.00(12)
O(6)-Cu(2)-N(6)	91.27(12)
O(10)-Cu(2)-N(6)	178.02(12)
O(6)-Cu(2)-S(2)	168.12(9)
O(10)-Cu(2)-S(2)	93.69(9)
N(6)-Cu(2)-S(2)	87.40(10)
O(6)-Cu(2)-O(7)	91.76(11)
O(10)-Cu(2)-O(7)	91.73(11)
N(6)-Cu(2)-O(7)	86.45(12)
S(2)-Cu(2)-O(7)	99.93(8)
C(12)-S(1)-Cu(1)	96.06(14)
C(27)-S(2)-Cu(2)	95.74(14)
N(1)-C(1)-C(2)	123.9(4)
N(1)-C(1)-H(1A)	118.1
C(2)-C(1)-H(1A)	118.1
C(6)-C(2)-C(3)	119.6(4)
C(6)-C(2)-C(1)	118.3(4)
C(3)-C(2)-C(1)	122.1(4)
O(1)-C(3)-N(3)	117.1(4)
O(1)-C(3)-C(2)	126.0(4)
N(3)-C(3)-C(2)	117.0(4)
N(3)-C(4)-C(5)	118.8(3)
N(3)-C(4)-C(7)	120.2(4)
C(5)-C(4)-C(7)	121.0(4)
C(4)-C(5)-C(6)	118.1(4)
C(4)-C(5)-C(10)	118.9(4)
C(6)-C(5)-C(10)	122.9(4)
C(2)-C(6)-C(5)	121.3(4)
C(2)-C(6)-H(6A)	119.4
C(5)-C(6)-H(6A)	119.4
C(8)-C(7)-C(4)	118.5(4)
C(8)-C(7)-H(7A)	120.7
C(4)-C(7)-H(7A)	120.7
C(7)-C(8)-C(9)	122.6(4)
C(7)-C(8)-H(8A)	118.7
C(9)-C(8)-H(8A)	118.7
C(10)-C(9)-C(8)	118.1(4)
C(10)-C(9)-C(11)	121.6(4)
C(8)-C(9)-C(11)	120.3(4)
C(9)-C(10)-C(5)	120.8(4)
C(9)-C(10)-H(10A)	119.6
C(5)-C(10)-H(10A)	119.6
C(9)-C(11)-H(11A)	109.5
C(9)-C(11)-H(11B)	109.5
H(11A)-C(11)-H(11B)	109.5
C(9)-C(11)-H(11C)	109.5
H(11A)-C(11)-H(11C)	109.5
H(11B)-C(11)-H(11C)	109.5
N(4)-C(12)-N(2)	115.4(4)
N(4)-C(12)-S(1)	122.8(3)
N(2)-C(12)-S(1)	121.8(3)
N(4)-C(13)-C(14)	112.6(3)
N(4)-C(13)-H(13A)	109.1
C(14)-C(13)-H(13A)	109.1
N(4)-C(13)-H(13B)	109.1
C(14)-C(13)-H(13B)	109.1
H(13A)-C(13)-H(13B)	107.8

C(13)-C(14)-H(14A)	109.5
C(13)-C(14)-H(14B)	109.5
H(14A)-C(14)-H(14B)	109.5
C(13)-C(14)-H(14C)	109.5
H(14A)-C(14)-H(14C)	109.5
H(14B)-C(14)-H(14C)	109.5
O(5)-C(15)-H(15A)	109.5
O(5)-C(15)-H(15B)	109.5
H(15A)-C(15)-H(15B)	109.5
O(5)-C(15)-H(15C)	109.5
H(15A)-C(15)-H(15C)	109.5
H(15B)-C(15)-H(15C)	109.5
N(6)-C(16)-C(17)	124.0(4)
N(6)-C(16)-H(16A)	118
C(17)-C(16)-H(16A)	118
C(21)-C(17)-C(18)	119.5(4)
C(21)-C(17)-C(16)	118.4(4)
C(18)-C(17)-C(16)	122.0(4)
O(6)-C(18)-N(8)	117.3(4)
O(6)-C(18)-C(17)	126.0(4)
N(8)-C(18)-C(17)	116.7(4)
N(8)-C(19)-C(20)	118.7(3)
N(8)-C(19)-C(22)	120.2(4)
C(20)-C(19)-C(22)	121.1(4)
C(19)-C(20)-C(25)	118.9(4)
C(19)-C(20)-C(21)	117.8(4)
C(25)-C(20)-C(21)	123.3(4)
C(17)-C(21)-C(20)	121.6(4)
C(17)-C(21)-H(21A)	119.2
C(20)-C(21)-H(21A)	119.2
C(23)-C(22)-C(19)	118.2(4)
C(23)-C(22)-H(22A)	120.9
C(19)-C(22)-H(22A)	120.9
C(22)-C(23)-C(24)	122.2(4)
C(22)-C(23)-H(23A)	118.9
C(24)-C(23)-H(23A)	118.9
C(25)-C(24)-C(23)	118.8(4)
C(25)-C(24)-C(26)	121.0(4)
C(23)-C(24)-C(26)	120.2(4)
C(24)-C(25)-C(20)	120.8(4)
C(24)-C(25)-H(25A)	119.6
C(20)-C(25)-H(25A)	119.6
C(24)-C(26)-H(26A)	109.5
C(24)-C(26)-H(26B)	109.5
H(26A)-C(26)-H(26B)	109.5
C(24)-C(26)-H(26C)	109.5
H(26A)-C(26)-H(26C)	109.5
H(26B)-C(26)-H(26C)	109.5
N(9)-C(27)-N(7)	115.3(4)
N(9)-C(27)-S(2)	122.7(3)
N(7)-C(27)-S(2)	121.9(3)
N(9)-C(28)-C(29)	112.7(3)
N(9)-C(28)-H(28A)	109.1
C(29)-C(28)-H(28A)	109.1
N(9)-C(28)-H(28B)	109.1
C(29)-C(28)-H(28B)	109.1
H(28A)-C(28)-H(28B)	107.8
C(28)-C(29)-H(29A)	109.5

C(28)-C(29)-H(29B)	109.5
H(29A)-C(29)-H(29B)	109.5
C(28)-C(29)-H(29C)	109.5
H(29A)-C(29)-H(29C)	109.5
H(29B)-C(29)-H(29C)	109.5
C(1)-N(1)-N(2)	115.9(3)
C(1)-N(1)-Cu(1)	128.0(3)
N(2)-N(1)-Cu(1)	116.1(2)
C(12)-N(2)-N(1)	119.0(3)
C(12)-N(2)-H(2N)	124.9
N(1)-N(2)-H(2N)	116
C(3)-N(3)-C(4)	125.2(3)
C(3)-N(3)-H(3N)	112.8
C(4)-N(3)-H(3N)	121.8
C(12)-N(4)-C(13)	124.0(4)
C(12)-N(4)-H(4N)	116.4
C(13)-N(4)-H(4N)	119.6
O(3)-N(5)-O(4)	123.7(4)
O(3)-N(5)-O(2)	119.5(4)
O(4)-N(5)-O(2)	116.7(4)
C(16)-N(6)-N(7)	117.1(3)
C(16)-N(6)-Cu(2)	126.8(3)
N(7)-N(6)-Cu(2)	115.9(2)
C(27)-N(7)-N(6)	118.8(3)
C(27)-N(7)-H(7N)	127.4
N(6)-N(7)-H(7N)	113.6
C(18)-N(8)-C(19)	125.6(3)
C(18)-N(8)-H(8N)	118.1
C(19)-N(8)-H(8N)	116.3
C(27)-N(9)-C(28)	123.7(3)
C(27)-N(9)-H(9N)	109.7
C(28)-N(9)-H(9N)	126.2
O(8)-N(10)-O(9)	119.3(5)
O(8)-N(10)-O(7)	120.5(4)
O(9)-N(10)-O(7)	120.2(4)
O(13)-N(11)-O(11)	119.4(3)
O(13)-N(11)-O(12)	119.8(3)
O(11)-N(11)-O(12)	120.8(3)
O(16)-N(12)-O(14)	119.8(3)
O(16)-N(12)-O(15)	120.2(3)
O(14)-N(12)-O(15)	120.0(3)
C(3)-O(1)-Cu(1)	129.2(3)
N(5)-O(2)-Cu(1)	115.9(3)
C(15)-O(5)-Cu(1)	124.9(2)
C(15)-O(5)-H(5O)	113.5
Cu(1)-O(5)-H(5O)	114.1
C(18)-O(6)-Cu(2)	127.8(3)
N(10)-O(7)-Cu(2)	114.0(2)
Cu(2)-O(10)-H(10O)	127.2
Cu(2)-O(10)-H(10P)	118.9
H(10O)-O(10)-H(10P)	109.4
H(17O)-O(17)-H(17P)	99.9

Table S16. Anisotropic displacement parameters ($Å^2 \times 10^3$) for **3**. The anisotropic displacement factor exponent takes the form: $-2\pi^2[h^2a \times {}^2U^{11} + ... + 2 h k a \times b \times U^{12}]$.

	U11	U22	U33	U23	U13	U12
Cu(1)	10(1)	21(1)	14(1)	5(1)	1(1)	4(1)
Cu(2)	10(1)	21(1)	17(1)	7(1)	2(1)	4(1)
S(1)	10(1)	22(1)	18(1)	4(1)	2(1)	6(1)
S(2)	12(1)	21(1)	18(1)	5(1)	2(1)	5(1)
C(1)	12(2)	14(2)	15(2)	6(2)	−3(2)	1(2)
C(2)	12(2)	15(2)	15(2)	8(2)	−1(2)	2(2)
C(3)	13(2)	11(2)	17(2)	7(2)	2(2)	1(2)
C(4)	17(2)	11(2)	14(2)	5(2)	2(2)	2(2)
C(5)	17(2)	19(2)	18(2)	10(2)	4(2)	0(2)
C(6)	16(2)	18(2)	19(2)	8(2)	2(2)	2(2)
C(7)	14(2)	21(2)	22(2)	10(2)	−1(2)	1(2)
C(8)	24(3)	19(2)	13(2)	6(2)	−4(2)	2(2)
C(9)	22(3)	18(2)	17(2)	6(2)	2(2)	1(2)
C(10)	14(2)	23(2)	19(2)	6(2)	5(2)	1(2)
C(11)	25(3)	20(2)	16(2)	0(2)	3(2)	0(2)
C(12)	15(2)	18(2)	17(2)	8(2)	2(2)	3(2)
C(13)	16(2)	24(2)	12(2)	−2(2)	−1(2)	2(2)
C(14)	33(3)	33(3)	20(3)	8(2)	10(2)	7(2)
C(15)	31(3)	33(3)	29(3)	7(2)	4(2)	0(2)
C(16)	10(2)	8(2)	20(2)	4(2)	1(2)	0(2)
C(17)	13(2)	12(2)	17(2)	11(2)	1(2)	3(2)
C(18)	15(2)	15(2)	23(2)	12(2)	1(2)	3(2)
C(19)	18(2)	15(2)	19(2)	8(2)	0(2)	4(2)
C(20)	14(2)	13(2)	19(2)	10(2)	−1(2)	−2(2)
C(21)	14(2)	13(2)	23(2)	11(2)	−1(2)	1(2)
C(22)	16(2)	22(2)	22(2)	10(2)	1(2)	0(2)
C(23)	22(3)	13(2)	21(2)	7(2)	−4(2)	5(2)
C(24)	25(3)	18(2)	15(2)	10(2)	3(2)	0(2)
C(25)	17(2)	21(2)	20(2)	11(2)	4(2)	1(2)
C(26)	24(3)	21(2)	18(2)	7(2)	4(2)	2(2)
C(27)	16(2)	15(2)	23(2)	10(2)	6(2)	0(2)
C(28)	17(2)	26(2)	9(2)	2(2)	0(2)	2(2)
C(29)	24(3)	26(3)	23(2)	9(2)	4(2)	4(2)
N(1)	14(2)	16(2)	11(2)	5(2)	−1(2)	−2(2)
N(2)	10(2)	22(2)	11(2)	−1(2)	−1(1)	1(2)
N(3)	7(2)	20(2)	15(2)	4(2)	1(2)	0(2)
N(4)	15(2)	21(2)	15(2)	4(2)	1(2)	5(2)
N(5)	7(2)	35(3)	32(2)	19(2)	1(2)	0(2)
N(6)	15(2)	13(2)	15(2)	5(2)	−1(2)	−2(2)
N(7)	10(2)	19(2)	12(2)	1(2)	0(1)	1(2)
N(8)	6(2)	19(2)	19(2)	6(2)	−2(2)	0(2)
N(9)	9(2)	23(2)	13(2)	3(2)	2(2)	2(2)
N(10)	17(2)	30(2)	28(2)	16(2)	8(2)	1(2)
N(11)	16(2)	17(2)	18(2)	6(2)	0(2)	1(2)
N(12)	19(2)	15(2)	19(2)	7(2)	0(2)	3(2)
O(1)	12(2)	26(2)	18(2)	4(1)	5(1)	5(1)
O(2)	23(2)	29(2)	33(2)	13(2)	−2(1)	2(2)
O(3)	34(2)	27(2)	79(3)	27(2)	0(2)	6(2)
O(4)	19(2)	53(2)	43(2)	16(2)	0(2)	4(2)
O(5)	13(2)	34(2)	17(2)	3(1)	2(1)	1(1)
O(6)	9(2)	23(2)	22(2)	6(1)	3(1)	2(1)
O(7)	21(2)	32(2)	40(2)	18(2)	7(2)	9(2)
O(8)	197(6)	44(3)	36(3)	11(2)	46(3)	22(3)
O(9)	17(2)	83(3)	110(3)	77(3)	−11(2)	2(2)

O(10)	14(2)	38(2)	21(2)	4(1)	2(1)	5(2)
O(11)	15(2)	44(2)	17(2)	8(1)	3(1)	2(2)
O(12)	18(2)	32(2)	20(2)	−1(1)	0(1)	0(2)
O(13)	11(2)	30(2)	18(2)	3(1)	0(1)	1(1)
O(14)	16(2)	40(2)	16(2)	8(1)	3(1)	5(2)
O(15)	17(2)	28(2)	20(2)	0(1)	0(1)	−1(1)
O(16)	7(2)	34(2)	26(2)	2(1)	0(1)	4(1)
O(17)	33(2)	32(2)	25(2)	7(1)	1(2)	−11(2)

Table S17. Hydrogen coordinates (× 10^4) and isotropic displacement parameters ($Å^2$ × 10^3) for **3.**

	x	y	z	U(eq)
H(1A)	5149	9340	1102	17
H(6A)	5278	8326	−467	21
H(7A)	975	7186	−2795	22
H(8A)	2242	6413	−3953	23
H(10A)	5479	7245	−2142	23
H(11A)	5695	6404	−3756	32
H(11B)	4519	6345	−4489	32
H(11C)	4623	5417	−4078	32
H(13A)	3475	12248	4884	24
H(13B)	4894	12327	5366	24
H(14A)	3416	11497	6058	43
H(14B)	4376	10686	5575	43
H(14C)	2956	10610	5096	43
H(15A)	−1999	9190	1232	48
H(15B)	−1054	8403	689	48
H(15C)	−1063	9537	594	48
H(16A)	10229	4475	1158	15
H(21A)	10288	3567	−440	18
H(22A)	5843	2191	−2615	24
H(23A)	7057	1423	−3821	22
H(25A)	10408	2540	−2151	22
H(26A)	10521	1633	−3765	31
H(26B)	9257	1424	−4464	31
H(26C)	9562	588	−3977	31
H(28A)	8700	7331	4991	22
H(28B)	10112	7374	5470	22
H(29A)	8542	6638	6195	36
H(29B)	9505	5801	5769	36
H(29C)	8106	5711	5254	36
H(2N)	5260	10253	2525	23
H(3N)	874	8196	−1143	22
H(4N)	5465	11192	4027	26
H(7N)	10371	5251	2606	22
H(8N)	5841	3161	−1000	23
H(9N)	10600	6176	4065	23
H(5O)	−421	9949	2297	34
H(10O)	4443	5105	1417	39
H(10P)	4763	5700	2351	39
H(17O)	2646	3860	931	48
H(17P)	3097	3853	157	48

Table S18. Cytotoxicity in A375 cancer cells.

	IC50(μM) ± S.D			
Compounds	12 h	24 h	48 h	72 h
cisplatin	21.2 ± 4.3	8.3 ± 2.1	3.3 ± 0.7	1.3 ± 0.6
1	19.3 ± 3.6	5.5 ± 2.1	1.1 ± 0.4	0.6 ± 0.2
2	5.3 ± 1.1	2.5 ± 0.6	0.6 ± 0.1	0.027 ± 0.005
3	7.1 ± 1.9	3.0 ± 0.8	0.7 ± 0.1	0.031 ± 0.001
cisplatin	21.2 ± 4.3	8.3 ± 2.1	3.3 ± 0.7	1.3 ± 0.6

IC_{50} values were calculated by four parameter logistic model ($p < 0.05$). Cells ($5 \times 10^3 \cdot mL^{-1}$) were treated for 12, 24, 48 and 72 h with increasing concentrations of tested compounds dissolved in DMSO. Cytotoxicity was assessed by MTT test. S.D. = standard deviation. Cells ($5 \cdot \times 10^3 \cdot mL^{-1}$) were treated for 12, 24, 48 and 72 h with increasing concentrations of tested compounds dissolved in DMSO. Cytotoxicity was assessed by MTT test.

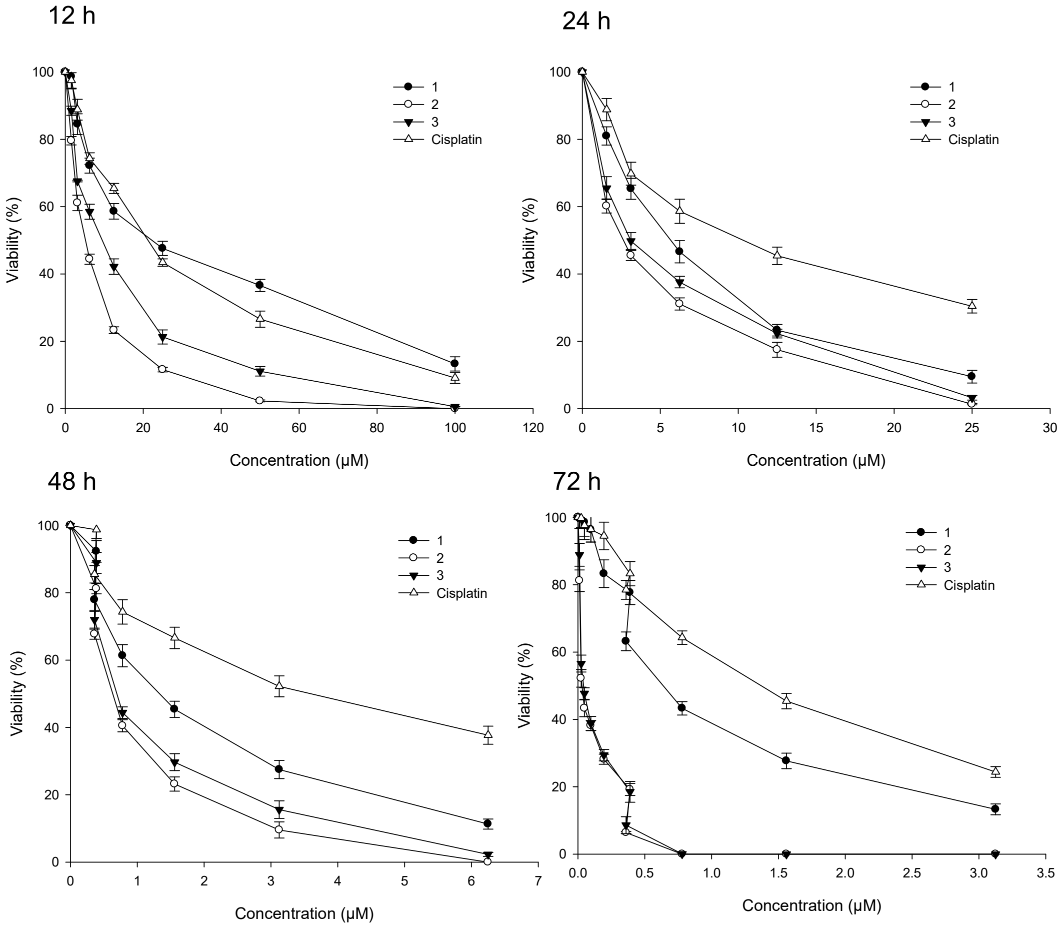

Figure S17. Dose-response curves for cell viability assessed in A375 cells.
